# Benchmarking the MinION: Evaluating long reads for microbial profiling

**DOI:** 10.1038/s41598-020-61989-x

**Published:** 2020-03-20

**Authors:** Robert Maximilian Leidenfrost, Dierk-Christoph Pöther, Udo Jäckel, Röbbe Wünschiers

**Affiliations:** 1 Department of Biotechnology and Chemistry, Mittweida University of Applied Sciences, Technikumplatz 17, 09648 Mittweida, Germany; 2 Unit for Biological Agents, Federal Institute for Occupational Safety and Health, Nöldnerstr. 40-42, 10317 Berlin, Germany

**Keywords:** Applied microbiology, Metagenomics

## Abstract

Nanopore based DNA-sequencing delivers long reads, thereby simplifying the decipherment of bacterial communities. Since its commercial appearance, this technology has been assigned several attributes, such as its error proneness, comparatively low cost, ease-of-use, and, most notably, aforementioned long reads. The technology as a whole is under continued development. As such, benchmarks are required to conceive, test and improve analysis protocols, including those related to the understanding of the composition of microbial communities. Here we present a dataset composed of twelve different prokaryotic species split into four samples differing by nucleic acid quantification technique to assess the specificity and sensitivity of the MinION nanopore sequencer in a blind study design. Taxonomic classification was performed by standard taxonomic sequence classification tools, namely Kraken, Kraken2 and Centrifuge directly on reads. This allowed taxonomic assignments of up to 99.27% on genus level and 92.78% on species level, enabling true-positive classification of strains down to 25,000 genomes per sample. Full genomic coverage is achieved for strains abundant as low as 250,000 genomes per sample under our experimental settings. In summary, we present an evaluation of nanopore sequence processing analysis with respect to microbial community composition. It provides an open protocol and the data may serve as basis for the development and benchmarking of future data processing pipelines.

## Introduction

Sequencing of environmental DNA has established itself as a means to overcome the limitations of cultivation and to understand the composition and dynamics of microbial communities^1–3^. Throughout the past two decades, sequencing technologies have continually experienced a decrease in cost and increase in output. As such and due to its wide availability, next-generation (also known as second-generation) DNA sequencing is currently the major technology^2,4,5^. Yet, a limitation of second generation DNA sequencing remains: its short reads. While first generation Sanger sequencing yields up to 1,000 basepairs (bp), second-generation methods (e.g. Illumina MiSeq) are limited to app. 300 bp. Nanopore-based sequencing is a third-generation sequencing method enabling deciphering of nucleic acids exceeding several thousand basepairs. The technology is generally applicable to a wide variety of purposes in basic and applied research in all kingdoms, as well as to clinical and life science applications^6–8^. Sequencing devices employing this technology are currently distributed through Oxford Nanopore Technologies and the technology as a whole is, as of today, under active development. This is of particular interest since nanopore sequencing, or long-read sequencing, has previously been labelled as error prone^9^, although more recent advances brought improvements to both chemistry and data processing (e.g. Brown, Nanopore Community Meeting Presentation 2018;^10^). On the other hand, single molecule sequencing using nanopores is generating long reads, which are, among other reasons, of interest in elucidating microbial diversity^11^. Other advantages of the first available sequencer model, the MinION, also compared to its larger siblings GridION and PromethION, are the lower initial investment and its mobility allowing for direct field studies^12–14^. Since the introduction of the MinION, several studies have been presented concerning its performance. However, those were not taking advantage of amplification-free sequencing and employed - to date - previous^15–18^ or other^19^ versions of the sequencing chemistry. Aforementioned ongoing development of the technology as a whole necessitates new and frequent revisions and updates to sequencing protocols and downstream data processing. This is also the case with the development of bioinformatics pipelines and the design of tools^20^. For this purpose, suitable datasets for rigorous testing are required. Recently, such datasets have been supplied for GridION and PromethION sequencers using commercially available standards^21^. We investigated four mixed microbial DNA samples differing by the employed DNA-quantitation technique and their composition using the MinION sequencer. The samples were composed of DNA covering up to five orders of magnitude in genome amounts from twelve bacterial species. The aim of the study includes an establishment of a suitable classification pipeline and an assessment of the accuracy of the MinION in samples with unknown microbial composition.

## Results and discussion

### Raw dataset description

Using the MinION DNA-sequencing platform we generated app. 809k reads in Fast5 file format, equal to an estimate of 8.15 Gbp in a single run within 36 hours (see Supplementary Fig. S1,S2). We could observe increased yield for each pore group switch (a.k.a remux), and output of constant quality on a uniform read length distribution for our sequencing run (see Supplementary Figs. S3–S5). Approximately 807k reads equal to 7.06 Gbp were successfully basecalled and demultiplexed generating an overall yield of 662k reads equivalent to 6.17 Gbp for downstream analysis. Samples one to four, corresponding to the four barcodes used, are composed of app. 142k (#1, heterogeneous sample quantified by ddPCR), 262k (#2, heterogeneous sample quantified by Qubit), 110k (#3, equimolar sample quantified by ddPCR) and 148k (#4, equimolar sample quantified by Qubit) reads, respectively. A total of only four reads were not properly demultiplexed by Porechop, i.e. assigned to a barcode not present in the library. A total of app. 140k reads were demultiplexed as “unclassified” by Porechop, i.e. not assigned to any barcode. All reads not assigned to barcodes #1, #2, #3 or #4 (corresponding to the four samples) were discarded and thus excluded from downstream analysis (Table 1).

**Table 1:** Yield (reads and bases), read length and mean quality presenting the output of the 36 h MinION sequencing run, after basecalling (Albacore) and adapter removal (Porechop).

Sample	Assignment	Reads	Yield [MBp]	Read length Mean [Bp]	Mean read quality [Q]	Read length N50 [Bp]
	**Basecalled:**					
1 (heterogenous, adjusted by ddPCR)	Barcode 01	143,672	1,362.69	9,485	12.8	14,312
2 (heterogenous, adjusted by Qubit)	Barcode 02	263,786	2,621.79	9,939	12.8	15,140
3 (equimolar, adjusted by ddPCR)	Barcode 03	111,370	956.20	8,586	12.8	13,835
4 (equimolar, adjusted by Qubit)	Barcode 04	150,965	1,372.85	9,094	12.8	14,385
	Unclassified	137,175	748.90	5,459	7.7	
	Misclassified	7	0.09	10,534	8.9	
	**Porechopped:**					
1 (heterogenous, adjusted by ddPCR)	Barcode 01	142,008	1,331.32	9,375	12.9	14,245
2 (heterogenous, adjusted by Qubit)	Barcode 02	261,833	2,571.89	9,823	12.9	15,074
3 (equimolar, adjusted by ddPCR)	Barcode 03	109,948	931.52	9,472	12.9	13,777
4 (equimolar, adjusted by Qubit)	Barcode 04	148,392	1,334.56	8,994	12.9	14,329
	Unclassified	140,464	800.95	5,703	8.0	
	Misclassified	4	0.06	15,012	11.0	

A clear drop in quality for un- and misclassified reads is observable as compared to correct assignment. Assigned Barcodes 1 to 4 match samples 1 to 4 (heterogeneous and equimolar adjusted by either ddPCR or Qubit). Statistics generated with NanoPlot, based on the sequencing_summary (Basecalled) and the individual fastq bins after porechopping.

### Data classification and validation

**Figure 1:**
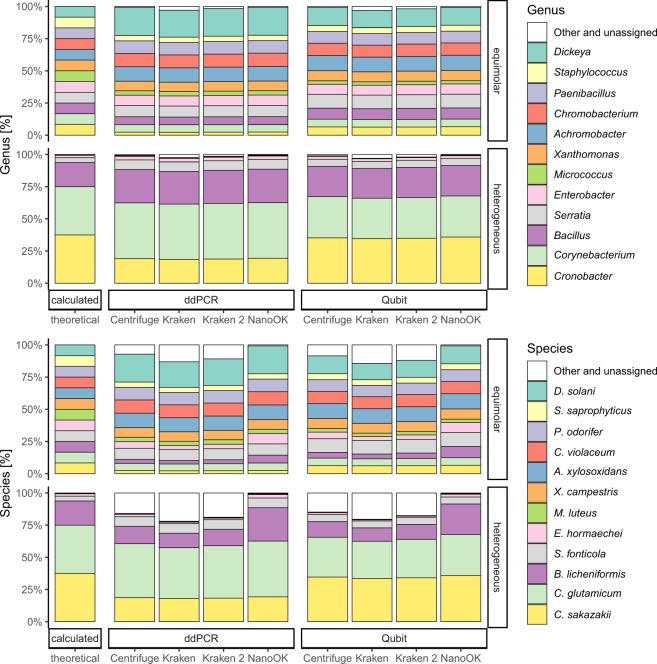
Centrifuge, Kraken and Kraken 2 classification results on genus and species level for equimolar (sample/barcode 3, adjusted by ddPCR and sample/barcode 4, adjusted by Qubit) and heterogeneously concentrated samples (sample/barcode 1, adjusted by ddPCR and sample/barcode 2, adjusted by Qubit) of 12 target strains. Theoretical values and validation by NanoOK (alignments with minimap2) are given for comparison.

The use of Centrifuge with nanopore read datasets has been demonstrated before^22,23^. The application of Kraken and Kraken2 on nanopore data has also been described, albeit within different experimental settings, such as the taxonomic classification of reads of well characterized isolates^24^ or the taxonomic classification of complete assemblies^21^. Taxonomic classification performed by either, Centrifuge, Kraken or Kraken2 allowed for the heterogeneously concentrated samples (samples #1 and #2, adjusted by ddPCR and Qubit, respectively) an initial choice of five out of twelve strains based on the available Krona plots (see Supplementary data S1). For the samples with equimolar genomic concentration (samples #3 and #4), a selection of twelve strains was immediately possible (Fig. 1). Generally, despite the differences in the underlying software and databases/indices, we could observe substantial agreement^25^ between the results obtained from Kraken, Kraken 2 and Centrifuge with their respective databases as tested by Fleiss Kappa (lowest 0.778, highest 0.931).

Quantitation by ddPCR delivers slightly different results than quantitation by fluorometry such as Qubit^26,27^. This is due to e.g. different basepair compositions, staining efficiencies or denaturation of DNA prior to droplet generation. Thus, we investigated, if the slight difference between these two quantitation approaches (Qubit vs. ddPCR) were also determinable by nanopore-based DNA-sequencing. Indeed, differences in quantitation, which resulted in different volumes necessary for sample preparations, corresponded to different amount of reads for that specific organism to the same extent (see Supplementary Fig. S6).

Unblinding the ground truth to the sequencing laboratory revealed a correct, that is true positive, selection of all twelve strains in samples of equimolar genomic concentration, as well as a correct selection of five out of twelve strains in the two samples with different genomic concentration. The five strains selected from the heterogeneously concentrated samples made up 99.38% of the genomes calculated to be available in the actual samples of different genomic concentration. This corresponds to a concentration of 2.5 million to 50 million genomes per species and sample. Notably, read classification matching the ground truth on genus level was possible for up to 99.27% (Centrifuge) between all samples, whereas read classification matching the ground truth on species level was up to 92.78% (Centrifuge) across all samples (Table 2). Generally, accuracy and deviation metrics (root mean squared deviation (RMSD) and mean absolute error (MAE)) on genus level were better than on species level. Comparing Centrifuge, Kraken and Kraken2 running their precompiled databases/indices, Centrifuge was able to assign the highest fraction of reads to the theoretically expected genera and species across all samples. Also, Centrifuge performed best with respect to both measures of deviation (RMSD, MAE), whereas Kraken 2 was superior over Kraken. However, beyond the accuracy of each classifier, computational aspects need to be considered. Especially, when limited computational resources are available, such as in field applications, Kraken 2 offers superior processing speed and lower memory consumption compared to Centrifuge and Kraken^28^.

**Table 2:** Taxonomic assignment accuracy and corresponding deviation metrics (RMSD and MAE) for Centrifuge, Kraken and Kraken 2 across all four samples, on genus and species level, respectively.

Sample	Software	Genus			Species		
		Accuracy (%)	RMSD	MAE	Accuracy (%)	RMSD	MAE
1 (heterogenous, adjusted by ddPCR)	Centrifuge	**99.27**	**0.0585**	**0.0286**	**84.10**	**0.0718**	**0.0374**
	Kraken	97.60	0.0589	0.0293	77.98	0.0847	0.0426
	Kraken 2	98.57	0.0587	0.0290	81.18	0.0775	0.0398
2 (heterogenous, adjusted by Qubit)	Centrifuge	**98.96**	**0.0221**	**0.0123**	**85.15**	**0.0494**	**0.0256**
	Kraken	97.06	0.0238	0.0140	79.54	0.0669	0.0341
	Kraken 2	98.06	0.0228	0.0132	82.47	0.0576	0.0296
3 (equimolar, adjusted by ddPCR)	Centrifuge	**99.26**	**0.0469**	**0.0322**	**92.78**	**0.0530**	**0.0417**
	Kraken	97.10	0.0459	0.0332	86.89	0.0600	0.0469
	Kraken 2	98.37	0.0464	0.0326	89.15	0.0568	0.0450
4 (equimolar, adjusted by Qubit)	Centrifuge	**99.08**	**0.0287**	**0.0224**	**91.49**	**0.0396**	**0.0332**
	Kraken	96.91	0.0290	0.0234	85.75	0.0518	0.0390
	Kraken 2	98.14	0.0287	0.0228	88.03	0.0466	0.0368

Centrifuge has highest accuracy for all samples, genus level classification metrics are superior compared to corresponding species level classification.

Precision and recall per species and genus reached generally high values on read level (see Supplementary Table S3, S4). For genera with very low abundancy, drops in precision could be observed (see Supplementary Table S3). Reads wrongly classified on species level were, e.g., attributable to close relatives, such as *Bacillus* species to *Bacillus licheniformis*, *Enterobacter cloacae* to *Enterobacter hormaechei*, *et cetera*, or exhibited differences in read abundancy as compared to true positive hits, which is similar to findings reported by Deshpande *et al*.^19^ despite a different sequencing and analysis approach. This is also reflected by the lower values of recall for these species on read level (see Supplementary Table S4). The necessity for accurate databases and unified nomenclature is discussed elsewhere^29–32^ and has been shown to affect classification of nanopore data^18^. These results indicate that classification is, as of yet, more reliable on genus level than on species level.

**Figure 2:**
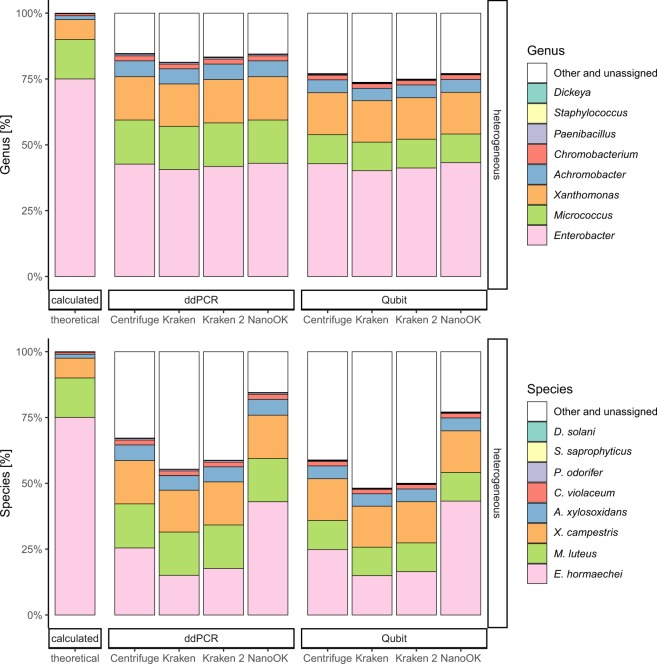
In silico complexity reduction of the samples with heterogeneous genomic concentration (sample/barcode 1, adjusted by ddPCR and sample/barcode 2, adjusted by Qubit) allows reliable detection of further strains down to an original genomic concentration around 25,000 genomes. Strains adjusted to the range of 500 to 5,000 genomes could not be reliably detected. Theoretical values and validation by NanoOK (alignments with minimap2) are given for comparison.

Serendipitously, rerunning the classification process after the removal of four most abundant initially selected strains from the read data allowed the additional selection and thus classification of four strains down to app. 25,000 to 500,000 genomes per sample, using Krona plots. The remaining three strains adjusted to the range of 500 to 5,000 genomes per sample could not be reliably retrieved from the two samples with heterogeneous genomic concentrations (Fig. 2). Their presence was obfuscated by the filter process, i.e. they were as abundant as falsely classified reads and, subsequently, a clear discrimination allowing selection and classification was impossible. With the experimental settings and proceeding as described here, this suggests a dynamic range of detection and viable classification between 250 and 500,000 genomes/µl of initial DNA input, corresponding to a range of 25,000 to 50 million genomes from material obtained from microbial communities of low diversity from the MinION. The range reported here is similar to the findings of Nicholls *et al*.^21^.

**Figure 3:**
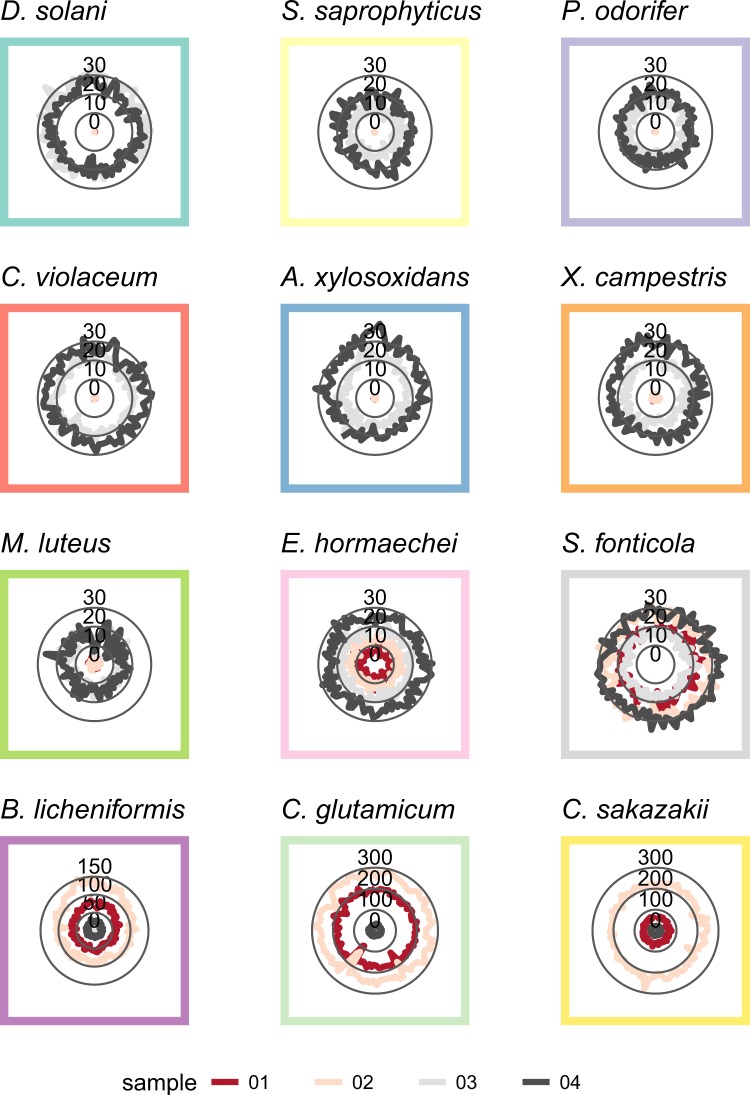
Sequenced coverage per sample for each of the twelve identified strains in the community. Data based on NanoOK analysis.

These results showed good consistency with a) the output from the NanoOK analysis by direct comparison (Table 3, see Supplementary Table S5), where at least 99.21% of all available reads could be aligned to selected references and b) the theoretical expectation. Moreover, mean coverages reported by NanoOK indicate potential for *de novo* genome assemblies (Fig. 3). Full genomic coverage realistically permitting *de novo* assembly was achieved for strains down to a concentration of 250,000 genomes per sample (see Supplementary Table S5). At comparable sequencing times, we anticipate the concentration level required to achieve full genomic coverage to be even lower for libraries that are not multiplexed.

**Table 3:** NanoOK alignment statistics for each sample. Alignments were performed against RefSeq genomes.

Sample	Reads (total)	Reads with alignments	[%]	Reads without alignments	[%]	Read length mean [bp]	Read length N50 [bp]
1 (heterogenous, adjusted by ddPCR)	142,008	141,332	99.52	676	0.48	9,375	14,245
2 (heterogenous, adjusted by Qubit)	261,833	260,540	99.51	1,293	0.49	9,823	15,074
3 (equimolar, adjusted by ddPCR)	109,948	109,148	99.27	800	0.73	8,472	13,777
4 (equimolar, adjusted by Qubit)	148,392	147,217	99.21	1,175	0.79	8,994	14,329

Despite the error rates currently accompanying MinION sequencing, these results clearly illustrate the viability and possibilities of long reads for direct taxonomic classification and abundance estimation with currently available bioinformatics pipelines.

## Conclusion

We present a MinION DNA sequence read dataset to facilitate the Nanopore community to improve and develop new bioinformatics pipelines aimed at the understanding of microbial diversity. Continual benchmarking using updated sequencing methods and chemistries in metagenome analyses is required^32^. With the presented detailed methodology, as a whole, this study follows the FAIR Guiding Principles^33^ for scientific data management and stewardship by contributing (F)indable and (A)ccessible data under bioproject accession PRJNA545964 and corresponding signal level data^34^ that is (R)eusable for the fast-paced development of third generation sequencing and downstream bioinformatics in a metagenomics context.

Based on the dataset, we present a simple and straightforward analysis pipeline to investigate the composition of microbial communities. Given our experimental approach we were able to achieve highly accurate taxonomic classification of low abundant (25,000 genomes/sample) organisms to at least genus level. Full genomic coverage was achieved for species with an abundancy of 250,000 genomes per sample and sufficient coverage for *de novo* assembly could be obtained.

While there is no standardized approach for the characterization of bacterial communities, molecular tools are considered powerful to gain knowledge and insight into these^35,36^, and nanopore sequencing is no exception to this point. In summary, the presented benchmark provides insight into nanopore data and data processing for the taxonomic classification of microbial communities. Hence, this study contributes to the toolsets and development of processing pipelines available to elucidate microbial diversity.

## Material and methods

**Figure 4:**
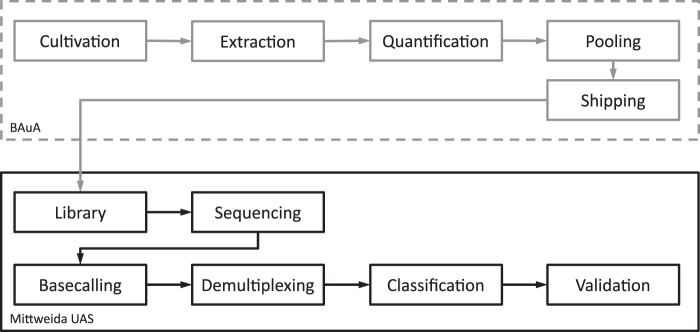
Overall study design and process workflow. Part One (grey), mock community creation, was performed by the Unit for Biological Agents, Federal Institute for Occupational Safety and Health (BAuA, Berlin). Part Two (black), sequencing and data processing was performed by Wünschiers Group, University of Applied Sciences Mittweida (Mittweida UAS).

The overall experimental design is setup as follows: Bacteria cultivation, DNA extraction, quantification and creation of mock samples were performed by the Unit for Biological Agents, Federal Institute for Occupational Safety and Health (BAuA). Samples were shipped to the sequencing team (Mittweida UAS). The sequencing team performed library preparation, sequencing and downstream processing unaware of the samples’ actual respective compositions (Fig. 4).

### Sample cultivation and preparation

DNA from twelve bacterial strains was extracted to form a mock community sample (Table 4) for benchmarking the MinION sequencing platform using the following criteria: (A) Each strain is the type strain of the bacterial species and is available from the Leibniz Institute DSMZ - German Collection of Microorganisms and Cell Cultures GmbH (DSMZ), the National Collection of Type Cultures (NCTC) or the American Type Culture Collection (ATCC). (B) Each strain has a reference sequence deposited at the National Center for Biological Information (NCBI). (C) Each strain has several assemblies of the same species available at the NCBI. (D) The sequencing laboratory is blind to both, the selection itself and the actual composition of the strains selected.

**Table 4:** Strain community overview: Overview of the strains selected to compose the microbial community with accessions and genomic specifications shown.

Species	Strain ID	Accession			Refseq ID	Size	GC (%)	16 S rRNA-genes	Gram	genomes in ranged sample	
		DSMZ	ATCC	NCTC		Mbp		n		n	%
***Bacillus licheniformis***^***T***^	Gibson 46	13	14580	10341	NC_006270.3	4.22	46.19	7	Pos	2,50E + 07	18.7500
***Xanthomonas campestris***^***T***^	P25	3586	33913	—	NC_003902.1	5.08	65.07	2	Neg	2,50E + 05	0.1875
***Staphylococcus saprophyticus subsp. saprophyticus***^***T***^	S-41	20229	15305	13634	NC_007350.1	2.52	33.24	6	Pos	2,50E + 03	0.0018
					NC_007351.1	0.038	30.75				
					NC_007352.1	0.022	31.34				
***Corynebacterium glutamicum***^***T***^	534	20300	13032	—	NC_003450.3	3.31	53.81	6	Neg	5,00E + 07	37.5000
***Micrococcus luteus***^***T***^	2665	20030	4698	2665	NC_012803.1	2.5	73.00	2	Pos	5,00E + 05	0.3750
***Enterobacter hormaechei subsp. Steigerwaltii***^***T***^	EN-562	16691	—	—	NZ_CP017179.1	4.78	55.55	8	Neg	2,50E + 06	1.8750
***Cronobacter sakazakii***^***T***^	CDC 4562–70	4485	29544	11467	NZ_CP011047.1	4.51	56.71	4	Neg	5,00E + 07	37.5000
					NZ_CP011048.1	0.093	57.02				
					NZ_CP011049.1	0.004	54.88				
					NZ_CP011050.1	0.053	50.07				
***Achromobacter xylosoxidans subsp. xylosoxidans***^***T***^	KM543	2402	27061	10807	NZ_LN831029.1	6.81	67.38	3	Neg	5,00E + 04	0.0375
***Paenibacillus odorifer***^***T***^	TOD45	15391	BAA-93	—	NZ_CP009428.1	6.81	44.21	10	Pos	5,00E + 03	0.0037
***Chromobacterium violaceum***^***T***^	MK	30191	12472	9757	NC_005085.1	4.75	64.83	8	Neg	2,50E + 04	0.0188
***Dickeya solani***^***T***^	IPO2222	28711	—	—	NZ_CP015137.1	4.92	56.21	7	Neg	5,00E + 02	0.0004
***Serratia fonticola***^***T***^	CUETM 77.165	4576	29844	12965	NZ_CP011254.1	6	53.61	7	Neg	5,00E + 06	3.7500

Bacteria were grown overnight as follows: *Dickeya solani*^T^ (Todd-Hewitt + 0,5% yeast extract (THY), 28 °C), *Serratia fonticola*^T^ (DSMZ-Medium 1, 28 °C), *Bacillus licheniformis*^T^ (DSMZ-Medium 1, 37 °C), *Corynebacterium glutamicum*^T^ (THY, 28 °C), *Micrococcus luteus*^T^ (THY, 28 °C), *Cronobacter sakazakii*^T^ (DSMZ-Medium 1, 28 °C), *Achromobacter xyloxidans* subsp. *xyloxidans*^T^ (DSMZ-Medium 1, 28 °C), *Paenibacillus odorifer*^T^ (DSMZ-Medium 1, 28 °C), *Chromobacterium violaceum*^T^ (DSMZ-Medium 1, 28 °C), *Enterobacter hormaechei* subsp. *steigerwaltii*^T^ (CASO, 37 °C), *Staphylococcus saprophyticus* subsp. *saprophyticus*^T^ (DSMZ-Medium 92, 37 °C) and *Xanthomonas campestris*^T^ (DSMZ-Medium 1, 28 °C). DNA of 1 ml of the cell suspension derived from liquid culture or resuspended colonies in PBS was extracted using a modified protocol of the GenElute Plant Genomic DNA Miniprep Kit (Sigma Aldrich,^37^). DNA concentrations were quantified using the Qubit BR assay in a Qubit 1.0 fluorometer according to the manufacturer’s protocol. Subsequently, ddPCR targeting the 16 S rRNA-gene was conducted with app. less than 40,000 target genes according to the manufacturer’s instructions (Bio-Rad) using the ddPCR Supermix for Probes (no dUTP). Final concentrations of oligonucleotides were 0.4 pmol/µL 1055Falt (ATGGRTGTCGTCAGCT), 0.2 pmol/µL 1392 R (ACGGGCGGTGTGTAC) and 0.1 pmol/µL 1115IB (FAM-CAACGAGCG-ZEN-CAACCC-3IABkFQ) adopted from Rothrock *et al*.^38^. Droplet generation was conducted according to manufacturer’s instructions in a QX200 Droplet Generator and amplified in a T100 Thermal Cycler. PCR conditions were initial denaturation at 95 °C for 10 min, and 30 cycles of denaturation at 95 °C for 30 s, annealing at 57 °C for 45 s, extension at 72 °C for 45 s with a ramp rate of 1 °C/s, followed by a final extension at 98 °C for 10 min and cooling to 12 °C. Droplet evaluation was performed in a QX200 Droplet Reader with QuantaSoft-Software.

Based on Qubit and ddPCR quantitation, the nucleic acids were adjusted to different genomic concentrations ranging from 5 to 5*10^5^ genomes/µl (samples #1 and #2, corresponding to sequencing library barcodes #1 and #2), or to equimolar genomic concentration of 5*10^4^ genomes/µl (samples #3 and #4 corresponding to sequencing library barcodes #3 and #4).

Samples were shipped on ice by public postal services.

### Library preparation and sequencing

A sequencing library was prepared according to manufacturer’s instructions. The Ligation Sequencing Kit (SQK-LSK108, Oxford Nanopore Technologies (ONT)) and the Native Barcoding Expansion 1–12 kit (EXP-NBD103, ONT), barcoding each of the samples (barcodes #1, #2, #3, #4), were used with the following exceptions: Shearing times were prolonged and an optional FFPE DNA repair step (M6630, New England Biolabs (NEB)) was included. The incubation times during the end-repair/dA-tailing (E7546, NEB) were extended from five to 20 minutes for both, the 20 °C and 65 °C incubation steps. Qubit checkpoint measurements were performed according to the library preparation protocol (see Supplementary Table S1). Pooling of the barcoded samples was performed ‘as is’ instead of protocol-given ‘equimolar’. Sequencing was then performed on a R9.4 flowcell (FLO-MIN106, ONT, >1200 pores, see Supplementary Table. S2) with MinKNOW (version 2.1.12, ONT) at room temperature.

### Base calling and demultiplexing

Upon conclusion of sequencing, raw data in Fast5 file format were transferred to our server (4.17.2-1-ARCH, 20 cores with 2 threads each, 256 GB RAM) and basecalled using the Albacore software (version 2.0.2, ONT) with barcoding option. Subsequently, barcodes were removed from basecalled output and subsequently sorted utilizing Porechop (version 0.2.3, standard settings, https://github.com/rrwick/Porechop). Basecalled and demultiplexed sequencing data quality was assessed with NanoPack (version 1.13.0, https://github.com/wdecoster/NanoPlot)^39^.

### Data classification and validation

Taxonomic classification was performed with standard parameters (Centrifuge “-k 1”) on native reads using Centrifuge (precompiled index: “Bacteria, Archaea (compressed), 2018-4-15”)^22^, as well as Kraken (precompiled database: “DustMasked MiniKraken DB 8GB”)^40^ and Kraken2 (precompiled database: MiniKraken2_v1_8GB)^28^ and the results were visualized with Krona^41^ and R^42–45^.

The interactive and intuitive Krona visualization was used to manually select up to twelve bacterial strains. The corresponding genome reference sequences were obtained from NCBI Reference Sequence Database^46^ (accessed on 2018-07-31).

NanoOK (version 1.34)^47^ was utilized for an assessment of the read dataset against the selection of NCBI genome reference sequences, using minimap2 aligner (version 2.11)^48^. To create the minimap2 index, the reference sequences obtained from NCBI Reference Sequence Database were concatenated into a single FastA file.

Statistics and additional visualizations were computed with R^42–45,49,50^. We calculated the accuracy of the classification performed by Centrifuge, Kraken and Kraken 2 on each sample the proportion of reads assigned to the known input organism at the genus and species level out of the total number reads given any assignment at that rank^18^. To calculate a corresponding estimate of the accompanying error, the mean absolute error, as well as root mean squared deviation of classified to theoretically present fractions on genus and species level were computed. On read level, precision and recall for genus and species identification were computed^32^ for Centrifuge, Kraken and Kraken 2 *vs*. the results obtained from the NanoOK analysis, with precision being the proportion of reads classified correctly to reads classified and recall being the proportion of reads classified correctly to the reads from the NanoOK dataset, which was used as “ground truth”. All additional bioinformatics processing was performed in the Linux Bourne Again Shell (bash), using Samtools (version 1.9)^51^ and seqtk (version 1.3-r106, https://github.com/lh3/seqtk).

## Supplementary information

Supplementary information.

Supplementary information2.

Supplementary information3.

## Data Availability

The data sets supporting the results of this article are available in the under bioproject accession PRJNA545964 (https://www.ncbi.nlm.nih.gov/sra/PRJNA545964) and as Zenodo deposit 3600229 (10.5281/zenodo.3600229).
