# Supplementary material for: Benchmarking the MinION: Evaluating long reads for microbial profiling
Source: Sci Rep. 2020 Mar 20;10:5125. doi: 10.1038/s41598-020-61989-x (PMC7083898; doi:10.1038/s41598-020-61989-x)
Supplement: Supplementary file 1 — Supplementary information. [file 41598_2020_61989_MOESM1_ESM.pdf]

Supplementary information

to

Benchmarking the MinION: Evaluating long reads for microbial profiling

Robert Maximilian Leidenfrost[1*], Dierk-Christoph Pöther[2], Udo Jäckel[2] and Röbbe Wünschiers[1]

[1]Department of Biotechnology and Chemistry, Mittweida University of Applied Sciences, Technikumplatz 17, 09648 Mittweida, Germany

[2]Unit for Biological Agents, Federal Institute for Occupational Safety and Health, Nöldnerstr. 40-42, 10317 Berlin, Germany

[*]Corresponding author: robert.leidenfrost@hs-mittweida.de

Supplementary tables

Supplementary table S1: Qubit DNA concentration measurements at various checkpoints during library preparation. Please note that the volumina and thus total DNA mass per sample for each checkpoint differ.

Sample	Original [ng/µl]	DNA Repair [ng/µl]	End prep [ng/µl]	Barcode [ng/µl]	Final [ng/µl]
1	3.70	5.42	6.54	4.60	16.6
2	5.78	8.80	11.8	12.1	
3	2.38	3.66	5.84	4.36	
4	3.36	4.54	6.52	5.02	

Supplementary table S2: Flowcell mux scan data for pre-run QC and run. A pore count > 1200 indicates a healthy flowcell in good condition.

Mux	QC	Run
1	510	501
2	477	432
3	353	273
4	137	80
Sum:	1477	1286

Supplementary table S3: Precision and Recall calculated for each genus per sample on read level.

	1		2		3		4		
	Prec	Recall	Prec	Recall	Prec	Recall	Prec	Recall	
Corynebacterium	0.994	0.997	0.994	0.997	0.994	0.996	0.994	0.997	**Centrifuge**
Xanthomonas	0.995	0.997	0.989	0.998	0.996	0.996	0.995	0.997	
Chromobacterium	1.000	1.000	0.984	0.984	0.996	0.996	0.996	0.995	
Bacillus	0.999	0.994	0.999	0.994	0.998	0.992	0.998	0.992	
Staphylococcus	0.250	1.000	0.750	0.857	0.986	0.996	0.984	0.996	
Micrococcus	0.977	0.995	0.979	0.995	0.979	0.994	0.981	0.995	
Dickeya	0.714	1.000	0.778	1.000	0.987	0.994	0.984	0.992	
Paenicbacillus	0.750	1.000	0.600	1.000	0.999	0.997	0.998	0.997	
Cronobacter	0.998	0.983	0.999	0.982	0.999	0.972	0.999	0.980	
Serratia	0.994	0.997	0.988	0.997	0.997	0.997	0.996	0.996	
Enterobacter	0.993	0.994	0.981	0.995	0.997	0.991	0.998	0.992	
Achromobacter	0.979	0.986	0.988	0.994	0.998	0.990	0.999	0.990	
Corynebacterium	0.995	0.986	0.995	0.986	0.994	0.986	0.995	0.985	**Kraken**
Xanthomonas	0.995	0.977	0.989	0.986	0.997	0.978	0.996	0.980	
Chromobacterium	1.000	0.960	1.000	0.967	0.996	0.975	0.997	0.973	
Bacillus	0.999	0.973	0.999	0.970	0.998	0.974	0.999	0.969	
Staphylococcus	1.000	1.000	0.750	0.857	0.990	0.981	0.987	0.980	
Micrococcus	0.979	0.979	0.982	0.979	0.980	0.975	0.981	0.977	
Dickeya	1.000	1.000	1.000	1.000	0.988	0.958	0.985	0.955	
Paenicbacillus	1.000	1.000	1.000	1.000	0.999	0.984	0.999	0.983	
Cronobacter	0.999	0.962	0.999	0.961	1.000	0.948	0.999	0.959	
Serratia	0.999	0.988	0.999	0.984	0.999	0.985	0.999	0.983	
Enterobacter	0.994	0.980	0.990	0.975	0.999	0.974	0.999	0.971	
Achromobacter	1.000	0.958	1.000	0.970	0.999	0.973	0.999	0.970	
Corynebacterium	0.995	0.993	0.995	0.993	0.994	0.993	0.995	0.992	**Kraken 2**
Xanthomonas	0.990	0.997	0.989	0.987	0.997	0.988	0.996	0.989	
Chromobacterium	0.980	1.000	1.000	0.967	0.996	0.988	0.997	0.985	
Bacillus	0.999	0.988	0.999	0.986	0.998	0.986	0.999	0.986	
Staphylococcus	0.500	1.000	0.857	0.857	0.988	0.991	0.985	0.990	
Micrococcus	0.979	0.987	0.982	0.987	0.980	0.985	0.981	0.989	
Dickeya	1.000	1.000	0.500	1.000	0.987	0.980	0.985	0.975	
Paenicbacillus	1.000	1.000	0.900	1.000	0.999	0.991	0.999	0.990	
Cronobacter	0.999	0.972	0.999	0.970	1.000	0.961	0.999	0.967	

Serratia	0.999	0.993	0.999	0.991	0.999	0.993	0.999	0.991	
Enterobacter	0.990	0.988	0.974	0.984	0.998	0.984	0.997	0.983	
Achromobacter	1.000	0.986	0.988	0.994	0.999	0.982	0.999	0.982	

Supplementary table S4: Precision and Recall calculated for each species per sample on read level.

	1		2		3		4		
	**Prec**	**Recall**	**Prec**	**Recall**	**Prec**	**Recall**	**Prec**	**Recall**	
C. glutamicum	0.994	0.964	0.994	0.964	0.994	0.962	0.994	0.966	**Centrifuge**
X. campestris	0.995	0.995	0.989	0.995	0.996	0.993	0.995	0.993	
C. violaceum	1.000	0.980	1.000	0.984	0.996	0.993	0.996	0.991	
B. licheniformis	0.999	0.517	0.999	0.517	0.999	0.504	0.999	0.504	
S. saprophyticus	1.000	1.000	1.000	0.857	0.989	0.990	0.986	0.992	
M. luteus	0.977	0.995	0.979	0.995	0.979	0.994	0.981	0.995	
D. solani	0.833	1.000	0.778	1.000	0.987	0.984	0.985	0.982	
P. odorifer	1.000	1.000	1.000	1.000	0.999	0.996	0.998	0.995	
C. sakazakii	0.999	0.966	0.999	0.965	0.999	0.955	0.999	0.965	
S. fonticola	0.998	0.997	0.998	0.996	0.999	0.997	0.998	0.996	
E. hormaechei	0.996	0.653	0.990	0.651	0.999	0.643	0.999	0.649	
A. xylosoxidans	0.979	0.986	0.994	0.988	0.998	0.984	0.999	0.985	
C. glutamicum	0.995	0.903	0.994	0.901	0.993	0.904	0.995	0.907	**Kraken**
X. campestris	0.995	0.967	0.989	0.973	0.997	0.971	0.996	0.971	
C. violaceum	1.000	0.940	1.000	0.967	0.996	0.972	0.997	0.968	
B. licheniformis	0.999	0.438	0.999	0.440	0.999	0.432	0.999	0.430	
S. saprophyticus	1.000	1.000	1.000	0.857	1.000	0.949	0.998	0.948	
M. luteus	0.979	0.979	0.982	0.979	0.980	0.975	0.981	0.977	
D. solani	1.000	0.800	1.000	0.429	0.988	0.907	0.985	0.904	
P. odorifer	1.000	1.000	1.000	1.000	0.999	0.982	0.999	0.979	
C. sakazakii	0.999	0.937	0.999	0.935	1.000	0.917	0.999	0.933	
S. fonticola	0.999	0.987	0.999	0.984	0.999	0.984	0.999	0.983	
E. hormaechei	0.998	0.400	0.998	0.389	0.999	0.384	0.999	0.389	
A. xylosoxidans	1.000	0.930	1.000	0.964	0.999	0.960	0.999	0.957	
C. glutamicum	0.995	0.934	0.994	0.933	0.993	0.935	0.995	0.936	**Kraken 2**
X. campestris	0.995	0.992	0.991	0.982	0.997	0.982	0.996	0.981	
C. violaceum	1.000	0.940	1.000	0.951	0.996	0.980	0.997	0.977	
B. licheniformis	0.999	0.493	0.999	0.493	0.999	0.486	0.999	0.487	
S. saprophyticus	1.000	1.000	1.000	0.857	0.991	0.979	0.989	0.979	
M. luteus	0.979	0.987	0.982	0.987	0.980	0.985	0.981	0.989	
D. solani	1.000	1.000	0.833	0.714	0.987	0.936	0.985	0.932	
P. odorifer	1.000	1.000	1.000	1.000	0.999	0.989	0.999	0.988	
C. sakazakii	0.999	0.950	0.999	0.949	1.000	0.938	0.999	0.946	
S. fonticola	0.999	0.992	0.999	0.991	0.999	0.993	0.999	0.991	
E. hormaechei	0.999	0.454	0.993	0.442	0.998	0.435	0.999	0.436	
A. xylosoxidans	1.000	0.965	0.994	0.988	0.999	0.972	0.999	0.972	

Supplementary table S5: NanoOK statistics (number of reads, mean read length, coverage) per sample and strain. The amount of reads per sample and coverage per strain correspond to DNA mass input.

Sample	**Number of reads [n]**				**Mean read length [bp]**				**Coverage [fold]**			
	**1**	**2**	**3**	**4**	**1**	**2**	**3**	**4**	**1**	**2**	**3**	**4**
C. glutamicum	61,510	83,401	6,368	8,836	9,890.54	10,153.69	9,609.39	9,995.09	188.67	263.37	19.08	27.47
X. campestris	393	552	8,216	11,715	10,560.97	10,889.66	10,288.31	10,555.56	0.84	1.20	17.09	24.95
C. violaceum	50	61	11,292	14,189	8,004.34	8,323.84	8,541.45	8,531.67	0.09	0.11	20.69	26.00
B. licheniformis	36,887	61,975	6,814	12,877	7,069.18	7,232.38	6,816.54	6,990.67	62.81	108.10	11.28	21.75
S. saprophyticus	1	5	4,240	6,406	5,916.00	9,112.20	7,660.79	7,814.76	0.00	0.02	12.89	19.76
M. luteus	376	384	3,726	3,878	10,591.40	10,562.12	10,004.03	10,292.62	1.58	1.61	14.80	15.84
D. solani	5	7	23,821	20,593	5,061.60	2,005.29	5,326.41	5,454.40	0.01	0.00	26.31	23.27
P. odorifer	12	9	10,822	13,521	9,659.33	8,908.00	9,366.08	9,465.18	0.02	0.01	15.30	19.33
C. sakazakii	26,442	90,749	2,607	9,337	10,578.29	11,007.08	10,035.62	10,643.33	63.43	226.23	5.93	22.55
S. fonticola	10,652	14,306	9,532	15,864	10,920.12	11,488.14	10,627.34	11,029.19	19.90	28.17	17.37	30.02
E. hormaechei	3,911	5,698	8,840	11,753	10,644.79	10,659.59	10,029.36	10,179.91	8.92	13.03	19.08	25.71
A. xylosoxidans	142	168	12,518	17,495	9,954.82	8,820.14	9,534.07	9,882.30	0.21	0.22	17.98	26.01

Supplementary figures

Find the supplementary attached as supplementary_figures.zip file. Figure legends as follows:

Supplementary figure S1: Assembled screenshot of Read Length Histogram (bucket width 1620 B) and Cumulative Throughput as given by MinKNOW control software at the end of 36h of sequencing. The overall expected data estimate by MinKNOW was approx. 8.15 GBp.

Supplementary figure S2: Duty time screenshot as given by MinKNOW control software after approx. 36h of sequencing/end of run. The flow cell re-mux every 8 hours of runtime is evident. The high pore occupancy and good yield indicate a „good run“. There is no exact explanation for the accumulation of pores in „Possible multiple“ state. (Angelica Vittori, ONT, personal communication)

Supplementary figure S3: Summary statistics for assessing the quality of the sequencing run. Figures composed by Nanoplot (unaltered).

Supplementary figure S4: Read length histograms after log transformation per sample: #1: Sample 1 (heterogeneous, adjusted by ddPCR), #2: Sample 2 (heterogeneous, adjusted by Qubit), #3: Sample 3 (equimolar, adjusted by ddPCR), #4: Sample 4 (equimolar, adjusted by Qubit). Figures from Nanoplot.

Supplementary figure S5: Read lengths vs Average read qualities per sample: #1: Sample 1 (heterogeneous, adjusted by ddPCR), #2: Sample 2 (heterogeneous, adjusted by Qubit), #3: Sample 3 (equimolar, adjusted by ddPCR), #4: Sample 4 (equimolar, adjusted by Qubit). Figures from Nanoplot.

Supplementary figure S6: Comparison of reads from equimolar pooled samples (3 and 4) assigned on genus level per applied DNA volume either quantified by Qubit or ddPCR for each correctly assigned genus. Reads were assigned by Centrifuge, Kraken, Kraken 2 or NanoOK.

Supplementary data S1 – Krona plots

Find the data attached as supplementary_data_s1-Krona_plots.zip file.

Supplementary note – software commands

Albacore

read_fast5_basecaller.py -i [input] -t [threads] -s [output] -f FLO-MIN106 -k SQK-LSK108 -r --barcoding

NanoPlot

NanoPlot -t [threads] -o [output] --barcoded --format [fmt] --plots [plots] --summary [input]
NanoPlot -t [threads] -o [output] --format [fmt] --plots [plots] --fastq [input]

Porechop

porechop-runner.py -i [input] -o [output] --threads [threads]

Centrifuge

centrifuge -x [db] -U [input] -k 1 --report-file [report] -S [result] -p [threads]

Kraken

kraken --db [db] --threads [threads] --fastq-input --output [output] [input]

Kraken 2

kraken2 --db [db] --threads [threads] --output [output] [input]

Krona

cat [path_to_input] | cut –f [columns] > [path_to_output]
ktImportTaxonomy [path_to_input]

NanoOK

minimap2 -d [index] [reference]
nanook_split_reads.pl -i [input] -o [output]

```
nanook align –a/q -s [input] -r [reference_input] -templateonly -passonly -numthreads [threads] -aligner [algn]
nanook analyse –a/q -s [input] -r [reference_input] -templateonly -passonly -numthreads [threads] -aligner [algn]
```
