# Supplementary material for: Benchmarking the MinION: Evaluating long reads for microbial profiling
Source: Sci Rep. 2020 Mar 20;10:5125. doi: 10.1038/s41598-020-61989-x (PMC7083898; doi:10.1038/s41598-020-61989-x)
Supplement: Supplementary file 2 — Supplementary information2. [file 41598_2020_61989_MOESM2_ESM.zip › sample_barcode_1/centrifuge.html]

Javascript must be enabled to view this page.

members
magnitude
magnitudeUnassigned
count
unassigned
taxon
rank

BC1\_k1\_centrifuge\_results

142009
1
node0.members.0.js

node1.members.0.js
511

superkingdom
2
141493
node2.members.0.js
11

node3.members.0.js
6
42587
1224
phylum

6
class
28211

204457
order
1

1
41297
family

1434046
genus
1

266812
species
1
node8.members.0.js

1
204441
order

family
433
1

1
434
genus

node12.members.0.js
1
446692
species

356
order
4

41294
family
1

1
genus
85413

1
species
1842539
node16.members.0.js

45401
family
1
node17.members.0.js

family
119045
1

node19.members.0.js
1
407
genus

family
82115
1

no rank
227290
1

genus
379
1

node23.members.0.js
species
1571470
1

2
class
1553900

2
213481
order

213483
family
2

genus
958
2

959
species
2
node28.members.0.js

node29.members.0.js
7
42372
1236
class

order
91347
41936
node30.members.0.js
56

8
family
1903412

genus
568
1

node33.members.0.js
569
species
1

7
635
genus

7
species
67780
node35.members.0.js

1
family
1903416

82984
genus
1

82985
species
1
node38.members.0.js

2
1903414
family

2
genus
626
1
node40.members.0.js

1
40576
species
node41.members.0.js

family
1903411
10696

2
node43.members.0.js
613
genus
10694

28151
species
1
node44.members.0.js

node45.members.0.js
species
614
1

48
species
615
node46.members.0.js

node47.members.0.js
10642
47917
species

2
629
genus

node49.members.0.js
1
29485
species

1
species
630
node50.members.0.js

family
1903410
7

7
204037
genus

node53.members.0.js
6
species
1089444

node54.members.0.js
204038
species
1

31153
543
family
136
node55.members.0.js

33
570
genus
5
node56.members.0.js

node57.members.0.js
species
2026240
1

14
573
species
node58.members.0.js

species
1905288
2
node59.members.0.js

node60.members.0.js
9
571
species

node61.members.0.js
1134687
species
2

1330547
genus
1

species
283686
1
node63.members.0.js

2
genus
158483

2
158822
species
node65.members.0.js

6
genus
544

6
species group
1344959

546
species
6
node68.members.0.js

413496
genus
26962
149
node69.members.0.js

species
28141
26505
node70.members.0.js

node71.members.0.js
230
413503
species

species
535744
46
node72.members.0.js

6
species
1163710
node73.members.0.js

node74.members.0.js
5
413497
species

413502
species
15
node75.members.0.js

6
species
413501
node76.members.0.js

genus
1048757
1

1048758
species
1
node78.members.0.js

83654
genus
2

2
species
1920116
node80.members.0.js

1330546
genus
1

1
species
61647
node82.members.0.js

genus
561
68

node84.members.0.js
1
208962
species

node85.members.0.js
562
species
66

node86.members.0.js
564
species
1

6
191675
no rank

6
no rank
36866

species
1920109
1
node89.members.0.js

node90.members.0.js
species
2066051
5

160674
genus
2

2
species
54291
node92.members.0.js

genus
590
12

node94.members.0.js
species
28901
12

3
579
genus

3
61648
species
node96.members.0.js

node97.members.0.js
183
genus
547
3913

2051905
species
30
node98.members.0.js

3700
354276
species group

2
species
208224
node100.members.0.js

species
550
1131
node101.members.0.js

69218
species
1
node102.members.0.js

node103.members.0.js
1915310
species
2

158836
species
2562
node104.members.0.js
2556

node105.members.0.js
1
1812934
subspecies

node106.members.0.js
1296536
subspecies
5

species
61645
2
node107.members.0.js

5
genus
620

5
species
622
node109.members.0.js

1903409
family
13

genus
2100764
2

2
species
665914
node112.members.0.js

4
551
genus

species
215689
3
node114.members.0.js

species
338565
1
node115.members.0.js

7
genus
53335
1
node116.members.0.js

node117.members.0.js
1
470934
species

node118.members.0.js
species
553
1

species
1891675
1
node119.members.0.js

node120.members.0.js
1484157
species
3

135622
order
22

22
family
267888

22
genus
53246
node123.members.0.js
5

node124.members.0.js
283699
species
2

node125.members.0.js
2
species
28109

node126.members.0.js
species
247523
2

1720343
species
2
node127.members.0.js

1
species
228
node128.members.0.js

2
28107
species
node129.members.0.js

43659
species
1
node130.members.0.js

node131.members.0.js
species
394751
2

node132.members.0.js
152297
species
1

node133.members.0.js
1
176102
species

1
species
43662
node134.members.0.js

5
135623
order

family
641
5

genus
657
2

species
38293
2
node138.members.0.js

662
genus
3

node140.members.0.js
1
212663
species

2
species group
717610

node142.members.0.js
species
670
1

663
species
1
node143.members.0.js

7
72274
order

family
135621
7

7
genus
286

136843
species group
1

species
76761
1
node148.members.0.js

node149.members.0.js
198620
species
1

930166
species
1
node150.members.0.js

1
136841
species group

species subgroup
1232139
1

1
1149133
species
node153.members.0.js

node154.members.0.js
1
species
157783

node155.members.0.js
species
1283291
1

species group
136849
1

species subgroup
251695
1

317
species
1
node158.members.0.js

395
135614
order

family
32033
395

genus
40323
1

995085
species group
1

node163.members.0.js
40324
species
1

394
genus
338

node165.members.0.js
393
339
species

1
species
56454
node166.members.0.js

201
class
28216

150
80840
order
node168.members.0.js
1

119060
family
4

1
genus
106589

node171.members.0.js
248026
species
1

1
32008
genus

node173.members.0.js
1
species
337

48736
genus
2

node175.members.0.js
2
species
190721

1
family
75682

1
genus
202907

1
279058
species
node178.members.0.js

80864
family
1

1
12916
genus

1
species
80869
node181.members.0.js

family
506
143

222
genus
143

143
species
85698
node184.members.0.js

206351
order
51

51
family
1499392

50
90153
no rank

535
genus
50
node188.members.0.js
1

49
536
species
node189.members.0.js

1
568394
genus

node191.members.0.js
1
species
748280

1783272
no rank
98886

phylum
201174
62103

62103
class
1760
13
node194.members.0.js

85010
order
2

2070
family
2

1
1835
genus

node198.members.0.js
1
1836
species

1
674734
genus

species
1653480
1
node200.members.0.js

order
85011
2

2
family
2062

2
genus
1883

1852274
species group
1

node205.members.0.js
species
1888
1

node206.members.0.js
1
species
862751

85007
order
61694

1653
family
61694

1716
genus
61694
1673
node209.members.0.js

node210.members.0.js
161879
species
1

node211.members.0.js
species
161896
1

1718
species
59637
node212.members.0.js

node213.members.0.js
1
species
191493

node214.members.0.js
1
1230998
species

108486
species
1
node215.members.0.js

node216.members.0.js
species
1408191
4

node217.members.0.js
1
species
1705

1
1223514
species
node218.members.0.js

node219.members.0.js
11
species
1652495

node220.members.0.js
species
92706
336

28028
species
1
node221.members.0.js

1721
species
23
node222.members.0.js

152794
species
1
node223.members.0.js

191610
species
1
node224.members.0.js

order
85006
387

1
family
85023

1
no rank
1655488

1655489
no rank
1

1
529883
genus

node230.members.0.js
species
529884
1

384
1268
family

1
genus
1663

species
2020486
1
node233.members.0.js

383
genus
1269

1270
species
383
node235.members.0.js

85019
family
1

1
1696
genus

1
1703
species
node238.members.0.js

1
85022
family

1
43673
genus

1
43674
species
node241.members.0.js

2
85012
order

family
83676
2

2
2013
genus

node245.members.0.js
2
species
280236

85009
order
2

31957
family
2

node248.members.0.js
1
genus
1912216
2

1
1747
species
node249.members.0.js

order
85004
1

31953
family
1

genus
1678
1

node253.members.0.js
1
1689
species

1
phylum
544448

1
class
31969

186328
order
1

2131
family
1

node258.members.0.js
1
genus
2132

1
no rank
1798711

1
1117
phylum

order
1161
1

1162
family
1

1
1177
genus

1
species
1869241
node264.members.0.js

1297
phylum
1

class
188787
1

1
order
68933

1
family
188786

genus
65551
1

node270.members.0.js
species
52022
1

36780
1239
phylum
2
node271.members.0.js

class
909932
1

order
909929
1

1
1843490
family

1
genus
365348

node276.members.0.js
1
484770
species

91061
class
36766
node277.members.0.js
3

186826
order
4

186827
family
1

1
1375
genus

1376
species
1
node281.members.0.js

33958
family
3

3
genus
1578

node284.members.0.js
1
1074467
species

node285.members.0.js
1
species
1584

1
species
1138822
node286.members.0.js

4
node287.members.0.js
1385
order
36759

15
family
186818

13
genus
1372

node290.members.0.js
2058136
species
11

node291.members.0.js
1
species
200991

node292.members.0.js
1374
species
1

genus
648802
2

node294.members.0.js
species
241244
2

4
90964
family

1
node296.members.0.js
1279
genus
4

node297.members.0.js
1
species
1715860

1
species
308354
node298.members.0.js

node299.members.0.js
1
species
29385

1
186820
family

1
1637
genus

1
1639
species
node302.members.0.js

family
186817
36718

400634
genus
2

1421
species
1
node305.members.0.js

node306.members.0.js
1
28031
species

genus
1906945
1

1426
species
1
node308.members.0.js

genus
1386
36714
node309.members.0.js
16534

86661
species group
16

1839798
species
1
node311.members.0.js

species
1392
2
node312.members.0.js

node313.members.0.js
13
1396
species

1
species
666686
node314.members.0.js

node315.members.0.js
species
1837130
5

2026248
species
222
node316.members.0.js

node317.members.0.js
8
2049935
species

node318.members.0.js
1
species
1570330

species group
653685
19662

node320.members.0.js
species
1452
1

1938374
species subgroup
18

1390
species
5
node322.members.0.js

species
492670
13
node323.members.0.js

119858
species
19
node324.members.0.js

1402
species
19094
node325.members.0.js

522
1648923
species
node326.members.0.js

node327.members.0.js
1423
species
8

species
859143
1
node328.members.0.js

node329.members.0.js
species
1856406
234

node330.members.0.js
species
1664069
20

species
1404
2
node331.members.0.js

1
1408
species
node332.members.0.js

33932
species
1
node333.members.0.js

node334.members.0.js
species
1409
6

1055323
genus
1

node336.members.0.js
33936
species
1

1
family
186823

432330
genus
1

node339.members.0.js
1
1903704
species

16
family
186822

44249
genus
16

species
1536770
1
node342.members.0.js

node343.members.0.js
1
61624
species

node344.members.0.js
species
162209
1

node345.members.0.js
12
189426
species

1
species
1406
node346.members.0.js

class
186801
11

1
order
68295

1
543371
family

1
genus
28895

node351.members.0.js
species
29329
1

10
order
186802

7
31979
family

6
genus
1485
node354.members.0.js
1

node355.members.0.js
1
1497
species

1
1488
species
node356.members.0.js

1
species
1513
node357.members.0.js

node358.members.0.js
species
1520
2

1
49082
genus
node359.members.0.js

1
family
186804

genus
1481960
1

1731
species
1
node362.members.0.js

family
186803
1

genus
698776
1

1
29360
species
node365.members.0.js

node366.members.0.js
1
186807
family

1
57723
phylum

1
class
1562566

1
458032
genus

node370.members.0.js
1
species
458033

phylum
203691
1

class
203692
1

1
order
136

1
137
family

genus
157
1

species
81028
1
node376.members.0.js

1783270
no rank
7

68336
no rank
7

7
976
phylum

117747
class
1

1
order
200666

84566
family
1

1
84568
no rank

1
species
1986952
node384.members.0.js

3
117743
class

200644
order
3

node387.members.0.js
1
3
49546
family

genus
326319
1

node389.members.0.js
species
983548
1

1
112040
genus

1
species
63186
node391.members.0.js

class
200643
2

171549
order
2

family
815
1

genus
816
1

node396.members.0.js
1
376805
species

1
171552
family

genus
838
1

28131
species
1
node399.members.0.js

1
class
768503

768507
order
1

1853232
family
1

1
genus
323449

1
388950
species
node404.members.0.js

superkingdom
2157
4

4
phylum
28890

no rank
2283794
2

183925
class
2

2
2158
order

family
2159
2

2172
genus
2

230361
species
1
node412.members.0.js

species
83816
1
node413.members.0.js

no rank
2290931
1

1
class
224756

1
order
2191

family
2194
1

45989
genus
1

node419.members.0.js
2198
species
1

class
183980
1

2231
order
1

1
2232
family

genus
2233
1

node424.members.0.js
1
species
1316941
