# Supplementary material for: Benchmarking the MinION: Evaluating long reads for microbial profiling
Source: Sci Rep. 2020 Mar 20;10:5125. doi: 10.1038/s41598-020-61989-x (PMC7083898; doi:10.1038/s41598-020-61989-x)
Supplement: Supplementary file 2 — Supplementary information2. [file 41598_2020_61989_MOESM2_ESM.zip › sample_barcode_1/kraken.html]

Javascript must be enabled to view this page.

members
magnitude
magnitudeUnassigned
count
unassigned
taxon
rank

BC1\_kraken\_krona

node0.members.0.js
14
142008

15
node1.members.0.js
superkingdom
139260
2

1224
41940
phylum
node2.members.0.js
7

28216
class
184

136
80840
order

506
family
136

4
genus
node6.members.0.js
136
222

109
node7.members.0.js
species
132
85698

no rank
762376
node8.members.0.js
1

562971
no rank
node9.members.0.js
10

node10.members.0.js
1167634
no rank
12

48
order
206351

family
1499392
48

48
90153
no rank

1
genus
node14.members.0.js
48
535

47
536
species

243365
no rank
node16.members.0.js
47

41749
1236
10
class
node17.members.0.js

4
order
135622

family
267888
4

53246
4
node20.members.0.js
genus
3

1
node21.members.0.js
247523
species

91347
41347
order
node22.members.0.js
106

1903411
10533
family
node23.members.0.js
1

613
10532
node24.members.0.js
genus
11

10520
node25.members.0.js
47917
species

species
82996
1

no rank
1006598
node27.members.0.js
1

5
family
1903410

5
204037
1
genus
node29.members.0.js

4
node30.members.0.js
1089444
species

family
1903412
2

genus
635
2

67780
species
node33.members.0.js
2

family
1903409
1

551
genus
node35.members.0.js
1

543
30700
family
node36.members.0.js
350

620
4
node37.members.0.js
genus
1

3
species
622

no rank
300267
node39.members.0.js
3

genus
node40.members.0.js
7
570
39

7
node41.members.0.js
571
species

1
species
1134687
node42.members.0.js

573
14
species
node43.members.0.js
7

1365186
no rank
node44.members.0.js
2

72407
5
node45.members.0.js
subspecies
4

1328324
no rank
node46.members.0.js
1

8
node47.members.0.js
species
1905288

node48.members.0.js
species
1463165
2

648
node49.members.0.js
genus
26375
413496

node50.members.0.js
species
8
413503
14

6
node51.members.0.js
no rank
1159491

28141
25681
species
node52.members.0.js
23366

node53.members.0.js
1138308
no rank
1072

node54.members.0.js
956149
no rank
98

node55.members.0.js
no rank
290339
1145

413502
species
19

node57.members.0.js
no rank
693216
19

7
535744
species

1074000
no rank
node59.members.0.js
7

1
species
413497

subspecies
413498
1

no rank
1159554
node62.members.0.js
1

5
species
413501

5
node64.members.0.js
no rank
1159613

genus
544
9

species
545
node66.members.0.js
1

1
35703
species

node68.members.0.js
1261127
no rank
1

5
species group
1344959

species
node70.members.0.js
4
546
5

node71.members.0.js
1333848
no rank
1

species
67824
node72.members.0.js
2

genus
1330546
6

6
1334193
species
node74.members.0.js

2
191675
no rank

1
no rank
84563

genus
1682492
1

1
node78.members.0.js
1410383
species

1
36866
no rank

693444
species
node80.members.0.js
1

561
genus
44

1
208962
species
node82.members.0.js

562
43
node83.members.0.js
species
30

3
no rank
585397
node84.members.0.js

3
1038927
no rank

1134782
no rank
node86.members.0.js
1

no rank
1048254
node87.members.0.js
2

no rank
1358422
node88.members.0.js
2

node89.members.0.js
758831
no rank
1

node90.members.0.js
585057
no rank
3

1
node91.members.0.js
no rank
1050617

1
genus
158483

species
158822
node93.members.0.js
1

genus
929812
1

1
species
929813
node95.members.0.js

5
genus
590

28901
5
species
node97.members.0.js
2

3
59201
1
node98.members.0.js
subspecies

1
no rank
90371
node99.members.0.js

1
node100.members.0.js
no rank
600

547
3856
node101.members.0.js
genus
11

1952
species group
node102.members.0.js
3844
354276

208224
species
node103.members.0.js
5

node104.members.0.js
species
1812935
6

node105.members.0.js
species
77
158836
1566

70
node106.members.0.js
subspecies
301102

301105
subspecies
node107.members.0.js
113

14
1296536
subspecies
node108.members.0.js

1273
299766
subspecies
node109.members.0.js

node110.members.0.js
1812934
subspecies
19

5
299767
species
node111.members.0.js

7
1915310
species
node112.members.0.js

node113.members.0.js
species
232
550
265

subspecies
336306
8

no rank
716541
node115.members.0.js
6

no rank
1211025
node116.members.0.js
2

5
1045856
no rank
node117.members.0.js

node118.members.0.js
1354030
no rank
5

15
69219
subspecies

15
no rank
1104326
node120.members.0.js

13
species
node121.members.0.js
38
61645

640513
no rank
node122.members.0.js
19

1421338
no rank
node123.members.0.js
6

1
node124.members.0.js
399742
species

8
160674
genus

node126.members.0.js
species
575
7

1
node127.members.0.js
species
54291

1
135619
order

1
family
28256

1
genus
2745

node131.members.0.js
1178482
species
1

order
135614
386

386
family
32033

386
338
4
genus
node134.members.0.js

382
339
27
species
node135.members.0.js

no rank
359385
1

node137.members.0.js
990315
no rank
1

340
354
node138.members.0.js
no rank
313

node139.members.0.js
no rank
314565
1

node140.members.0.js
190485
no rank
40

1
135623
order

1
family
641

1
662
genus

1
species group
717610

1
node145.members.0.js
species
663

no rank
node146.members.0.js
3
1783272
97305

node147.members.0.js
phylum
3
1239
35950

class
186801
1

order
186802
1

1
family
186807

1
genus
1562

59610
species
1

node153.members.0.js
no rank
349161
1

class
node154.members.0.js
2
91061
35946

186826
order
1

1
81852
family

1350
genus
1

node158.members.0.js
species
1352
1

order
node159.members.0.js
9
1385
35943

90964
family
1

genus
1279
1

1
species
29385

147452
subspecies
1

node164.members.0.js
no rank
342451
1

8
node165.members.0.js
family
35920
186817

15829
genus
node166.members.0.js
35912
1386

1773
node167.members.0.js
species group
19722
653685

146
119858
species
node168.members.0.js

1550
1648923
1468
node169.members.0.js
species

node170.members.0.js
no rank
766760
82

species
node171.members.0.js
32
1423
50

node172.members.0.js
936156
no rank
4

13
135461
6
node173.members.0.js
subspecies

535026
no rank
node174.members.0.js
3

node175.members.0.js
1404258
no rank
3

node176.members.0.js
1052588
no rank
1

1
96241
subspecies

node178.members.0.js
655816
no rank
1

10037
species
node179.members.0.js
16163
1402

26
node180.members.0.js
no rank
1126218

6100
node181.members.0.js
no rank
279010

37
1938374
8
node182.members.0.js
species subgroup

13
492670
7
species
node183.members.0.js

node184.members.0.js
1458206
no rank
5

1
node185.members.0.js
no rank
1338518

node186.members.0.js
species
5
1390
16

1
node187.members.0.js
no rank
692420

6
1292358
no rank
node188.members.0.js

1
node189.members.0.js
no rank
1412898

no rank
1034836
node190.members.0.js
3

1452
3
species
node191.members.0.js
2

1
node192.members.0.js
no rank
1239783

4
node193.members.0.js
561879
species

4
86664
species
node194.members.0.js

node195.members.0.js
species
1178537
1

species
86665
2

2
node197.members.0.js
no rank
272558

1581038
species
node198.members.0.js
1

node199.members.0.js
species
1664069
95

1
1479
species
node200.members.0.js

species
756828
node201.members.0.js
9

5
1408
species
node202.members.0.js

species
1441095
node203.members.0.js
4

species
665099
1

1
node205.members.0.js
1196031
no rank

1
species
1478
node206.members.0.js

5
node207.members.0.js
1774743
species

4
species
1398
node208.members.0.js

27
node209.members.0.js
species group
180
86661

node210.members.0.js
species
1405
5

species
node211.members.0.js
75
1396
92

1
no rank
526968
node212.members.0.js

no rank
1217984
node213.members.0.js
2

4
no rank
451709
node214.members.0.js

2
node215.members.0.js
no rank
1454382

288681
no rank
node216.members.0.js
7

1
347495
no rank
node217.members.0.js

1
node218.members.0.js
1892404
species

node219.members.0.js
species
27
1428
36

1
node220.members.0.js
1195464
no rank

29339
6
no rank
node221.members.0.js
3

no rank
570416
node222.members.0.js
2

1
no rank
1279365
node223.members.0.js

node224.members.0.js
529122
no rank
1

1
29337
no rank

no rank
930170
node226.members.0.js
1

1392
19
node227.members.0.js
species
13

no rank
1392837
node228.members.0.js
3

node229.members.0.js
1449979
no rank
3

1
node230.members.0.js
79883
species

1837130
species
node231.members.0.js
8

30
node232.members.0.js
species
1856406

300825
species
1

1
node234.members.0.js
no rank
1246626

4
1471
species

4
796606
no rank
node236.members.0.js

12
186822
family

genus
44249
12

node239.members.0.js
189426
species
12

1
family
186818

1
genus
1372

1
node242.members.0.js
species
192421

3
phylum
node243.members.0.js
61352
201174

1760
61349
node244.members.0.js
class
14

1
85009
order

family
31957
1

1
genus
1912216

1
node248.members.0.js
1747
species

60957
order
85007

60957
family
1653

genus
node251.members.0.js
5039
1716
60957

species
349751
2

1224162
no rank
node253.members.0.js
2

6
species
1721

node255.members.0.js
no rank
1121353
6

1697
species
node256.members.0.js
1

3
node257.members.0.js
species
5
1719

2
no rank
1087451
node258.members.0.js

1718
55848
species
node259.members.0.js
53383

no rank
1232384
node260.members.0.js
1

no rank
1079988
node261.members.0.js
315

no rank
1310161
node262.members.0.js
72

node263.members.0.js
1232383
no rank
2

196627
752
no rank
node264.members.0.js
660

92
1204414
no rank
node265.members.0.js

node266.members.0.js
no rank
340322
1323

1
160386
species

1285583
no rank
node268.members.0.js
1

1
node269.members.0.js
species
43771

node270.members.0.js
species
38289
1

152794
species
2

node272.members.0.js
no rank
196164
2

9
species
92706

no rank
1232385
node274.members.0.js
9

2
1050174
species
node275.members.0.js

node276.members.0.js
species
1705
2

1
species
38305

1
1224164
no rank
node278.members.0.js

1231000
species
1

1
no rank
1408189
node280.members.0.js

1652495
species
node281.members.0.js
17

species
156976
node282.members.0.js
1

1408191
species
14

931089
no rank
node284.members.0.js
14

1
1717
species
node285.members.0.js

species
42817
1

1348662
no rank
node287.members.0.js
1

2
108486
species

node289.members.0.js
no rank
1451189
2

376
order
85006

family
1268
376

1269
genus
376

376
1270
339
node293.members.0.js
species

37
no rank
465515
node294.members.0.js

85012
order
1

1
family
83676

genus
2013
1

1
280236
species

no rank
1235441
node299.members.0.js
1

node300.members.0.js
2734
