# Supplementary material for: Benchmarking the MinION: Evaluating long reads for microbial profiling
Source: Sci Rep. 2020 Mar 20;10:5125. doi: 10.1038/s41598-020-61989-x (PMC7083898; doi:10.1038/s41598-020-61989-x)
Supplement: Supplementary file 2 — Supplementary information2. [file 41598_2020_61989_MOESM2_ESM.zip › sample_barcode_1/kraken2.html]

Javascript must be enabled to view this page.

members
magnitude
magnitudeUnassigned
count
unassigned
taxon
rank

BC1\_kraken2\_krona

node0.members.0.js
142008
6

node1.members.0.js
1304

2157
3
superkingdom

phylum
3
28890

no rank
2290931
2

2
183963
class

1
1644060
order

family
1644061
1

genus
203193
1

species
229731
1

no rank
node10.members.0.js
1
358396

1
1644055
order

1
1963271
family

genus
56688
1

1
2247
species

node15.members.0.js
1
no rank
416348

2283796
1
no rank

class
1
183967

no rank
1234666
1

1495144
species
1
node19.members.0.js

12
2
superkingdom
node20.members.0.js
140695

1783272
3
node21.members.0.js
98314
no rank

2
1239
phylum
36505
node22.members.0.js

class
1
1737404

order
1
1737405

family
1
1570339

genus
165779
1

1
33034
species

525919
no rank
node28.members.0.js
1

node29.members.0.js
36502
class
91061
1

186826
3
order

family
33958
3

genus
1253
1

114090
species
1
node33.members.0.js

1578
2
genus

240427
node35.members.0.js
1
species

node36.members.0.js
1
species
1007676

node37.members.0.js
36498
order
1385
7

186822
12
family

44249
12
genus

189426
12
node40.members.0.js
species

186817
1
36471
node41.members.0.js
family

genus
node42.members.0.js
36469
11745
1386

species
node43.members.0.js
348
1664069

421767
1
node44.members.0.js
species

node45.members.0.js
1
species
129985

node46.members.0.js
9
species
86664

1565991
species
3
node47.members.0.js

7
324767
species

1367477
7
node49.members.0.js
no rank

1467
species
node50.members.0.js
1

756828
2
node51.members.0.js
species

35841
species
3
node52.members.0.js

6
node53.members.0.js
species
79880

species
node54.members.0.js
4
2093834

species
79885
3

3
node56.members.0.js
no rank
398511

98228
species
2
node57.members.0.js

species
node58.members.0.js
1
1705566

653685
1395
node59.members.0.js
23913
species group

1648923
3496
3665
node60.members.0.js
species

no rank
node61.members.0.js
169
766760

species
18187
node62.members.0.js
10844
1402

279010
7269
node63.members.0.js
no rank

node64.members.0.js
74
no rank
1126218

11
node65.members.0.js
species
1452
9

no rank
2
node66.members.0.js
1239783

species subgroup
172
node67.members.0.js
8
1938374

148
node68.members.0.js
species
492670
136

1458206
no rank
node69.members.0.js
10

1155777
no rank
node70.members.0.js
1

no rank
1
node71.members.0.js
1385727

12
1390
species
node72.members.0.js
16

no rank
node73.members.0.js
1
692420

node74.members.0.js
2
no rank
1292358

no rank
node75.members.0.js
1
1412898

species
node76.members.0.js
362
119858

121
node77.members.0.js
species
1423
72

1
node78.members.0.js
no rank
936156

subspecies
14
node79.members.0.js
483913

subspecies
2
node80.members.0.js
86029

subspecies
11
node81.members.0.js
2
96241

655816
no rank
7
node82.members.0.js

1052585
2
node83.members.0.js
no rank

9
135461
subspecies
node84.members.0.js
21

no rank
5
node85.members.0.js
1404258

1302650
node86.members.0.js
4
no rank

1192196
no rank
node87.members.0.js
1

1052588
node88.members.0.js
2
no rank

1178537
1
node89.members.0.js
species

species group
1792192
7

293387
4
node91.members.0.js
species

1049581
node92.members.0.js
3
species

2026248
species
node93.members.0.js
2

species
10
node94.members.0.js
2049935

node95.members.0.js
2
species
859143

79883
5
node96.members.0.js
species

1478
2
species

node98.members.0.js
2
no rank
1349754

1479
species
6
node99.members.0.js

2011012
species
node100.members.0.js
3

3
1404
species

2
node102.members.0.js
no rank
1452722

1348623
node103.members.0.js
1
no rank

node104.members.0.js
3
species
352858

species
node105.members.0.js
13
561879

species
node106.members.0.js
3
1547283

666686
species
2
node107.members.0.js

86661
27
node108.members.0.js
216
species group

580165
1
node109.members.0.js
species

species
123
node110.members.0.js
16
1396

no rank
98
node111.members.0.js
1003239

288681
no rank
node112.members.0.js
4

2
node113.members.0.js
no rank
1454382

node114.members.0.js
1
no rank
526991

222523
no rank
node115.members.0.js
1

no rank
node116.members.0.js
1
526984

32
1428
species
36
node117.members.0.js

1
29339
no rank

1261129
1
node119.members.0.js
no rank

no rank
1
node120.members.0.js
1442

node121.members.0.js
1
no rank
527019

1218175
no rank
1
node122.members.0.js

species
node123.members.0.js
5
4
64104

527000
no rank
node124.members.0.js
1

species
24
node125.members.0.js
14
1392

260799
2
node126.members.0.js
no rank

261591
3
node127.members.0.js
no rank

no rank
5
node128.members.0.js
1449979

1837130
species
node129.members.0.js
2

3
665099
species

1196031
3
node131.members.0.js
no rank

1398
species
7
node132.members.0.js

species
7
86665

272558
no rank
node134.members.0.js
7

1441095
species
node135.members.0.js
16

33932
node136.members.0.js
7
species

2
300825
species

2
node138.members.0.js
no rank
1246626

2009331
node139.members.0.js
2
species

2
node140.members.0.js
species
264697

node141.members.0.js
1
species
1127744

species
node142.members.0.js
2
632773

67
node143.members.0.js
species
1856406

node144.members.0.js
10
species
1408
9

315750
1
node145.members.0.js
no rank

1413
1
species

1
node147.members.0.js
no rank
649639

node148.members.0.js
2
species
199441

species
node149.members.0.js
3
1402861

species
5
1471

node151.members.0.js
5
no rank
796606

1581038
node152.members.0.js
3
species

genus
129337
1

1
node154.members.0.js
species
1422

186820
1
family

genus
1637
1

1
node157.members.0.js
species
1639

node158.members.0.js
5
family
186818
1

3
1372
genus

species
2
node160.members.0.js
2058136

1526927
node161.members.0.js
1
species

648800
1
genus

species
76853
1

1
node164.members.0.js
no rank
1002809

family
2
90964

genus
2
1279

species
1
node167.members.0.js
1280

node168.members.0.js
1
species
29385

3
201174
phylum
node169.members.0.js
61800

node170.members.0.js
61797
class
1760
17

85009
1
order

family
31957
1

genus
1912216
1

1747
species
node174.members.0.js
1

order
61391
85007

1762
1
family

1866885
1
genus

species
1
110539

350058
no rank
1
node179.members.0.js

family
85025
3

1827
3
genus

1990687
species
1
node182.members.0.js

1833
species
node183.members.0.js
1

103816
species
node184.members.0.js
1

family
61387
1653

genus
node186.members.0.js
61387
3428
1716

species
108486
3

1451189
no rank
3
node188.members.0.js

node189.members.0.js
1
species
35755

species
5
38288

node191.members.0.js
5
no rank
585529

species
node192.members.0.js
1
43770

1721
14
species

1121353
14
node194.members.0.js
no rank

species
349751
1

1
node196.members.0.js
no rank
1224162

species
160386
4

1285583
node198.members.0.js
4
no rank

species
node199.members.0.js
3
146827

1050174
4
node200.members.0.js
species

species
38305
1

1224164
no rank
1
node202.members.0.js

92706
11
species

1232385
node204.members.0.js
11
no rank

28028
species
node205.members.0.js
1

42817
2
species

node207.members.0.js
2
no rank
1348662

node208.members.0.js
3
species
38301

species
node209.members.0.js
1
191610

1724
node210.members.0.js
5
species

11
1719
species
12
node211.members.0.js

1074485
1
node212.members.0.js
no rank

2080740
species
node213.members.0.js
1

species
1408191
40

no rank
node215.members.0.js
40
931089

1652495
node216.members.0.js
55
species

1
node217.members.0.js
species
156976

species
3
node218.members.0.js
136857

203263
1
species

1431546
no rank
node220.members.0.js
1

species
1
node221.members.0.js
65058

53551
1718
species
57724
node222.members.0.js

340322
no rank
node223.members.0.js
2966

1310161
no rank
node224.members.0.js
7

no rank
14
node225.members.0.js
1232384

165
196627
no rank
353
node226.members.0.js

1204414
node227.members.0.js
188
no rank

no rank
node228.members.0.js
117
1232381

710
node229.members.0.js
no rank
1079988

1232383
node230.members.0.js
6
no rank

species
2
1223514

2
node232.members.0.js
no rank
1223515

38289
5
6
node233.members.0.js
species

306537
1
node234.members.0.js
no rank

161896
3
node235.members.0.js
species

187491
node236.members.0.js
1
species

species
1
node237.members.0.js
571915

node238.members.0.js
1
species
38302

1072256
2
node239.members.0.js
species

species
node240.members.0.js
1
1697

species
node241.members.0.js
7
5
1717

2
1806053
no rank

1450520
no rank
node243.members.0.js
2

1231000
2
species

2
node245.members.0.js
no rank
1408189

species
225326
7

no rank
7
node247.members.0.js
1121362

1404244
2
species

no rank
node249.members.0.js
2
1404245

2
node250.members.0.js
species
1705

169292
3
species

548476
no rank
3
node252.members.0.js

3
1230998
species

1437875
no rank
3
node254.members.0.js

species
152794
15

196164
15
node256.members.0.js
no rank

1121358
3
species

3
node258.members.0.js
no rank
558173

85011
2
order

2
2062
family

genus
1
1883

1852274
1
species group

species
1
node263.members.0.js
1888

2063
1
genus

2018025
node265.members.0.js
1
species

order
2
2039638

2
2162846
family

2039639
1
node268.members.0.js
genus

622681
1
genus

573600
species
1
node270.members.0.js

85008
1
order

family
1
28056

genus
1
1873

species
1
node274.members.0.js
2201999

order
1
85012

83676
1
family

2013
1
genus

280236
1
species

1235441
1
node279.members.0.js
no rank

382
85006
order

1268
379
family

genus
1269
379

1270
344
379
node283.members.0.js
species

465515
no rank
node284.members.0.js
35

1
85020
family

1
43668
genus

1
node287.members.0.js
species
1903186

family
1
85023

genus
33882
1

104336
species
1
node290.members.0.js

85021
1
family

53457
1
genus

857417
1
node293.members.0.js
species

6
1798711
no rank

phylum
6
1117

1161
2
order

family
1185
1

genus
1
373984

373994
node299.members.0.js
1
species

1
201821
no rank

no rank
1
1219117

1940762
node302.members.0.js
1
species

4
1890424
order

1890438
1
family

genus
1
47251

node306.members.0.js
1
species
111781

family
1
1890431

genus
1
217161

1
1173032
species

1
node310.members.0.js
no rank
1173020

1890426
2
family

genus
1129
1

species
1
node313.members.0.js
316278

genus
13034
1

species
1
292566

13035
node316.members.0.js
1
no rank

1783270
7
no rank

68336
7
no rank

1134404
1
phylum

class
1
795747

795748
1
order

family
1
1334117

1134403
1
genus

1
1134405
species

1191523
no rank
1
node325.members.0.js

976
6
phylum

class
1
768503

order
1
768507

1
1853232
family

89966
1
genus

1411621
species
1
node331.members.0.js

1
1937959
class

1
1936988
order

family
1
1937961

2349
1
genus

species
1
2350

760192
1
node337.members.0.js
no rank

117743
1
class

200644
1
order

family
1
49546

1016
1
genus

1848904
species
1
node342.members.0.js

117747
1
class

order
200666
1

family
1
84566

genus
1
423349

1300914
node347.members.0.js
1
species

class
2
200643

2
171549
order

family
1
171550

genus
239759
1

species
328814
1

no rank
node353.members.0.js
1
717959

1
815
family

genus
816
1

species
1
357276

997877
node357.members.0.js
1
no rank

node358.members.0.js
42360
phylum
1224
9

3
68525
subphylum

class
29547
1

213849
1
order

72294
1
family

1
2321108
no rank

1
28196
genus

663364
species
1
node365.members.0.js

28221
2
class

order
213118
1

1
213121
family

1
53318
genus

1
65555
species

1
node371.members.0.js
no rank
1167006

29
1
order

suborder
1
80811

1
39
family

genus
1
44

species
1
83453

node377.members.0.js
1
no rank
1294270

11
1236
class
42136
node378.members.0.js

order
4
135619

family
1
135620

1
28253
genus

400668
node382.members.0.js
1
species

28256
3
family

2745
1
node384.members.0.js
2
genus

1178482
species
1
node385.members.0.js

1
114403
no rank

114399
1
no rank

235572
1
genus

91844
species
node389.members.0.js
1

135622
16
order

family
267890
1

genus
1
22

species
1
359303

no rank
1
node394.members.0.js
323850

1
72275
family

1
2742
genus

species
node397.members.0.js
1
1749259

14
267888
family

53246
7
node399.members.0.js
14
genus

1
176102
species

no rank
1
node401.members.0.js
1312369

1
227
species

1314868
1
node403.members.0.js
no rank

1
node404.members.0.js
species
621376

species
node405.members.0.js
1
267375

species
1
228

326442
no rank
node407.members.0.js
1

247523
node408.members.0.js
1
species

species
1
node409.members.0.js
1514074

72274
4
order

4
135621
family

genus
4
286

node413.members.0.js
1
species
2201356

node414.members.0.js
1
species
2054915

node415.members.0.js
1
species
253237

1
node416.members.0.js
species
163011

118884
2
no rank

1
1273155
genus

585455
node419.members.0.js
1
species

1
33811
no rank

node421.members.0.js
1
species
1248727

91347
107
41698
node422.members.0.js
order

family
1
1903414

1
581
genus

1
node425.members.0.js
species
582

2
1903412
family

genus
635
2

1
node428.members.0.js
species
67780

1
1821960
species

667120
no rank
1
node430.members.0.js

451511
1
no rank

genus
1
447792

species
node433.members.0.js
1
1756993

1
1903411
family
node434.members.0.js
10597

1
1927833
genus

species
1
node436.members.0.js
1878942

genus
629
4

3
1649845
species group

632
2
3
node439.members.0.js
species

1
node440.members.0.js
no rank
1345702

species
630
1

subspecies
1
150053

no rank
1
node443.members.0.js
930944

7
613
genus
10591
node444.members.0.js

47917
node445.members.0.js
10579
species

node446.members.0.js
2
species
614
1

1346614
node447.members.0.js
1
no rank

82996
species
1
node448.members.0.js

node449.members.0.js
2
species
615
1

1401254
no rank
1
node450.members.0.js

543
211
30982
node451.members.0.js
family

no rank
3
191675

no rank
36866
3

1920109
species
3
node454.members.0.js

1
929812
genus

node456.members.0.js
1
species
929813

genus
1
2172100

2172103
species
1
node458.members.0.js

genus
14
node459.members.0.js
2
544

1344959
1
10
node460.members.0.js
species group

67827
node461.members.0.js
1
species

2077147
species
1
node462.members.0.js

2077149
species
1
node463.members.0.js

546
4
node464.members.0.js
5
species

no rank
1
node465.members.0.js
1333848

2066049
species
1
node466.members.0.js

species
node467.members.0.js
2
1
35703

1261127
node468.members.0.js
1
no rank

genus
620
2

species
622
1

300267
node471.members.0.js
1
no rank

species
623
1

1
42897
no rank

198214
1
node474.members.0.js
no rank

node475.members.0.js
83
genus
570
5

species
3
node476.members.0.js
548

species
node477.members.0.js
11
1905288

573
32
49
node478.members.0.js
species

no rank
node479.members.0.js
1
1365186

39831
2
subspecies

no rank
2
node481.members.0.js
861365

12
72407
subspecies
14
node482.members.0.js

1225181
no rank
1
node483.members.0.js

no rank
1
node484.members.0.js
1328324

571
13
node485.members.0.js
species

2026240
species
node486.members.0.js
2

26640
node487.members.0.js
genus
413496
521

species
26037
node488.members.0.js
23147
28141

956149
191
node489.members.0.js
no rank

1138308
no rank
node490.members.0.js
2562

290339
no rank
node491.members.0.js
137

species
5
413497

subspecies
413498
5

1159554
no rank
5
node494.members.0.js

species
1163710
5

node496.members.0.js
5
no rank
1073999

413503
12
18
node497.members.0.js
species

1159491
no rank
6
node498.members.0.js

species
9
413501

9
node500.members.0.js
no rank
1159613

33
413502
species

693216
no rank
33
node502.members.0.js

species
535744
12

no rank
12
node504.members.0.js
1074000

82
561
genus

1
208962
species
2
node506.members.0.js

1
node507.members.0.js
no rank
1440052

562
49
node508.members.0.js
80
species

2048781
no rank
2
node509.members.0.js

no rank
1
node510.members.0.js
1358422

1050617
no rank
node511.members.0.js
6

no rank
node512.members.0.js
3
585057

2
83334
no rank

no rank
1
node514.members.0.js
1328859

155864
node515.members.0.js
1
no rank

no rank
1
node516.members.0.js
1446746

861906
1
no rank

no rank
1
node518.members.0.js
216592

83333
node519.members.0.js
1
no rank

1
node520.members.0.js
no rank
930406

4
node521.members.0.js
no rank
405955

199310
no rank
node522.members.0.js
3

no rank
5
1038927

1133853
no rank
node524.members.0.js
1

no rank
2
node525.members.0.js
1134782

1048254
node526.members.0.js
2
no rank

585397
no rank
node527.members.0.js
1

node528.members.0.js
12
genus
160674
1

species
2
node529.members.0.js
54291

575
species
9
node530.members.0.js

83654
3
genus

species
node532.members.0.js
2
1920116

species
1
node533.members.0.js
1920114

1330545
2
genus

1
node535.members.0.js
species
1907578

species
1
node536.members.0.js
69220

genus
node537.members.0.js
3905
58
547

399742
node538.members.0.js
2
species

1827481
node539.members.0.js
1
species

1914861
species
node540.members.0.js
1

3842
node541.members.0.js
species group
354276
1161

node542.members.0.js
59
species
69218

1812935
12
node543.members.0.js
species

node544.members.0.js
7
species
299767

2077136
node545.members.0.js
2
species

node546.members.0.js
207
species
2027919

species
1778
node547.members.0.js
141
158836

subspecies
node548.members.0.js
51
1296536

subspecies
node549.members.0.js
217
301105

3
node550.members.0.js
subspecies
301102

1318
node551.members.0.js
subspecies
299766

subspecies
node552.members.0.js
48
1812934

species
node553.members.0.js
3
208224

1915310
9
node554.members.0.js
species

species
79
node555.members.0.js
23
61645

no rank
node556.members.0.js
47
640513

node557.members.0.js
9
no rank
1421338

448
550
species
522
node558.members.0.js

node559.members.0.js
9
no rank
1354030

node560.members.0.js
4
no rank
1045856

336306
1
node561.members.0.js
21
subspecies

no rank
node562.members.0.js
10
1211025

no rank
node563.members.0.js
10
716541

69219
40
subspecies

1104326
no rank
40
node565.members.0.js

2077137
species
node566.members.0.js
3

881260
node567.members.0.js
1
species

genus
590
16

28901
9
16
node569.members.0.js
species

1
59201
subspecies
node570.members.0.js
7

149539
1
node571.members.0.js
no rank

no rank
1
440524

no rank
1
node573.members.0.js
866913

28150
no rank
1
node574.members.0.js

3
node575.members.0.js
no rank
108619

1335483
2
genus

2
563
species

630626
node578.members.0.js
2
no rank

node579.members.0.js
5
genus
1330547
1

208223
species
4
node580.members.0.js

1903410
6
family

5
204037
genus

1089444
species
5
node583.members.0.js

1
122277
genus

species
1
55208

no rank
1
node586.members.0.js
1175631

family
2
1903409

genus
551
1

1922217
species
1
node589.members.0.js

genus
53335
1

553
node591.members.0.js
1
species

135623
3
order

641
1
node593.members.0.js
3
family

662
2
genus

76258
node595.members.0.js
2
species

135614
398
order

family
1
1775411

genus
2233801
1

2021234
1
node599.members.0.js
species

32033
397
family

1
40323
genus

species group
1
995085

species
1
40324

node604.members.0.js
1
no rank
1190567

genus
396
node605.members.0.js
3
338

1
643453
species group

species
346
1

no rank
1
454595

no rank
node609.members.0.js
1
1437881

339
30
392
node610.members.0.js
species

no rank
3
359385

3
node612.members.0.js
no rank
990315

313
340
no rank
359
node613.members.0.js

no rank
1
node614.members.0.js
314565

190485
40
node615.members.0.js
no rank

1358017
no rank
node616.members.0.js
1

1358015
node617.members.0.js
3
no rank

1358009
no rank
1
node618.members.0.js

1
1553900
class

order
213481
1

family
1
213483

genus
1
958

species
959
1

765869
no rank
node624.members.0.js
1

class
28211
10

204455
1
order

family
1
31989

1
263377
genus

1229727
species
1
node629.members.0.js

order
204457
3

2
41297
family

genus
165695
1

species
46429
1

no rank
1
node634.members.0.js
690566

1
1434046
genus

species
node636.members.0.js
1
1922222

1
335929
family

361177
1
genus

2060312
species
1
node639.members.0.js

1
204441
order

1
41295
family

genus
1
1263978

1
1263979
species

no rank
node644.members.0.js
1
1401328

356
5
order

255475
1
family

1
293088
genus

species
node648.members.0.js
1
1486262

family
82115
3

1
323620
genus

879274
node651.members.0.js
1
species

1
node652.members.0.js
no rank
227290

no rank
1
227292

28105
1
genus

1
node655.members.0.js
species
1842534

family
1
119045

1
407
genus

2202825
species
node658.members.0.js
1

class
28216
201

206351
51
order

family
1499392
51

no rank
90153
51

3
535
genus
51
node663.members.0.js

1108595
species
node664.members.0.js
1

536
5
47
node665.members.0.js
species

node666.members.0.js
42
no rank
243365

32003
1
order

1
90627
family

1443590
1
genus

species
node670.members.0.js
1
1188319

node671.members.0.js
149
order
80840
2

family
141
506

genus
517
1

1
node674.members.0.js
species
1697043

genus
140
node675.members.0.js
3
222

85698
118
node676.members.0.js
137
species

no rank
8
node677.members.0.js
562971

1167634
7
node678.members.0.js
no rank

762376
4
node679.members.0.js
no rank

75682
1
family

202907
1
genus

1
node682.members.0.js
species
279058

4
119060
family

1
48736
genus

305
species
1
node685.members.0.js

1
32008
genus

node687.members.0.js
1
species
416344

106589
2
genus

2
node689.members.0.js
species
164546

80864
1
family

1
283
genus

species
285
1

node693.members.0.js
1
no rank
1191062

1783257
1
no rank

74201
1
phylum

class
414999
1

415000
1
order

family
1
134623

278955
1
no rank

species
node700.members.0.js
1
794903

phylum
203691
1

1
203692
class

order
136
1

family
137
1

157
1
genus

species
167
1

no rank
node707.members.0.js
1
869209
