# Supplementary material for: Benchmarking the MinION: Evaluating long reads for microbial profiling
Source: Sci Rep. 2020 Mar 20;10:5125. doi: 10.1038/s41598-020-61989-x (PMC7083898; doi:10.1038/s41598-020-61989-x)
Supplement: Supplementary file 2 — Supplementary information2. [file 41598_2020_61989_MOESM2_ESM.zip › sample_barcode_2/centrifuge.html]

Javascript must be enabled to view this page.

members
magnitude
magnitudeUnassigned
count
unassigned
taxon
rank

BC2\_k1\_centrifuge\_results

node0.members.0.js
261834
11

superkingdom
2157
3

28890
3
phylum

no rank
2283794
1

183939
1
class

order
2182
1

2183
1
family

genus
2184
1

1
42879
species
node8.members.0.js

2290931
2
no rank

class
183963
2

order
1
1644060

1
1644061
family

genus
29287
1

species
29288
1
node14.members.0.js

order
1
1644055

1
2116545
no rank

node17.members.0.js
species
756883
1

node18.members.0.js
1176

node19.members.0.js
260644
16
2
superkingdom

phylum
1
32066

1
203490
class

203491
1
order

1129771
1
family

genus
1
168808

species
187101
1
node25.members.0.js

no rank
4
1783257

2
74201
phylum

class
2
203494

order
2
48461

family
1647988
2

genus
239934
2

node32.members.0.js
239935
2
species

phylum
203682
2

2
203683
class

order
112
2

family
2
126

genus
2
1676125

1331910
2
species
node38.members.0.js

phylum
1
200918

class
188708
1

order
1
2419

family
1643950
1

2420
1
genus

node44.members.0.js
1
2421
species

1783270
10
no rank

no rank
68336
10

9
976
phylum

class
117747
1

1
200666
order

1
84566
family

84567
1
genus

node52.members.0.js
species
1
188932

class
117743
3

order
200644
3

2
49546
family

1
237
genus

node57.members.0.js
1
55197
species

1
143222
genus

species
1
1729720
node59.members.0.js

no rank
313602
1

genus
1
1940138

node62.members.0.js
species
1415657
1

4
768503
class

order
768507
4

family
1853232
2

1
1379908
genus

node67.members.0.js
512763
1
species

genus
1
89966

node69.members.0.js
species
1484116
1

family
89373
2

107
1
genus

1
564064
species
node72.members.0.js

120831
1
genus

species
94254
1
node74.members.0.js

class
1
1853228

1
1853229
order

family
1
563835

genus
1
79328

node79.members.0.js
species
2029983
1

phylum
1
1090

1
191410
class

1
191411
order

1
191412
family

genus
1
256319

species
274537
1
node85.members.0.js

phylum
114762
17
1224
node86.members.0.js

subphylum
68525
3

3
28221
class

1
29
order

1
80811
suborder

31
1
family

32
1
genus

83455
1
species
node93.members.0.js

1
213462
order

1
213468
family

1
2357
genus

node97.members.0.js
2358
1
species

order
1
69541

family
1
213422

28231
1
genus

species
1
351604
node101.members.0.js

node102.members.0.js
28211
2
35
class

order
15
356

family
1
31993

133
1
genus

node106.members.0.js
655015
1
species

69277
1
family

genus
1
68287

species
1
593909
node109.members.0.js

2
45401
family

genus
1
59282

node112.members.0.js
1079
1
species

1
46913
genus

node114.members.0.js
species
1736675
1

41294
3
family

node116.members.0.js
genus
374
1
2

species
1
375
node117.members.0.js

genus
1073
1

1076
1
species
node119.members.0.js

1
118882
family

528
1
genus

node122.members.0.js
529
1
species

family
119045
1

genus
407
1

1
1479019
species
node125.members.0.js

family
82115
5

genus
323620
1

879274
1
species
node128.members.0.js

227290
3
no rank

genus
1
357

node131.members.0.js
359
1
species

genus
2
379

species
1
384
node133.members.0.js

node134.members.0.js
species
1
56730

no rank
227292
1

28105
1
genus

no rank
1
663276

species
1
380
node138.members.0.js

45404
1
family

genus
120652
1

199596
1
species
node141.members.0.js

order
3
204457

3
41297
family

165697
1
genus

node145.members.0.js
33050
1
species

genus
1
165695

node147.members.0.js
1855519
1
species

13687
1
genus

node149.members.0.js
1
1560345
species

1
204458
order

1
76892
family

genus
75
1

69395
1
species
node153.members.0.js

204441
3
order

2
433
family

genus
153497
2

node157.members.0.js
species
153496
2

41295
1
family

191
1
genus

node160.members.0.js
192
1
species

766
1
order

1
942
family

genus
768
1

node164.members.0.js
769
1
species

204455
10
order

node166.members.0.js
family
10
1
31989

genus
265
1

species
1
34003
node168.members.0.js

1
299261
genus

1
299262
species
node170.members.0.js

2
1579315
genus

species
2
1579316
node172.members.0.js

genus
53945
1

node174.members.0.js
1217908
1
species

node175.members.0.js
302485
3
2
genus

1
221822
species
node176.members.0.js

2211641
1
genus

species
245188
1
node178.members.0.js

class
1236
18
114444
node179.members.0.js

order
72274
22

family
1
468

1
469
genus

node183.members.0.js
1
1646498
species

family
21
135621

genus
1
1849530

node186.members.0.js
species
1
1697053

node187.members.0.js
2
20
286
genus

node188.members.0.js
101564
1
species

species
1
1856685
node189.members.0.js

136841
5
species group

node191.members.0.js
species
5
287

node192.members.0.js
species
1853130
2

136842
1
species group

species
1
296
node194.members.0.js

species
2
1283291
node195.members.0.js

species group
1
136843

node197.members.0.js
species
294
1

species group
136846
4

species subgroup
4
578833

node200.members.0.js
316
4
species

1
1931241
species
node201.members.0.js

order
135625
3

712
1
3
family
node203.members.0.js

214906
1
genus

node205.members.0.js
species
1
731

1
724
genus

node207.members.0.js
1
727
species

562
135614
order

32033
562
1
family
node209.members.0.js

genus
40323
2

1
995085
species group

species
1
40324
node212.members.0.js

node213.members.0.js
species
1793721
1

557
338
genus

species group
1
643453

node216.members.0.js
species
346
1

node217.members.0.js
species
339
555

1
56460
species
node218.members.0.js

genus
68
1

1
435897
species
node220.members.0.js

genus
83618
1

species
1
415229
node222.members.0.js

1
118884
no rank

no rank
33811
1

node225.members.0.js
1
1248727
species

order
135624
2

2
84642
family

node228.members.0.js
genus
1
642

genus
1
43947

1
43948
species
node230.members.0.js

order
4
135622

1
72275
family

genus
1621534
1

species
326544
1
node234.members.0.js

267890
1
family

22
1
genus

node237.members.0.js
1
93973
species

family
267888
1

node239.members.0.js
53246
1
genus

family
267893
1

node241.members.0.js
1
135575
genus

order
91347
224
113810
node242.members.0.js

node243.members.0.js
543
99073
461
family

no rank
17
191675

36866
16
no rank

species
1920128
1
node246.members.0.js

node247.members.0.js
1
693444
species

2066051
11
species
node248.members.0.js

node249.members.0.js
species
3
891974

no rank
84563
1

84564
1
no rank

genus
1
203804

node253.members.0.js
101534
1
species

genus
409304
1

species
168169
1
node255.members.0.js

83654
11
1
genus
node256.members.0.js

node257.members.0.js
1920116
9
species

species
83655
1
node258.members.0.js

590
35
genus

node260.members.0.js
species
35
28901

570
18
117
genus
node261.members.0.js

species
3
1134687
node262.members.0.js

node263.members.0.js
species
244366
1

node264.members.0.js
5
2026240
species

species
53
573
node265.members.0.js

3
548
species
node266.members.0.js

node267.members.0.js
species
5
1463165

4
1905288
species
node268.members.0.js

species
571
25
node269.members.0.js

1
579
genus

node271.members.0.js
species
1
61648

genus
1
1048757

species
1048758
1
node273.members.0.js

547
274
5781
genus
node274.members.0.js

node275.members.0.js
species
2051905
43

5456
354276
species group

node277.members.0.js
299767
3
species

species
1915310
4
node278.members.0.js

158836
3746
3731
species
node279.members.0.js

4
1296536
subspecies
node280.members.0.js

subspecies
1812934
11
node281.members.0.js

1
69218
species
node282.members.0.js

1693
550
species
node283.members.0.js

species
1
1812935
node284.members.0.js

node285.members.0.js
species
2
2027919

3
61645
species
node286.members.0.js

node287.members.0.js
208224
3
species

node288.members.0.js
species
1692238
1

1166130
1
species
node289.members.0.js

species
1560339
2
node290.members.0.js

1914861
2
species
node291.members.0.js

2
399742
species
node292.members.0.js

3
929812
genus

node294.members.0.js
species
3
929813

1330545
2
genus

node296.members.0.js
species
61646
2

1
1330547
genus

1
1158459
species
node298.members.0.js

561
228
genus

species
562
228
node300.members.0.js

552
92364
413496
genus
node301.members.0.js

species
64
413502
node302.members.0.js

node303.members.0.js
413497
24
species

47
413501
species
node304.members.0.js

node305.members.0.js
22
1163710
species

413503
748
species
node306.members.0.js

species
90758
28141
node307.members.0.js

species
535744
149
node308.members.0.js

genus
158483
5

node310.members.0.js
species
5
158822

1
160674
genus

node312.members.0.js
575
1
species

1335483
1
genus

node314.members.0.js
species
563
1

16
620
genus

node316.members.0.js
species
622
14

node317.members.0.js
species
623
2

node318.members.0.js
544
2
21
genus

node319.members.0.js
35703
2
species

1344959
16
species group

species
1
67827
node321.members.0.js

species
11
546
node322.members.0.js

node323.members.0.js
2077149
2
species

2
57706
species
node324.members.0.js

species
1
67824
node325.members.0.js

genus
6
1330546

node327.members.0.js
species
1334193
2

species
61647
4
node328.members.0.js

family
19
1903412

635
19
genus

node331.members.0.js
67780
19
species

4
1903414
family

2
581
genus

582
2
species
node334.members.0.js

586
1
genus

species
126385
1
node336.members.0.js

genus
626
1

node338.members.0.js
1
628
species

family
1903410
13

genus
2
71655

node341.members.0.js
1109412
2
species

genus
2
84565

node343.members.0.js
species
1
1239307

1
63612
species
node344.members.0.js

204037
9
genus

1089444
9
species
node346.members.0.js

node347.members.0.js
family
1903411
14449
1

1745211
1
genus

node349.members.0.js
species
1
1639108

node350.members.0.js
613
6
14439
genus

145
615
species
node351.members.0.js

node352.members.0.js
species
82996
3

1
61652
species
node353.members.0.js

node354.members.0.js
species
47917
14281

28151
1
species
node355.members.0.js

node356.members.0.js
614
2
species

34037
1
genus

species
1805933
1
node358.members.0.js

genus
7
629

node360.members.0.js
4
630
species

3
1649845
species group

node362.members.0.js
species
633
3

1
28
1903409
family
node363.members.0.js

53335
19
6
genus
node364.members.0.js

1
1891675
species
node365.members.0.js

node366.members.0.js
species
592316
2

3
1484157
species
node367.members.0.js

1
1235990
species
node368.members.0.js

node369.members.0.js
species
1
1484158

species
470934
5
node370.members.0.js

551
4
genus

node372.members.0.js
species
215689
3

node373.members.0.js
species
182337
1

genus
4
2100764

node375.members.0.js
665914
4
species

135619
7
order

family
1
135620

genus
1
28253

node379.members.0.js
400668
1
species

node380.members.0.js
family
5
1
28256

1
2745
genus

species
1
1897729
node382.members.0.js

404432
2
genus

node384.members.0.js
2
1771309
species

genus
1
204286

node386.members.0.js
species
28258
1

family
1
224379

158481
1
genus

node389.members.0.js
158327
1
species

3
135613
order

72276
1
family

1335745
1
genus

species
1
1335757
node393.members.0.js

family
1
449719

genus
1
437504

species
437505
1
node396.members.0.js

family
1046
1
node397.members.0.js

order
1
118969

family
1
444

genus
445
1

node401.members.0.js
species
1
446

72273
1
order

135616
1
family

1
28884
genus

node405.members.0.js
species
39765
1

135623
10
order

family
10
641

genus
2
657

node409.members.0.js
1
38293
species

node410.members.0.js
species
1
1295392

genus
7
662

node412.members.0.js
species
1
55601

717610
3
species group

696485
2
species
node414.members.0.js

691
1
species
node415.members.0.js

node416.members.0.js
species
3
666

246861
1
genus

673
1
species
node418.members.0.js

class
28216
263

order
206389
3

family
75787
1

genus
1
146937

species
146939
1
node423.members.0.js

family
2
2008794

1
12960
genus

species
748247
1
node426.members.0.js

genus
33057
1

species
2005884
1
node428.members.0.js

order
206351
63

1499392
61
family

90153
61
no rank

genus
535
61

node433.members.0.js
1108595
1
species

node434.members.0.js
536
60
species

481
2
family

genus
59
1

1
96942
species
node437.members.0.js

genus
1
538

node439.members.0.js
539
1
species

2
32003
order

2008790
1
family

919
1
genus

node443.members.0.js
species
1
36861

family
206379
1

genus
1
914

1
915
species
node446.members.0.js

195
80840
order

172
506
family

1
290425
genus

node450.members.0.js
302406
1
species

genus
2
517

species
1416803
1
node452.members.0.js

1697043
1
species
node453.members.0.js

genus
222
2
169
node454.members.0.js

85698
167
species
node455.members.0.js

no rank
4
119065

no rank
2
80841

node458.members.0.js
species
2
1834205

no rank
224471
2

genus
1
28067

species
28068
1
node461.members.0.js

32012
1
genus

species
926
1
node463.members.0.js

node464.members.0.js
family
1
4
80864

34072
2
genus

1
2126319
species
node466.members.0.js

1
34073
species
node467.members.0.js

283
1
genus

species
285
1
node469.members.0.js

family
2
75682

genus
1
149698

node472.members.0.js
species
2045208
1

963
1
genus

node474.members.0.js
species
1
341045

119060
13
family

genus
2
1
106589
node476.members.0.js

1
119219
species
node477.members.0.js

node478.members.0.js
genus
32008
6
3

2
87882
species group

node480.members.0.js
species
2
95486

758793
1
species
node481.members.0.js

1
93217
genus

species
93218
1
node483.members.0.js

1822464
2
4
genus
node484.members.0.js

1
75105
species
node485.members.0.js

node486.members.0.js
species
1
412963

1783272
145850
no rank

phylum
1239
61770
2
node488.members.0.js

526524
3
class

526525
3
order

3
128827
family

genus
191303
3

node493.members.0.js
1712675
3
species

class
6
186801

order
2
68295

1
186814
family

genus
249529
1

911092
1
species
node498.members.0.js

family
1
543372

252965
1
genus

species
1
252966
node501.members.0.js

186802
4
order

family
31979
1

1485
1
genus

node505.members.0.js
species
1
84022

543349
1
family

2733
1
genus

species
1
2734
node508.members.0.js

family
1
186804

1870884
1
genus

1
1496
species
node511.members.0.js

1
186803
family

1506553
1
genus

node514.members.0.js
species
1834196
1

node515.members.0.js
class
91061
61757
5

186826
9
order

1300
3
family

1301
3
genus

node519.members.0.js
species
1307
1

species
1
1335
node520.members.0.js

species
400065
1
node521.members.0.js

family
33958
3

3
1578
genus

node524.members.0.js
1545702
1
species

node525.members.0.js
species
1624
1

node526.members.0.js
1601
1
species

1
81850
family

genus
1243
1

node529.members.0.js
species
979982
1

family
81852
2

1
33969
genus

node532.members.0.js
33970
1
species

node533.members.0.js
1350
1
genus

node534.members.0.js
order
1385
61743
5

node535.members.0.js
61696
3
186817
family

genus
1
459532

species
1
586416
node537.members.0.js

1055323
3
genus

node539.members.0.js
species
3
33936

61683
27795
1386
genus
node540.members.0.js

1792192
1
species group

node542.members.0.js
1
293387
species

node543.members.0.js
species
1
1178537

node544.members.0.js
species
12
1409

node545.members.0.js
1
2009331
species

node546.members.0.js
species
7
1837130

node547.members.0.js
species
1856406
367

species
4
2049935
node548.members.0.js

2
1628753
species
node549.members.0.js

1479
1
species
node550.members.0.js

node551.members.0.js
2
199441
species

node552.members.0.js
species
3
1404

node553.members.0.js
1408
5
species

86664
1
species
node554.members.0.js

node555.members.0.js
species
1570330
1

species group
653685
33078

22
1938374
species subgroup

node558.members.0.js
species
18
492670

node559.members.0.js
1390
4
species

32083
1402
species
node560.members.0.js

15
1423
species
node561.members.0.js

node562.members.0.js
72361
1
species

species
1452
3
node563.members.0.js

node564.members.0.js
species
925
1648923

node565.members.0.js
species
119858
29

species
2
33932
node566.members.0.js

node567.members.0.js
species
561879
4

1398
1
species
node568.members.0.js

node569.members.0.js
species
309
2026248

species
1
324767
node570.members.0.js

node571.members.0.js
species
1664069
45

species
86665
1
node572.members.0.js

species group
39
86661

species
1428
2
node574.members.0.js

1
1392
species
node575.members.0.js

species
1396
36
node576.members.0.js

2
45667
genus

species
1570
1
node578.members.0.js

node579.members.0.js
402384
1
species

genus
84406
1

species
1
163877
node581.members.0.js

129337
3
genus

species group
2
1505648

species
33938
2
node584.members.0.js

species
1
33940
node585.members.0.js

family
15
1
186818
node586.members.0.js

11
1372
genus

node588.members.0.js
species
2058136
10

1
1215089
species
node589.members.0.js

3
648800
genus

node591.members.0.js
species
3
76853

16
186822
family

1
85151
no rank

1
55079
genus

1
1500254
species
node595.members.0.js

genus
1
15
44249
node596.members.0.js

node597.members.0.js
160799
1
species

1
1616788
species
node598.members.0.js

node599.members.0.js
species
2069255
1

node600.members.0.js
species
9
189426

node601.members.0.js
1
528191
species

node602.members.0.js
1870820
1
species

family
8
90964

node604.members.0.js
1279
8
1
genus

node605.members.0.js
species
1282
1

species
29385
6
node606.members.0.js

family
2
186820

genus
1
1637

species
1
1639
node609.members.0.js

2755
1
genus

node611.members.0.js
species
1
2756

186823
1
family

genus
29330
1

species
1
405212
node614.members.0.js

class
909932
1

order
1
1843489

family
31977
1

genus
1
906

1
907
species
node619.members.0.js

1737404
1
class

order
1
1737405

family
2042895
1

genus
1505664
1

species
1
1556
node624.members.0.js

phylum
1297
1

class
1
188787

68933
1
order

188786
1
family

genus
1
65551

1
52022
species
node630.members.0.js

node631.members.0.js
544448
2
1
phylum

1
31969
class

order
186328
1

family
1
33925

1
46238
genus

species
215578
1
node636.members.0.js

phylum
201174
84072
1
node637.members.0.js

class
1
84998

1643822
1
order

1643826
1
family

genus
1
644652

node642.members.0.js
1
1841863
species

node643.members.0.js
10
84069
1760
class

order
85007
83642

family
1
85025

genus
1
1817

node647.members.0.js
37326
1
species

family
1653
83636

node649.members.0.js
1716
83636
2282
genus

152794
1
species
node650.members.0.js

node651.members.0.js
species
1717
1

species
80883
1718
node652.members.0.js

node653.members.0.js
species
571915
1

node654.members.0.js
species
14
1652495

node655.members.0.js
species
1
1072256

node656.members.0.js
species
92706
415

node657.members.0.js
1
349751
species

3
1050174
species
node658.members.0.js

node659.members.0.js
18
1721
species

species
1
161896
node660.members.0.js

node661.members.0.js
1
1121358
species

node662.members.0.js
species
161899
1

species
28028
1
node663.members.0.js

node664.members.0.js
species
1
1223514

species
1
161895
node665.members.0.js

1
1230998
species
node666.members.0.js

1
160386
species
node667.members.0.js

species
1408191
8
node668.members.0.js

5
1762
family

3
1866885
genus

node671.members.0.js
1
1797
species

species
1
1800
node672.members.0.js

node673.members.0.js
1
758802
species

node674.members.0.js
2
1763
genus

order
2
85012

2
83676
family

genus
2
2013

species
2014
2
node678.members.0.js

order
85008
1

1
28056
family

genus
673534
1

species
2024580
1
node682.members.0.js

order
85010
1

family
2070
1

genus
1
2071

node686.members.0.js
1
103731
species

398
85006
order

family
1268
393

genus
2
1663

node690.members.0.js
species
1
1849032

37928
1
species
node691.members.0.js

1742989
1
genus

node693.members.0.js
species
1
256701

genus
390
1269

species
390
1270
node695.members.0.js

node696.members.0.js
85023
1
2
family

1
2034
genus

node698.members.0.js
1
1905847
species

family
85017
2

genus
1
254250

node701.members.0.js
species
139208
1

genus
1
186188

1
186189
species
node703.members.0.js

85016
1
family

genus
1707
1

species
11
1
node706.members.0.js

order
11
85011

2062
11
family

node709.members.0.js
3
11
1883
genus

species
68570
1
node710.members.0.js

species
1
68202
node711.members.0.js

node712.members.0.js
1
1812480
species

node713.members.0.js
1
1889
species

1477431
1
no rank

species
42239
1
node715.members.0.js

species
1
1915
node716.members.0.js

1
67267
species
node717.members.0.js

node718.members.0.js
species
1
1265601

order
1
85013

family
1
74712

genus
1854
1

node722.members.0.js
species
298654
1

order
85009
3

31957
3
family

genus
72763
1

node726.members.0.js
species
399497
1

genus
1
1743

species
1
1744
node728.members.0.js

1912216
1
genus

node730.members.0.js
species
1
1747

1752188
1
no rank

1848755
1
species
node732.members.0.js

3
1798711
no rank

3
1117
phylum

order
1161
2

family
1185
2

1186
2
genus

species
1
2005462
node738.members.0.js

1
1954171
species
node739.members.0.js

1
1301283
subclass

1
1150
order

1892249
1
family

1
43988
genus

node744.members.0.js
species
43989
1

phylum
2
200795

1
292625
class

order
1
292629

292628
1
family

genus
233189
1

node750.members.0.js
167964
1
species

class
301297
1

genus
670486
1

1
552810
species
node753.members.0.js
