# Supplementary material for: Benchmarking the MinION: Evaluating long reads for microbial profiling
Source: Sci Rep. 2020 Mar 20;10:5125. doi: 10.1038/s41598-020-61989-x (PMC7083898; doi:10.1038/s41598-020-61989-x)
Supplement: Supplementary file 2 — Supplementary information2. [file 41598_2020_61989_MOESM2_ESM.zip › sample_barcode_2/kraken.html]

Javascript must be enabled to view this page.

members
magnitude
magnitudeUnassigned
count
unassigned
taxon
rank

BC2\_kraken\_krona

47
node0.members.0.js
261833

node1.members.0.js
5591

superkingdom
256194
node2.members.0.js
2
25

112867
node3.members.0.js
phylum
21
1224

node4.members.0.js
112619
class
31
1236

1
135619
order

1
28256
family

1
2745
genus

1666906
1
node8.members.0.js
species

112030
node9.members.0.js
order
342
91347

family
1903409
5

82986
genus
1

53336
1
node12.members.0.js
species

53335
2
genus
node13.members.0.js
3

1
node14.members.0.js
species
553

1
32199
genus

9
species
1

118110
no rank
1
node17.members.0.js

5
1903412
family

genus
5
node19.members.0.js
635
1

species
node20.members.0.js
3
67780

93378
species
1
node21.members.0.js

family
node22.members.0.js
14097
1903411
2

9
613
node23.members.0.js
14092
genus

2
82996
species

1006598
2
node25.members.0.js
no rank

14081
node26.members.0.js
species
47917

629
genus
2

species group
1649845
1

species
node29.members.0.js
1
633

935293
node30.members.0.js
1
species

genus
1745211
1

species
1639108
1

1
node33.members.0.js
no rank
1441930

543
1096
family
node34.members.0.js
97570

191675
no rank
2

no rank
2
node36.members.0.js
84563
1

146507
no rank
1

1
genus
568987

species
138072
1

572265
node40.members.0.js
1
no rank

node41.members.0.js
138
genus
13
570

1134687
species
3
node42.members.0.js

species
node43.members.0.js
2
2026240

1905288
node44.members.0.js
34
species

36
573
node45.members.0.js
56
species

no rank
node46.members.0.js
2
1365186

72407
subspecies
node47.members.0.js
18

571
species
22
node48.members.0.js

1463165
species
8
node49.members.0.js

3
83654
genus

species
node51.members.0.js
3
83655

1
1335483
genus

563
species
1

no rank
node54.members.0.js
1
630626

18
590
genus

28901
8
species
17
node56.members.0.js

9
node57.members.0.js
subspecies
1
59201

no rank
1
node58.members.0.js
108619

1
90370
node59.members.0.js
2
no rank

527001
node60.members.0.js
1
no rank

no rank
115981
2

2
node62.members.0.js
no rank
859199

90371
no rank
3
node63.members.0.js

54736
species
node64.members.0.js
1

2
genus
579

61648
2
node66.members.0.js
species

genus
1330545
1

species
1
node68.members.0.js
1907578

1
158483
genus

species
1
node70.members.0.js
158822

20
544
genus

node72.members.0.js
3
species
2
35703

node73.members.0.js
1
no rank
1261127

67824
species
node74.members.0.js
4

1
1344959
node75.members.0.js
13
species group

546
species
node76.members.0.js
11

57706
species
node77.members.0.js
1

221
genus
561

564
species
node79.members.0.js
1

158
562
node80.members.0.js
220
species

no rank
2
node81.members.0.js
696406

node82.members.0.js
6
no rank
1358422

no rank
1038927
8

node84.members.0.js
4
no rank
1133853

1048254
no rank
node85.members.0.js
4

3
node86.members.0.js
no rank
585057

585397
no rank
14
node87.members.0.js

1329907
no rank
1
node88.members.0.js

758831
no rank
2
node89.members.0.js

244319
no rank
1

573235
no rank
1
node91.members.0.js

405955
2
node92.members.0.js
no rank

no rank
node93.members.0.js
1
1078034

3
no rank
498388

3
node95.members.0.js
no rank
481805

no rank
1603259
2

331111
node97.members.0.js
2
no rank

node98.members.0.js
7
no rank
199310

1
node99.members.0.js
no rank
1050617

1
83334
node100.members.0.js
3
no rank

1328859
1
node101.members.0.js
no rank

1
node102.members.0.js
no rank
155864

439855
no rank
2
node103.members.0.js

930406
node104.members.0.js
2
no rank

910348
no rank
1
node105.members.0.js

861906
no rank
1

node107.members.0.js
1
no rank
216592

929812
genus
1

929813
species
1
node109.members.0.js

11
620
genus

1
623
2
node111.members.0.js
species

node112.members.0.js
1
no rank
1282358

node113.members.0.js
4
species
624

4
622
species

no rank
node115.members.0.js
4
300267

species
621
1

344609
no rank
node117.members.0.js
1

1
genus
409304

1
168169
species

no rank
node120.members.0.js
1
476281

1
160674
node121.members.0.js
33
genus

node122.members.0.js
3
species
54291

node123.members.0.js
29
species
575

genus
5610
node124.members.0.js
547
12

1692238
species
1
node125.members.0.js

2860
354276
5596
node126.members.0.js
species group

61645
21
species
58
node127.members.0.js

node128.members.0.js
7
no rank
1421338

640513
30
node129.members.0.js
no rank

299767
5
node130.members.0.js
species

1812935
4
node131.members.0.js
species

node132.members.0.js
434
species
380
550

no rank
node133.members.0.js
1
1333850

node134.members.0.js
7
no rank
1354030

35
subspecies
69219

1104326
no rank
35
node136.members.0.js

1045856
no rank
node137.members.0.js
3

336306
subspecies
8

no rank
2
node139.members.0.js
1211025

6
node140.members.0.js
no rank
716541

species
7
node141.members.0.js
1915310

208224
species
node142.members.0.js
9

node143.members.0.js
2219
species
110
158836

301105
node144.members.0.js
154
subspecies

299766
node145.members.0.js
1801
subspecies

1296536
23
node146.members.0.js
subspecies

26
node147.members.0.js
subspecies
1812934

node148.members.0.js
105
subspecies
301102

399742
1
node149.members.0.js
species

11
1330546
genus

node151.members.0.js
11
species
9
1334193

701347
node152.members.0.js
2
no rank

2346
413496
90400
node153.members.0.js
genus

40
species
413502

693216
no rank
40
node155.members.0.js

7
535744
species

node157.members.0.js
7
no rank
1074000

12
1163710
species

1073999
no rank
12
node159.members.0.js

13
species
413501

1159613
no rank
node161.members.0.js
13

413497
species
10

413498
subspecies
10

1159554
no rank
10
node164.members.0.js

species
node165.members.0.js
87924
28141
79644

290339
4119
node166.members.0.js
no rank

no rank
node167.members.0.js
306
956149

1138308
node168.members.0.js
3855
no rank

48
node169.members.0.js
species
19
413503

no rank
node170.members.0.js
29
1159491

3
family
1903414

1
626
node172.members.0.js
3
genus

2
node173.members.0.js
species
1
628

1
node174.members.0.js
no rank
406817

8
family
1903410

4
204037
7
node176.members.0.js
genus

1089444
node177.members.0.js
3
species

84565
genus
1

1929246
node179.members.0.js
1
species

550
order
135614

550
32033
family

338
6
genus
node182.members.0.js
550

52
339
node183.members.0.js
543
species

411
340
node184.members.0.js
489
no rank

1281282
3
node185.members.0.js
no rank

314565
3
node186.members.0.js
no rank

node187.members.0.js
72
no rank
190485

no rank
1
node188.members.0.js
92826

359385
no rank
1
node189.members.0.js

species
1
node190.members.0.js
56460

72274
order
3

1
family
468

469
genus
1

909768
species group
1

470
species
1
node195.members.0.js

2
135621
family

genus
286
2

1
136841
species group

287
1
node199.members.0.js
species

node200.members.0.js
1
species
1283291

order
1706369
1

1
1706371
family

genus
447467
1

1
species
447471

node205.members.0.js
1
no rank
1117647

2
order
135622

2
family
72275

1
node208.members.0.js
genus
2742

genus
226
1

314275
node210.members.0.js
1
species

135613
order
1

1046
1
node212.members.0.js
family

3
class
28211

order
3
node214.members.0.js
356
1

41294
1
node215.members.0.js
family

1
82115
family

1
no rank
227290

1
genus
379

2028343
species
1
node219.members.0.js

1
28216
224
node220.members.0.js
class

59
order
206351

59
1499392
family

90153
no rank
59

59
genus
535

59
species
536

no rank
node226.members.0.js
59
243365

164
80840
order

1
family
119060

genus
48736
1

105219
species
node230.members.0.js
1

family
506
163

222
1
genus
node232.members.0.js
163

162
node233.members.0.js
species
136
85698

no rank
node234.members.0.js
10
1167634

node235.members.0.js
13
no rank
562971

no rank
3
node236.members.0.js
762376

1783272
2
no rank
node237.members.0.js
143302

83066
phylum
201174

12
1760
node239.members.0.js
83066
class

85007
1
order
node240.members.0.js
82669

82668
1653
family

1716
6982
genus
node242.members.0.js
82668

2
species
349751

1224162
2
node244.members.0.js
no rank

1
1717
node245.members.0.js
2
species

698964
1
node246.members.0.js
no rank

species
node247.members.0.js
1
161896

1050174
node248.members.0.js
3
species

1
species
38288

no rank
node250.members.0.js
1
585529

species
203263
1

no rank
1
node252.members.0.js
1431546

1652495
species
node253.members.0.js
36

43770
species
1
node254.members.0.js

14
species
1408191

931089
node256.members.0.js
14
no rank

4
42817
species

node258.members.0.js
4
no rank
1348662

571915
species
node259.members.0.js
1

38302
species
node260.members.0.js
2

node261.members.0.js
1
species
136857

72054
1718
75576
node262.members.0.js
species

1079988
no rank
497
node263.members.0.js

node264.members.0.js
105
no rank
1310161

340322
no rank
node265.members.0.js
1829

no rank
1090
node266.members.0.js
196627
976

1204414
114
node267.members.0.js
no rank

1
node268.members.0.js
no rank
1232384

1705
node269.members.0.js
1
species

1
species
160386

1285583
no rank
1
node271.members.0.js

65058
1
species
2
node272.members.0.js

945711
node273.members.0.js
1
no rank

species
node274.members.0.js
1
191610

1721
species
10

no rank
node276.members.0.js
10
1121353

3
169292
species

no rank
3
node278.members.0.js
548476

11
species
92706

1232385
11
node280.members.0.js
no rank

7
152794
species

no rank
node282.members.0.js
7
196164

28028
2
node283.members.0.js
species

node284.members.0.js
3
species
146827

85006
order
384

1
1268
node286.members.0.js
384
family

genus
1269
383

species
node288.members.0.js
383
1270
345

38
node289.members.0.js
no rank
465515

1
order
85012

1
family
83676

1
2013
genus

1
2014
species

subspecies
568208
1

446468
no rank
node295.members.0.js
1

2
1239
60234
node296.members.0.js
phylum

12
91061
node297.members.0.js
60232
class

order
60218
node298.members.0.js
1385
10

family
186822
9

44249
genus
9

189426
node301.members.0.js
9
species

8
186817
60191
node302.members.0.js
family

129337
genus
1

species group
1505648
1

33938
1
node305.members.0.js
species

genus
60182
node306.members.0.js
1386
26433

1774743
1
node307.members.0.js
species

species
2
node308.members.0.js
79880

3
node309.members.0.js
species
1398

2
300825
species

no rank
node311.members.0.js
2
1246626

665099
species
3

1196031
node313.members.0.js
3
no rank

98228
species
1
node314.members.0.js

135735
species
1
node315.members.0.js

1837130
node316.members.0.js
8
species

1441095
species
node317.members.0.js
8

1628753
species
1
node318.members.0.js

species
3
node319.members.0.js
1479

1478
species
2
node320.members.0.js

5
86665
species

no rank
node322.members.0.js
5
272558

2
species
1471

2
node324.members.0.js
no rank
796606

86664
species
node325.members.0.js
7

species
node326.members.0.js
11
561879

305
node327.members.0.js
species group
34
86661

1392
10
species
node328.members.0.js
13

1392837
no rank
1
node329.members.0.js

no rank
2
node330.members.0.js
1449979

7
node331.members.0.js
species
1405

species
1
node332.members.0.js
1892404

species
74
node333.members.0.js
1428
39

4
29337
no rank

no rank
4
node335.members.0.js
930170

29339
12
no rank
25
node336.members.0.js

node337.members.0.js
4
no rank
1261129

node338.members.0.js
6
no rank
570416

node339.members.0.js
3
no rank
1279365

1
node340.members.0.js
no rank
180850

no rank
5
node341.members.0.js
1195464

1396
147
species
node342.members.0.js
176

no rank
node343.members.0.js
1
572264

1454382
no rank
node344.members.0.js
2

288681
node345.members.0.js
11
no rank

no rank
node346.members.0.js
2
1217984

2
node347.members.0.js
no rank
222523

361100
node348.members.0.js
1
no rank

no rank
4
node349.members.0.js
269801

node350.members.0.js
2
no rank
347495

451709
node351.members.0.js
3
no rank

1179100
no rank
1

no rank
1
node353.members.0.js
637380

1408
13
node354.members.0.js
species

1664069
node355.members.0.js
190
species

1
79885
species

398511
no rank
node357.members.0.js
1

2892
653685
33102
node358.members.0.js
species group

235
node359.members.0.js
species
119858

node360.members.0.js
125
species
73
1423

483913
node361.members.0.js
3
subspecies

86029
2
node362.members.0.js
subspecies

936156
no rank
node363.members.0.js
18

10
96241
subspecies

no rank
6
node365.members.0.js
655816

1052585
no rank
4
node366.members.0.js

19
node367.members.0.js
subspecies
6
135461

node368.members.0.js
4
no rank
535026

1404258
7
node369.members.0.js
no rank

node370.members.0.js
2
no rank
1052588

node371.members.0.js
55
species subgroup
8
1938374

species
27
node372.members.0.js
492670
13

1458206
no rank
9
node373.members.0.js

no rank
node374.members.0.js
1
1385727

no rank
4
node375.members.0.js
1338518

1390
7
species
node376.members.0.js
20

1333538
no rank
node377.members.0.js
1

692420
no rank
node378.members.0.js
4

no rank
2
node379.members.0.js
1034836

1292358
node380.members.0.js
6
no rank

node381.members.0.js
6
species
5
1452

node382.members.0.js
1
no rank
1239783

2364
1648923
node383.members.0.js
2494
species

766760
130
node384.members.0.js
no rank

1402
16598
species
node385.members.0.js
27295

no rank
node386.members.0.js
10653
279010

1126218
44
node387.members.0.js
no rank

node388.members.0.js
18
species
756828

species
2
node389.members.0.js
1547283

4
node390.members.0.js
species
1705566

species
2
node391.members.0.js
199441

1856406
node392.members.0.js
52
species

90964
family
8

8
1279
genus

29385
1
species
6
node395.members.0.js

subspecies
147452
5

342451
node397.members.0.js
5
no rank

1282
species
1
node398.members.0.js

1
species
1280

subspecies
46170
1

1006543
no rank
node401.members.0.js
1

2
186826
order

family
81852
1

1
1350
genus

1352
species
1

1305849
1
node406.members.0.js
no rank

1300
family
1

1
1301
genus

species
1308
1

no rank
1
node410.members.0.js
264199

1
superkingdom
10239

1
35237
no rank

1
order
28883

1
10662
family

1
no rank
196896

984186
1
node416.members.0.js
species
