# Supplementary material for: Benchmarking the MinION: Evaluating long reads for microbial profiling
Source: Sci Rep. 2020 Mar 20;10:5125. doi: 10.1038/s41598-020-61989-x (PMC7083898; doi:10.1038/s41598-020-61989-x)
Supplement: Supplementary file 2 — Supplementary information2. [file 41598_2020_61989_MOESM2_ESM.zip › sample_barcode_2/kraken2.html]

Javascript must be enabled to view this page.

members
magnitude
magnitudeUnassigned
count
unassigned
taxon
rank

BC2\_kraken2\_krona

18
node0.members.0.js
261833

superkingdom
10239
4

no rank
1
4
node2.members.0.js
35237

2
51368
no rank

2
2060084
genus

1349410
node5.members.0.js
1
species

1349409
species
node6.members.0.js
1

548681
1
order

family
548682
1

genus
1
692606

150286
species
node10.members.0.js
1

node11.members.0.js
2839

2157
2
superkingdom

phylum
1
28890

183968
1
class

2258
1
order

2259
1
family

genus
1
2260

species
1
53953

70601
1
node19.members.0.js
no rank

1783275
1
no rank

phylum
1
28889

183924
1
class

1
114380
order

2272
1
family

genus
1
477695

1
1200300
species

1
node27.members.0.js
no rank
1184251

superkingdom
25
258970
node28.members.0.js
2

1783257
5
no rank

3
node30.members.0.js
phylum
1
203682

2
203683
class

112
2
order

family
126
2

genus
1
123

species
1
125

1
node36.members.0.js
no rank
530564

1
118
genus

species
1
node38.members.0.js
1632864

74201
2
phylum

class
2
414999

2
415000
order

134623
2
family

1
178440
genus

species
1
107709

452637
1
node45.members.0.js
no rank

1
2028344
genus

species
1
node47.members.0.js
1796921

40117
2
phylum

class
203693
2

order
189778
2

family
2
189779

genus
2
1234

species
1
node53.members.0.js
42253

330214
species
node54.members.0.js
1

1224
node55.members.0.js
114004
28
phylum

1236
31
class
node56.members.0.js
113679

10
72274
order

family
135621
8

8
node59.members.0.js
genus
2
286

node60.members.0.js
1
species
797277

species
node61.members.0.js
1
1283291

1028989
species
1
node62.members.0.js

136841
1
species group

287
node64.members.0.js
1
species

2
136843
species group

380021
species
1
node66.members.0.js

294
species
1
node67.members.0.js

family
2
468

genus
2
469

species group
node70.members.0.js
1
909768

2004647
species
node71.members.0.js
1

135613
1
order

node73.members.0.js
1
family
1046

2
135624
order

family
2
84642

642
2
genus

1
node77.members.0.js
species
654

species
1
node78.members.0.js
1636607

order
5
135619

family
135620
1

28253
1
genus

1
936476
species

1
node83.members.0.js
no rank
491952

family
4
28256

2745
1
genus
node85.members.0.js
4

species
node86.members.0.js
1
1897729

475662
species
node87.members.0.js
1

1971364
species
node88.members.0.js
1

3
135623
order

family
3
641

genus
662
3

717610
1
species group

species subgroup
2315253
1

150340
1
node94.members.0.js
species

62153
node95.members.0.js
1
species

672
species
node96.members.0.js
1

order
554
135614

32033
node98.members.0.js
554
1
family

338
node99.members.0.js
551
3
genus

41
species
node100.members.0.js
547
339

node101.members.0.js
2
no rank
92826

340
411
no rank
node102.members.0.js
502

314565
no rank
node103.members.0.js
4

1358015
no rank
4
node104.members.0.js

1
node105.members.0.js
no rank
1358017

190485
no rank
node106.members.0.js
82

359385
2
node107.members.0.js
no rank

node108.members.0.js
1
species
56460

genus
40323
1

species group
1
995085

2072406
species
1
node111.members.0.js

1
2370
genus

node113.members.0.js
1
species
1444770

1706369
2
order

1
1706372
family

genus
393661
1

species
393662
1

314285
1
node118.members.0.js
no rank

1706371
1
family

genus
1
10

1
node121.members.0.js
species
1987723

135622
5
order

267890
2
family

22
2
node124.members.0.js
genus
1

271097
1
species

1
node126.members.0.js
no rank
425104

1
267888
family

genus
1
53246

227
1
species

1314868
1
node130.members.0.js
no rank

family
267889
1

genus
1
28228

1
node133.members.0.js
species
58049

family
1
267893

no rank
1
946227

1298881
species
node136.members.0.js
1

113066
node137.members.0.js
order
391
91347

family
11
1903409

53335
1
genus
node139.members.0.js
4

1
node140.members.0.js
species
1891675

species
node141.members.0.js
1
553

node142.members.0.js
1
species
1484158

1
genus
node143.members.0.js
5
551

2
182337
species

634500
node145.members.0.js
2
no rank

species
1
node146.members.0.js
1619313

species
node147.members.0.js
1
552

1
82986
genus

82987
species
1
node149.members.0.js

2100764
1
genus

665913
node151.members.0.js
1
species

family
5
1903412

568
1
genus

1
node154.members.0.js
species
569

4
635
genus

4
node156.members.0.js
species
67780

1
451511
no rank

447792
1
genus

1
node159.members.0.js
species
1756993

node160.members.0.js
98439
740
family
543

83654
genus
2
5
node161.members.0.js

3
node162.members.0.js
species
1920116

genus
5
42
node163.members.0.js
544

2
species group
node164.members.0.js
30
1344959

2077147
species
node165.members.0.js
2

2066049
species
1
node166.members.0.js

2077148
species
node167.members.0.js
1

67827
species
3
node168.members.0.js

546
species
21
node169.members.0.js

2
species
node170.members.0.js
7
35703

1261127
no rank
node171.members.0.js
5

genus
579
1

61648
node173.members.0.js
1
species

570
22
genus
node174.members.0.js
222

1134687
species
5
node175.members.0.js

573
75
species
node176.members.0.js
120

subspecies
39831
2

2
node178.members.0.js
no rank
861365

1
node179.members.0.js
no rank
1365186

no rank
1
node180.members.0.js
1049565

72407
41
node181.members.0.js
subspecies
35

1225181
6
node182.members.0.js
no rank

node183.members.0.js
25
24
species
571

node184.members.0.js
1
no rank
1333852

2026240
species
2
node185.members.0.js

species
1
node186.members.0.js
244366

1463165
12
node187.members.0.js
species

species
node188.members.0.js
2
548

1905288
species
node189.members.0.js
33

1
929812
genus

1
node191.members.0.js
species
929813

genus
1
5
node192.members.0.js
1330547

208223
4
node193.members.0.js
species

413496
91201
node194.members.0.js
genus
1779

65
node195.members.0.js
species
33
413503

1159491
node196.members.0.js
32
no rank

species
22
1163710

22
node198.members.0.js
no rank
1073999

413497
16
species

16
413498
subspecies

1159554
16
node201.members.0.js
no rank

30
535744
species

no rank
30
node203.members.0.js
1074000

species
413501
20

1159613
no rank
20
node205.members.0.js

species
79053
89184
node206.members.0.js
28141

no rank
647
node207.members.0.js
956149

290339
no rank
466
node208.members.0.js

1138308
no rank
node209.members.0.js
9018

species
85
413502

no rank
node211.members.0.js
85
693216

genus
1330545
5

species
node213.members.0.js
3
1907578

61646
species
node214.members.0.js
1

species
1
node215.members.0.js
2153385

4
1330546
genus

61647
species
3
node217.members.0.js

1334193
node218.members.0.js
1
species

590
genus
1
57
node219.members.0.js

54736
species
node220.members.0.js
1

28901
55
node221.members.0.js
species
32

59201
node222.members.0.js
20
3
subspecies

90371
no rank
2
node223.members.0.js

1077085
1
node224.members.0.js
no rank

no rank
1
440524

no rank
1
node226.members.0.js
866913

no rank
node227.members.0.js
1
28150

119912
no rank
node228.members.0.js
1

28144
node229.members.0.js
1
no rank

90370
no rank
1
node230.members.0.js

no rank
node231.members.0.js
3
149539

108619
3
node232.members.0.js
no rank

54388
node233.members.0.js
2
no rank

58712
node234.members.0.js
1
no rank

subspecies
2
3
node235.members.0.js
59202

1
1243601
no rank

1
node237.members.0.js
no rank
1243602

620
5
genus

622
5
species

node240.members.0.js
5
no rank
300267

genus
1335483
2

563
2
species

630626
2
node243.members.0.js
no rank

no rank
191675
9

no rank
84563
1

genus
1906657
1

1
node247.members.0.js
species
1778262

no rank
8
36866

2282309
species
node249.members.0.js
4

1
node250.members.0.js
species
2282310

1920109
node251.members.0.js
3
species

547
82
genus
node252.members.0.js
5756

species
node253.members.0.js
1
1692238

node254.members.0.js
2
species
1977566

1560339
1
node255.members.0.js
species

5659
node256.members.0.js
species group
1803
354276

158836
node257.members.0.js
2536
218
species

node258.members.0.js
305
subspecies
301105

1812934
subspecies
66
67
node259.members.0.js

no rank
node260.members.0.js
1
1333851

1296536
subspecies
node261.members.0.js
57

subspecies
node262.members.0.js
1887
299766

2
node263.members.0.js
subspecies
301102

550
772
node264.members.0.js
species
666

336306
30
subspecies

no rank
7
node266.members.0.js
1211025

716541
no rank
node267.members.0.js
23

1354030
node268.members.0.js
8
no rank

61
69219
subspecies

no rank
node270.members.0.js
61
1104326

no rank
5
node271.members.0.js
1045856

node272.members.0.js
2
no rank
1333850

species
255
node273.members.0.js
2027919

61645
36
species
node274.members.0.js
129

no rank
79
node275.members.0.js
640513

1421338
node276.members.0.js
14
no rank

113
node277.members.0.js
species
69218

2077137
species
node278.members.0.js
8

node279.members.0.js
6
species
299767

species
2
node280.members.0.js
2077136

208224
species
6
node281.members.0.js

species
6
node282.members.0.js
1915310

node283.members.0.js
23
species
1812935

399742
species
node284.members.0.js
5

1914861
4
node285.members.0.js
species

881260
node286.members.0.js
2
species

2
158483
genus

2
node288.members.0.js
species
158822

34
160674
genus

54291
2
node290.members.0.js
species

575
species
32
node291.members.0.js

genus
3
348
node292.members.0.js
561

node293.members.0.js
337
216
species
562

node294.members.0.js
1
no rank
1954351

26
node295.members.0.js
no rank
1050617

8
node296.members.0.js
no rank
2048777

585397
no rank
6
node297.members.0.js

585057
no rank
node298.members.0.js
6

585055
no rank
1
node299.members.0.js

168807
no rank
node300.members.0.js
2

83334
1
no rank
node301.members.0.js
3

no rank
node302.members.0.js
1
1328859

155864
1
node303.members.0.js
no rank

83333
4
node304.members.0.js
no rank

1446746
no rank
node305.members.0.js
1

node306.members.0.js
12
no rank
199310

node307.members.0.js
26
no rank
405955

node308.members.0.js
7
no rank
930406

11
1038927
no rank

node310.members.0.js
2
no rank
1134782

1048254
7
node311.members.0.js
no rank

1133852
no rank
node312.members.0.js
2

2048781
no rank
5
node313.members.0.js

2
861906
no rank

no rank
node315.members.0.js
2
216592

1499973
4
node316.members.0.js
species

208962
node317.members.0.js
4
species

family
1
1903414

genus
583
1

species
node320.members.0.js
1
585

14199
node321.members.0.js
family
3
1903411

genus
1745211
1

1
1639108
species

1441930
node324.members.0.js
1
no rank

613
genus
6
14191
node325.members.0.js

47917
node326.members.0.js
14181
species

615
node327.members.0.js
2
species

61652
2
node328.members.0.js
species

genus
629
4

species group
1649845
3

2
species
node331.members.0.js
3
633

1
109458
no rank

no rank
node333.members.0.js
1
748672

28152
species
node334.members.0.js
1

family
19
1903410

2
71655
genus

598467
species
2
node337.members.0.js

genus
2
122277

1
554
species

555
1
subspecies

561230
no rank
node341.members.0.js
1

species
node342.members.0.js
1
29471

genus
1082702
1

1082704
node344.members.0.js
1
species

genus
3
14
node345.members.0.js
204037

2
node346.members.0.js
species
1778540

556
1
species

1223569
no rank
node348.members.0.js
1

node349.members.0.js
6
species
1089444

204039
node350.members.0.js
2
species

28211
36
class

order
204457
11

1
335929
family

genus
361177
1

species
node355.members.0.js
1
645517

10
41297
family

genus
1
1434046

species
1
node358.members.0.js
2077182

genus
72173
1

1634516
species
1
node360.members.0.js

1
165696
genus

species
48935
1

279238
no rank
node363.members.0.js
1

165697
2
node364.members.0.js
genus
1

1357916
1
node365.members.0.js
species

2
165695
genus

1
46429
species

no rank
1
node368.members.0.js
690566

1855519
node369.members.0.js
1
species

genus
13687
3

941907
node371.members.0.js
1
species

93064
species
2
node372.members.0.js

order
356
15

family
255475
2

293088
1
genus

686597
species
node376.members.0.js
1

genus
414371
1

1349819
node378.members.0.js
1
species

3
45401
family

1
46913
genus

1736675
1
node381.members.0.js
species

81
1
genus

species
node383.members.0.js
1
717785

1
29407
genus

species
node385.members.0.js
1
674703

family
82115
3

no rank
227292
2

28105
2
genus

382
species
1
node389.members.0.js

663276
1
no rank

380
1
species

1185652
no rank
1
node392.members.0.js

1
227290
no rank

1
357
genus

1
node395.members.0.js
species
359

family
69277
1

genus
449972
1

266779
species
node398.members.0.js
1

no rank
119042
1

genus
1734920
1

1235591
node401.members.0.js
1
species

family
1
772

genus
773
1

803
species
node404.members.0.js
1

family
2036754
1

genus
1
node406.members.0.js
28209

family
41294
3

node408.members.0.js
3
1
genus
374

335659
1
node409.members.0.js
species

1
node410.members.0.js
species
1437360

order
5
204455

1
69657
family

genus
1
85

1873716
1
node414.members.0.js
species

family
4
31989

genus
285107
1

1915078
species
1
node417.members.0.js

genus
97050
2

1
node419.members.0.js
species
2099786

292414
node420.members.0.js
1
species

265
1
genus

34004
species
node422.members.0.js
1

5
204441
order

433
2
family

1
89583
genus

1
33996
species

no rank
node427.members.0.js
1
272568

1
441
genus

1
442
species

1288313
no rank
1
node430.members.0.js

41295
3
family

genus
1
13134

55518
1
node433.members.0.js
species

191
2
node434.members.0.js
genus
1

species
1
node435.members.0.js
1226968

5
68525
subphylum

3
28221
class

order
213118
1

family
213121
1

genus
109168
1

species
84980
1

177439
no rank
1
node442.members.0.js

order
29
1

80812
1
suborder

family
49
1

genus
50
1

52
species
1
node447.members.0.js

order
69541
1

213422
1
family

28231
genus
1
node450.members.0.js

2
29547
class

213849
2
order

72294
2
family

genus
194
1

node455.members.0.js
1
species
199

no rank
2321108
1

genus
1
28196

1032072
1
species

node459.members.0.js
1
no rank
870501

256
node460.members.0.js
class
1
28216

order
80840
192

119065
2
no rank

1
80841
no rank

1469502
species
1
node464.members.0.js

no rank
224471
1

genus
28067
1

28068
1
species

1
node468.members.0.js
no rank
983917

3
80864
family

28065
1
genus

192843
1
species

node472.members.0.js
1
no rank
338969

1
238749
genus

1
node474.members.0.js
species
1546149

1
34072
genus

1795631
node476.members.0.js
1
species

family
1
11
node477.members.0.js
119060

106589
genus
1
3
node478.members.0.js

node479.members.0.js
2
species
96344

1
48736
genus

1
305
species

859655
1
node482.members.0.js
no rank

1822464
node483.members.0.js
1
genus

93217
1
genus

species
node485.members.0.js
1
93222

1
44013
genus

species
node487.members.0.js
1
556054

genus
32008
3

species group
87882
1

265293
node490.members.0.js
1
species

111527
2
species group

342113
1
node492.members.0.js
species

57975
species
1
node493.members.0.js

family
5
75682

1
29580
genus

node496.members.0.js
1
species
1938606

4
202907
genus

1
node498.members.0.js
species
158899

species
3
node499.members.0.js
279058

506
171
family

517
1
genus

1746199
1
node502.members.0.js
species

169
node503.members.0.js
genus
1
222

217203
species
1
node504.members.0.js

node505.members.0.js
167
144
species
85698

1167634
no rank
node506.members.0.js
11

no rank
4
node507.members.0.js
762376

562971
8
node508.members.0.js
no rank

1
507
genus

323284
species
node510.members.0.js
1

order
1
32003

1
32011
family

1
16
genus

node514.members.0.js
1
species
1662285

206389
3
order

2008794
family
1
3
node516.members.0.js

12960
1
genus
node517.members.0.js
2

748247
1
node518.members.0.js
species

order
206351
59

family
1499392
59

no rank
59
90153

535
59
node522.members.0.js
genus
1

58
node523.members.0.js
species
3
536

55
node524.members.0.js
no rank
243365

203691
1
phylum

203692
1
class

order
136
1

137
1
family

146
1
genus

species
node530.members.0.js
1
154

5
no rank
node531.members.0.js
144920
1783272

1297
3
phylum

class
3
188787

order
3
118964

183710
3
family

genus
3
1298

1
309887
species

no rank
node538.members.0.js
1
709986

node539.members.0.js
1
species
980427

1
node540.members.0.js
species
2202254

201174
83686
phylum

node542.members.0.js
83684
15
class
1760

2
85012
order

1
2012
family

1
1988
genus

1
node546.members.0.js
species
1411117

family
83676
1

genus
2013
1

species
280236
1

no rank
1
node550.members.0.js
1235441

85007
node551.members.0.js
83263
1
order

family
1762
2

1763
genus
node553.members.0.js
1

1866885
1
genus

species
1800
1

710421
no rank
node556.members.0.js
1

1653
83258
family

1716
genus
4639
83258
node558.members.0.js

26
1721
species

26
node560.members.0.js
no rank
1121353

35755
species
node561.members.0.js
2

169292
5
species

548476
node563.members.0.js
5
no rank

187491
node564.members.0.js
2
species

441500
node565.members.0.js
1
species

node566.members.0.js
3
species
161899

1697
4
node567.members.0.js
species

161895
3
node568.members.0.js
species

1724
species
2
node569.members.0.js

1050174
node570.members.0.js
4
species

1072256
node571.members.0.js
2
species

species
4
node572.members.0.js
2079535

species
3
161879

645127
no rank
node574.members.0.js
3

8
species
node575.members.0.js
10
1717

2
1806053
no rank

node577.members.0.js
2
no rank
1450520

1
1223514
species

1223515
no rank
node579.members.0.js
1

146827
species
node580.members.0.js
9

1
43771
species

no rank
node582.members.0.js
1
1267754

species
3
node583.members.0.js
1705

349751
3
species

node585.members.0.js
3
no rank
1224162

65058
species
node586.members.0.js
1

4
38305
species

no rank
4
node588.members.0.js
1224164

2
1121358
species

558173
no rank
node590.members.0.js
2

species
5
node591.members.0.js
43770

1
191493
species

node593.members.0.js
1
no rank
1437874

258224
3
species

662755
3
node595.members.0.js
no rank

38288
6
species

585529
no rank
node597.members.0.js
6

1404244
3
species

1404245
no rank
3
node599.members.0.js

1719
species
node600.members.0.js
10

3
575200
species

no rank
3
node602.members.0.js
1224163

species
node603.members.0.js
6
38301

1230998
4
species

1437875
no rank
4
node605.members.0.js

156976
node606.members.0.js
2
species

35757
species
4
node607.members.0.js

14
152794
species

196164
14
node609.members.0.js
no rank

species
2
node610.members.0.js
191610

node611.members.0.js
3
species
1487956

136857
node612.members.0.js
1
species

1231000
3
species

node614.members.0.js
3
no rank
1408189

3
node615.members.0.js
species
702967

1727
1
species

no rank
1
node617.members.0.js
858619

38289
node618.members.0.js
5
2
species

306537
node619.members.0.js
3
no rank

species
3
225326

1121362
no rank
3
node621.members.0.js

node622.members.0.js
109
species
1652495

species
160386
4

1285583
node624.members.0.js
4
no rank

species
72366
78246
node625.members.0.js
1718

no rank
11
node626.members.0.js
1232383

no rank
node627.members.0.js
1014
1079988

340322
no rank
node628.members.0.js
4194

node629.members.0.js
9
no rank
1310161

node630.members.0.js
17
no rank
1232384

196627
no rank
226
496
node631.members.0.js

270
node632.members.0.js
no rank
1204414

1232381
no rank
node633.members.0.js
139

species
53
1408191

931089
node635.members.0.js
53
no rank

2
108486
species

2
node637.members.0.js
no rank
1451189

161896
species
node638.members.0.js
5

2
42817
species

1348662
2
node640.members.0.js
no rank

571915
species
1
node641.members.0.js

species
92706
17

1232385
no rank
17
node643.members.0.js

3
203263
species

3
node645.members.0.js
no rank
1431546

family
2
85025

1827
2
genus

1
node648.members.0.js
species
1805827

node649.members.0.js
1
species
1990687

order
1643682
2

family
85030
2

genus
88138
1

1
node653.members.0.js
species
477641

38501
1
genus

species
138336
1

no rank
node656.members.0.js
1
1146883

2
85008
order

family
2
28056

168694
1
genus

168697
1
species

391037
node661.members.0.js
1
no rank

genus
1
1873

node663.members.0.js
1
species
291594

order
1
622452

family
83778
1

genus
1
33981

species
131568
1

266940
1
node668.members.0.js
no rank

order
85006
389

family
85023
1

33877
1
genus

2080742
node672.members.0.js
1
species

1268
388
family

1269
386
genus

1270
352
species
node675.members.0.js
386

465515
no rank
node676.members.0.js
34

1663
2
node677.members.0.js
genus
1

656366
1
node678.members.0.js
species

1
2037
order

2049
1
family

1
1654
genus

species
1
node682.members.0.js
1912795

order
85011
7

2062
7
node684.members.0.js
family
1

6
1883
genus

species
node686.members.0.js
1
1885

2094021
species
node687.members.0.js
1

1950
1
species

subspecies
1
55158

316280
no rank
1
node690.members.0.js

1
node691.members.0.js
species
67267

species
node692.members.0.js
1
1535768

1882757
species
node693.members.0.js
1

order
85010
2

2
2070
family

2
1813
genus

129921
1
node697.members.0.js
species

1804986
species
1
node698.members.0.js

84998
2
class

order
2
1643822

2
1643826
family

genus
84111
1

84112
node703.members.0.js
1
species

genus
1
644652

1335613
1
node705.members.0.js
species

phylum
6
61221
node706.members.0.js
1239

node707.members.0.js
61209
3
class
91061

node708.members.0.js
61202
9
order
1385

186822
10
family

44249
node710.members.0.js
10
1
genus

189426
species
node711.members.0.js
9

7
90964
family

1279
7
genus

node714.members.0.js
1
species
1282

species
node715.members.0.js
6
29385

family
5
186818

1372
5
genus

species
1
161360

no rank
node719.members.0.js
1
1185653

4
node720.members.0.js
species
2058136

family
1
186820

genus
1
1637

species
1
node723.members.0.js
1639

186817
family
2
61170
node724.members.0.js

84406
1
genus

1
node726.members.0.js
species
2017483

19902
genus
node727.members.0.js
61164
1386

9
node728.members.0.js
species
35841

2093834
node729.members.0.js
5
species

5
node730.members.0.js
species
2011012

86661
54
species group
node731.members.0.js
387

51
node732.members.0.js
species
40
1428

no rank
1
29337

930170
no rank
node734.members.0.js
1

1417985
node735.members.0.js
1
no rank

node736.members.0.js
1
no rank
1423143

180850
1
node737.members.0.js
no rank

node738.members.0.js
6
no rank
1195464

no rank
1
node739.members.0.js
412694

1405
node740.members.0.js
2
species

31
species
node741.members.0.js
222
1396

1454382
9
node742.members.0.js
no rank

no rank
3
node743.members.0.js
361100

1217984
no rank
1
node744.members.0.js

526986
1
node745.members.0.js
no rank

1003239
167
node746.members.0.js
no rank

222523
2
node747.members.0.js
no rank

no rank
node748.members.0.js
8
288681

species
1
658666

node750.members.0.js
1
no rank
1330043

node751.members.0.js
3
species
1890302

1392
31
species
node752.members.0.js
43

no rank
node753.members.0.js
3
1449979

1
node754.members.0.js
no rank
673518

198094
node755.members.0.js
1
no rank

no rank
7
node756.members.0.js
261591

species
node757.members.0.js
6
580165

node758.members.0.js
5
4
species
64104

node759.members.0.js
1
no rank
527000

species
20
21
node760.members.0.js
1408

1
node761.members.0.js
no rank
315750

5
1404
species

node763.members.0.js
1
no rank
1006007

node764.members.0.js
4
no rank
1348623

666686
species
3
node765.members.0.js

79880
node766.members.0.js
8
species

859143
species
node767.members.0.js
3

79883
10
node768.members.0.js
species

33932
5
node769.members.0.js
species

species
5
86665

no rank
5
node771.members.0.js
272558

1479
species
5
node772.members.0.js

1446792
node773.members.0.js
1
species

1178537
species
10
node774.members.0.js

7
node775.members.0.js
species
352858

5
node776.members.0.js
species
632773

129985
node777.members.0.js
6
species

13
node778.members.0.js
species
1402861

species
node779.members.0.js
3
2026248

1441095
22
node780.members.0.js
species

species
9
node781.members.0.js
86664

756828
node782.members.0.js
1
species

species
1471
3

no rank
node784.members.0.js
3
796606

1705566
species
node785.members.0.js
5

2049935
node786.members.0.js
10
species

1547283
species
6
node787.members.0.js

species
22
node788.members.0.js
561879

node789.members.0.js
6
species
1565991

561
node790.members.0.js
species
1664069

species
node791.members.0.js
3
264697

1467
species
node792.members.0.js
1

324767
11
species

11
node794.members.0.js
no rank
1367477

2267265
node795.members.0.js
1
species

species
3
4
node796.members.0.js
1478

1349754
1
node797.members.0.js
no rank

7
79885
species

398511
no rank
node799.members.0.js
7

19
node800.members.0.js
species
15
1398

node801.members.0.js
2
no rank
1121088

2
node802.members.0.js
no rank
345219

199441
species
node803.members.0.js
8

1837130
9
node804.members.0.js
species

species
1413
2

2
node806.members.0.js
no rank
649639

1792192
3
species group
node807.members.0.js
12

1049581
6
node808.members.0.js
species

293387
species
node809.members.0.js
3

421767
1
node810.members.0.js
species

653685
node811.members.0.js
39922
2261
species group

species
5629
5932
node812.members.0.js
1648923

no rank
303
node813.members.0.js
766760

species
8
14
node814.members.0.js
1452

no rank
node815.members.0.js
5
1239783

node816.members.0.js
1
subspecies
1529886

119858
species
node817.members.0.js
588

species subgroup
15
302
node818.members.0.js
1938374

node819.members.0.js
259
245
species
492670

no rank
1
node820.members.0.js
1338518

1225788
no rank
1
node821.members.0.js

no rank
node822.members.0.js
12
1458206

1390
28
node823.members.0.js
species
21

no rank
3
node824.members.0.js
1034836

node825.members.0.js
2
no rank
692420

1126211
2
node826.members.0.js
no rank

18076
species
node827.members.0.js
30597
1402

279010
no rank
node828.members.0.js
12376

node829.members.0.js
145
no rank
1126218

1423
140
species
node830.members.0.js
228

483913
subspecies
node831.members.0.js
21

subspecies
14
96241

655816
9
node833.members.0.js
no rank

1052585
node834.members.0.js
5
no rank

936156
no rank
6
node835.members.0.js

86029
subspecies
node836.members.0.js
11

1496303
1
node837.members.0.js
subspecies

subspecies
13
35
node838.members.0.js
135461

no rank
1
node839.members.0.js
1192196

1404258
no rank
13
node840.members.0.js

1052588
node841.members.0.js
4
no rank

1302650
4
node842.members.0.js
no rank

species
4
665099

1196031
node844.members.0.js
4
no rank

91
node845.members.0.js
species
1856406

1581038
species
3
node846.members.0.js

species
300825
3

3
node848.members.0.js
no rank
1246626

1
node849.members.0.js
genus
1329200

genus
1
182709

746691
1
species

1238184
node852.members.0.js
1
no rank

1
129337
genus

1505648
1
species group

33938
1
node855.members.0.js
species

order
186826
4

1
1300
family

genus
1
1301

1345
species
node859.members.0.js
1

family
81852
1

genus
1
1350

species
node862.members.0.js
1
2005703

family
33958
1

genus
1
1253

1
1670797
no rank

node866.members.0.js
1
species
1254

family
186828
1

1
2747
genus

2751
species
node869.members.0.js
1

6
186801
class

186802
5
order

4
186807
family

genus
1562
1

1
node874.members.0.js
species
1833852

471826
1
genus

species
1
471827

477974
no rank
node877.members.0.js
1

2
36853
genus

species
36854
1

756499
no rank
node880.members.0.js
1

species
1
node881.members.0.js
49338

186803
1
family

genus
1
1506553

1871021
species
node884.members.0.js
1

order
68295
1

1
543371
family

44000
node887.members.0.js
1
genus

phylum
544448
1

class
1
31969

order
2085
1

1
2092
family

genus
1
2093

2112
species
node893.members.0.js
1

no rank
1798711
4

4
1117
phylum

order
1890424
2

1213
2
family

1218
2
node898.members.0.js
genus
1

species
1219
1

no rank
node900.members.0.js
1
74547

1161
1
order

family
1
1162

genus
1
1163

1165
1
species

no rank
1
node905.members.0.js
272123

subclass
1
1301283

1150
1
order

1
1892255
family

1
241421
genus

species
241425
1

no rank
node911.members.0.js
1
1173022

no rank
13
1783270

no rank
13
68336

phylum
976
13

class
5
768503

order
768507
5

family
89373
3

genus
1
861914

1834519
species
node919.members.0.js
1

genus
105
2

2259595
species
node921.members.0.js
1

1
106
species

761193
node923.members.0.js
1
no rank

1853232
2
family

genus
1
2
node925.members.0.js
89966

species
node926.members.0.js
1
1385663

class
1853228
2

order
1853229
2

2
563835
family

1
398041
genus

1492898
1
node931.members.0.js
species

genus
649460
1

1
node933.members.0.js
species
477680

1
1937959
class

1
1936988
order

family
1
1937961

genus
2349
1

species
1
2350

node939.members.0.js
1
no rank
760192

class
3
117743

3
200644
order

family
49546
3

2
52959
genus

1312072
species
2
node944.members.0.js

286104
1
genus

node946.members.0.js
1
species
1936080

class
117747
2

2
200666
order

family
84566
2

genus
423349
1

node951.members.0.js
1
species
1550579

genus
1
node952.members.0.js
28453
