# Supplementary material for: Benchmarking the MinION: Evaluating long reads for microbial profiling
Source: Sci Rep. 2020 Mar 20;10:5125. doi: 10.1038/s41598-020-61989-x (PMC7083898; doi:10.1038/s41598-020-61989-x)
Supplement: Supplementary file 2 — Supplementary information2. [file 41598_2020_61989_MOESM2_ESM.zip › sample_barcode_3/centrifuge.html]

Javascript must be enabled to view this page.

members
magnitude
magnitudeUnassigned
count
unassigned
taxon
rank

BC3\_k1\_centrifuge\_results

node0.members.0.js
109949
1

5
superkingdom
2157

28890
phylum
5

183968
class
1

1
order
2258

1
family
2259

1
genus
2260

1008460
species
node7.members.0.js
1

3
no rank
2290931

224756
2
class

2191
1
order

1
family
1198451

395331
1
genus

species
358766
1
node13.members.0.js

1
order
94695

2206
1
family

1
genus
2207

1
node17.members.0.js
species
2208

1
class
183963

2235
1
order

1963268
1
family

genus
1
146825

1
node22.members.0.js
146826
species

no rank
1
2283794

183939
class
1

order
1
2182

1
family
196117

genus
1
196119

1
node28.members.0.js
2189
species

superkingdom
2
node29.members.0.js
109477
10

1224
phylum
node30.members.0.js
77112
41

subphylum
1
68525

1
class
28221

1
order
213115

family
1
194924

1
genus
872

881
species
node36.members.0.js
1

28216
class
node37.members.0.js
23755
1

206389
1
order

2008794
1
family

1
node40.members.0.js
genus
12960

206351
11295
order

1499392
11294
family

11294
no rank
90153

535
genus
node44.members.0.js
11294
16

11259
node45.members.0.js
species
536

14
node46.members.0.js
species
1108595

species
2059672
node47.members.0.js
5

481
1
family

1
genus
538

539
species
1
node50.members.0.js

80840
order
11
12457
node51.members.0.js

no rank
1
119065

224471
1
no rank

genus
1
28067

node55.members.0.js
1
28068
species

family
506
node56.members.0.js
12425
5

genus
222
31
12408
node57.members.0.js

32002
species
30
node58.members.0.js

217203
species
1
node59.members.0.js

1758194
species
2
node60.members.0.js

species
217204
3
node61.members.0.js

12341
node62.members.0.js
species
85698

517
genus
node63.members.0.js
10
2

1
node64.members.0.js
463040
species

species
521
1
node65.members.0.js

1416806
species
1
node66.members.0.js

species
94624
5
node67.members.0.js

genus
2
90243

species
90245
node69.members.0.js
2

3
family
75682

1
genus
202907

1
node72.members.0.js
species
279058

149698
genus
2

node74.members.0.js
1
species
1707785

node75.members.0.js
1
1678028
species

119060
family
11

4
genus
106589

species
119219
2
node78.members.0.js

node79.members.0.js
1
106590
species

1
node80.members.0.js
species
248026

genus
5
32008

species group
2
111527

species
57975
1
node83.members.0.js

28450
species
1
node84.members.0.js

87882
2
species group

1
node86.members.0.js
species
292

node87.members.0.js
1
95486
species

species
640511
node88.members.0.js
1

48736
2
genus

329
species
node90.members.0.js
1

1
node91.members.0.js
species
305

node92.members.0.js
6
1
family
80864

1649468
2
genus

2
node94.members.0.js
species
2109913

genus
1
12916

1
node96.members.0.js
species
232721

219181
1
genus

1658672
species
node98.members.0.js
1

174951
genus
1

node100.members.0.js
1
species
94132

order
1
32003

206379
1
family

1
genus
914

node104.members.0.js
1
44574
species

16
class
28211

order
1
204455

1
family
31989

1
genus
299261

1
node109.members.0.js
299262
species

1921002
1
order

2100208
1
family

1509243
genus
1

1509244
species
node113.members.0.js
1

356
order
10

41294
family
2

1
genus
374

node117.members.0.js
1
species
1355477

85413
genus
1

1526658
species
node119.members.0.js
1

82115
3
family

1
genus
323620

node122.members.0.js
1
879274
species

no rank
2
227290

genus
1
1525371

node125.members.0.js
1
species
399

379
genus
1

species
384
node127.members.0.js
1

1
family
119045

2282523
genus
1

1
node130.members.0.js
223967
species

family
1
335928

genus
1
279

node133.members.0.js
1
280
species

45401
family
3

3
genus
46913

species
2083786
1
node136.members.0.js

species
1736675
node137.members.0.js
2

order
2
204441

2
family
41295

171436
genus
1

171437
species
1
node141.members.0.js

191
genus
1

193
species
1
node143.members.0.js

order
2
204457

1
family
41297

genus
1
1434046

node147.members.0.js
1
266812
species

335929
1
family

1
genus
1041

1
node150.members.0.js
502682
species

1236
class
10
node151.members.0.js
53299

order
19
72274

family
19
135621

286
genus
node154.members.0.js
19
1

237610
species
1
node155.members.0.js

136843
species group
4

3
node157.members.0.js
species
294

1
node158.members.0.js
200451
species

1788301
species
node159.members.0.js
1

136842
1
species group

species
587753
1
node161.members.0.js

species group
10
136841

species
300
1
node163.members.0.js

species
287
9
node164.members.0.js

136846
1
species group

species subgroup
1
578833

316
species
1
node167.members.0.js

135624
order
1

family
1
84642

642
1
genus

species
651
1
node171.members.0.js

1
order
72273

135616
1
family

genus
1
40222

1
node175.members.0.js
species
754477

135622
order
3

72275
1
family

genus
1
226

1
node179.members.0.js
715451
species

267890
family
1

genus
1
22

species
56812
1
node182.members.0.js

1
family
267888

53246
1
genus

1
node185.members.0.js
43662
species

135623
1
order

family
1
641

662
1
genus

1
species group
717610

680
species
1
node190.members.0.js

8223
order
135614

32033
family
node192.members.0.js
8223
2

genus
338
8217
node193.members.0.js
19

node194.members.0.js
1
species
1985254

node195.members.0.js
8188
species
339

90270
species
2
node196.members.0.js

643453
species group
1

node198.members.0.js
1
species
346

species
442694
2
node199.members.0.js

species
29447
1
node200.members.0.js

3
node201.members.0.js
56454
species

genus
40323
1
4
node202.members.0.js

species group
2
995085

species
40324
node204.members.0.js
2

1827305
species
1
node205.members.0.js

135618
1
order

403
1
family

1
genus
39773

271065
species
1
node209.members.0.js

15
node210.members.0.js
45036
91347
order

family
543
26
11467
node211.members.0.js

5
genus
590

5
node213.members.0.js
species
28901

genus
570
node214.members.0.js
8
1

2
node215.members.0.js
species
548

1
node216.members.0.js
species
1905288

4
node217.members.0.js
573
species

genus
2
160674

54291
species
node219.members.0.js
1

species
575
node220.members.0.js
1

1330545
genus
1

1
node222.members.0.js
1907578
species

1
node223.members.0.js
20
561
genus

19
node224.members.0.js
species
562

genus
547
node225.members.0.js
8782
476

1
node226.members.0.js
species
1692238

2051905
species
58
node227.members.0.js

node228.members.0.js
1
1914861
species

354276
species group
8246

species
1812935
1
node230.members.0.js

node231.members.0.js
1
species
208224

species
299767
node232.members.0.js
1

5680
5691
node233.members.0.js
species
158836

node234.members.0.js
7
subspecies
1296536

node235.members.0.js
4
subspecies
1812934

550
species
node236.members.0.js
2550

2027919
species
1
node237.members.0.js

node238.members.0.js
1
61645
species

23
2612
node239.members.0.js
413496
genus

node240.members.0.js
1
species
535744

413502
species
3
node241.members.0.js

species
28141
node242.members.0.js
2565

node243.members.0.js
20
species
413503

158483
1
genus

1
node245.members.0.js
158822
species

3
genus
1330547

2
node247.members.0.js
283686
species

node248.members.0.js
1
1158459
species

genus
83654
1
2
node249.members.0.js

species
83655
1
node250.members.0.js

4
genus
544

species
67825
1
node252.members.0.js

1344959
3
species group

57706
species
node254.members.0.js
2

node255.members.0.js
1
species
67827

no rank
1
191675

84563
no rank
1

1906660
genus
1

1778264
species
1
node259.members.0.js

node260.members.0.js
9531
2
family
1903411

613
genus
3
9526
node261.members.0.js

node262.members.0.js
1
28151
species

2033438
species
node263.members.0.js
1

node264.members.0.js
1
species
614

node265.members.0.js
9508
47917
species

1
node266.members.0.js
species
104623

species
82996
node267.members.0.js
1

species
615
10
node268.members.0.js

1
node269.members.0.js
3
genus
629

species
29484
1
node270.members.0.js

species group
1
1649845

632
species
node272.members.0.js
1

family
1903410
2
24005
node273.members.0.js

122277
3
genus

species
1905730
1
node275.members.0.js

node276.members.0.js
2
29471
species

71655
1
genus

1
node278.members.0.js
1109412
species

103
node279.members.0.js
23999
genus
204037

species
1089444
23758
node280.members.0.js

7
node281.members.0.js
species
204042

node282.members.0.js
34
1778540
species

species
204039
20
node283.members.0.js

204038
species
node284.members.0.js
77

1903409
8
family

1
genus
2100764

665913
species
node287.members.0.js
1

551
genus
1
3
node288.members.0.js

2
node289.members.0.js
species
215689

53335
4
genus

node291.members.0.js
2
592316
species

species
1484157
1
node292.members.0.js

species
470934
node293.members.0.js
1

1903412
9
family

8
node295.members.0.js
1
635
genus

7
node296.members.0.js
species
67780

568
genus
node297.members.0.js
1

1
family
1903414

626
genus
1

1
node300.members.0.js
species
351671

no rank
1
118884

33811
no rank
1

1248727
species
node303.members.0.js
1

order
1
1706369

1706375
1
family

1434050
genus
1

species
1470434
node307.members.0.js
1

order
1
1934945

1934946
family
1

1934947
1
genus

species
1810504
node311.members.0.js
1

1
order
135613

1
family
72276

1051
1
genus

node315.members.0.js
1
species
1442136

1783272
32348
no rank

1
phylum
544448

class
1
31969

2085
order
1

2092
1
family

1
genus
2093

species
2118
node322.members.0.js
1

201174
phylum
10192

1
class
84998

order
1
84999

1643824
family
1

1
genus
133925

133926
species
1
node328.members.0.js

class
1760
3
node329.members.0.js
10191

85011
6
order

6
family
2062

1883
genus
5

1
node333.members.0.js
1912
species

node334.members.0.js
1
species
42684

species
1940
node335.members.0.js
1

47763
species
node336.members.0.js
1

species
1169025
1
node337.members.0.js

genus
1
2063

1
node339.members.0.js
68173
species

order
3
85008

28056
3
family

1
genus
673534

1
node343.members.0.js
2024580
species

1865
1
genus

node345.members.0.js
1
649831
species

84593
1
genus

1
node347.members.0.js
species
1003110

6389
order
85007

85025
family
3

genus
3
1817

species
37326
2
node351.members.0.js

node352.members.0.js
1
species
37329

6383
family
1653

1716
genus
6383
node354.members.0.js
175

species
1718
6166
node355.members.0.js

species
28028
1
node356.members.0.js

node357.members.0.js
38
species
92706

1
node358.members.0.js
species
38289

146827
species
node359.members.0.js
1

1
node360.members.0.js
species
1652495

1762
family
3

1073531
1
genus

1788
species
node363.members.0.js
1

1
node364.members.0.js
2
genus
1763

1
node365.members.0.js
1781
species

85006
3782
order

3782
family
1268

genus
1
1663

node369.members.0.js
1
290399
species

genus
3781
1269

3781
node371.members.0.js
1270
species

85010
6
order

2070
family
6

43356
1
genus

node375.members.0.js
1
43357
species

1
genus
142577

species
530584
node377.members.0.js
1

1
genus
1847

1690815
species
node379.members.0.js
1

genus
1
2029

node381.members.0.js
1
860235
species

1813
genus
1

1
node383.members.0.js
208439
species

165301
1
genus

species
1586287
node385.members.0.js
1

1
order
85009

family
1
31957

1912216
genus
1

1
node389.members.0.js
species
1747

2037
1
order

1
family
2049

genus
1
1654

1
node393.members.0.js
species
2081702

1
no rank
1798711

1117
phylum
node395.members.0.js
1

200795
1
phylum

class
1
301297

1202465
1
order

1202464
family
1

61434
genus
1

node401.members.0.js
1
61435
species

1239
22153
phylum

class
1
909932

909929
1
order

1
family
1843490

365348
1
genus

484770
species
1
node407.members.0.js

186801
class
3

order
3
186802

2
family
186803

830
genus
1

43305
species
1
node412.members.0.js

698776
genus
1

1
node414.members.0.js
species
29360

31979
1
family

1
genus
1981033

node417.members.0.js
1
2086584
species

22149
class
91061

186826
order
6

33958
family
2

1578
genus
2

species
2099788
1
node422.members.0.js

1612
species
node423.members.0.js
1

1300
family
1

1
genus
1301

1
node426.members.0.js
197614
species

family
1
81850

1243
1
genus

species
1245
1
node429.members.0.js

186827
family
2

1
2
node431.members.0.js
1375
genus

1376
species
1
node432.members.0.js

order
1385
4
node433.members.0.js
22143

186818
4
family

1372
genus
4

node436.members.0.js
4
species
2058136

family
4561
90964

node438.members.0.js
4561
14
genus
1279

246432
species
node439.members.0.js
2

node440.members.0.js
1
61015
species

species
1288
node441.members.0.js
2

species
29382
node442.members.0.js
3

5
node443.members.0.js
species
1280

70258
species
node444.members.0.js
1

node445.members.0.js
3
species
214473

1715860
species
6
node446.members.0.js

4523
node447.members.0.js
species
29385

1
node448.members.0.js
species
29384

186822
10801
family

node450.members.0.js
10801
6
44249
genus

1536775
species
node451.members.0.js
1

species
189426
node452.members.0.js
10793

1
node453.members.0.js
61624
species

186820
1
family

1637
genus
1

node456.members.0.js
1
1639
species

family
6772
186817

genus
1
351195

1230341
species
node459.members.0.js
1

node460.members.0.js
6771
3162
genus
1386

species group
3531
653685

2
node462.members.0.js
1452
species

4
species subgroup
1938374

node464.members.0.js
1
1390
species

492670
species
node465.members.0.js
3

species
119858
node466.members.0.js
4

1423
species
node467.members.0.js
1

node468.members.0.js
3439
1402
species

species
1648923
node469.members.0.js
81

1
node470.members.0.js
species
1837130

node471.members.0.js
3
1664069
species

2026248
species
node472.members.0.js
28

node473.members.0.js
1
1408
species

species group
6
86661

1396
species
5
node475.members.0.js

node476.members.0.js
1
species
1392

1856406
species
node477.members.0.js
38

node478.members.0.js
1
79883
species

1783257
no rank
3

203682
3
phylum

class
3
203683

order
3
112

1763524
2
family

127
1
genus

species
128
node485.members.0.js
1

genus
1
466152

node487.members.0.js
1
466153
species

family
1
126

1
genus
1936111

1891926
species
1
node490.members.0.js

no rank
4
1783270

4
no rank
68336

976
4
phylum

class
2
768503

order
2
768507

1853232
family
2

genus
2
89966

1411621
species
1
node498.members.0.js

node499.members.0.js
1
species
1446467

200643
1
class

171549
order
1

171552
family
1

1
genus
838

1
node504.members.0.js
species
28132

class
1
117743

200644
order
1

49546
family
1

1
genus
237

node509.members.0.js
1
species
996

node510.members.0.js
466
