# Supplementary material for: Benchmarking the MinION: Evaluating long reads for microbial profiling
Source: Sci Rep. 2020 Mar 20;10:5125. doi: 10.1038/s41598-020-61989-x (PMC7083898; doi:10.1038/s41598-020-61989-x)
Supplement: Supplementary file 2 — Supplementary information2. [file 41598_2020_61989_MOESM2_ESM.zip › sample_barcode_3/kraken.html]

Javascript must be enabled to view this page.

members
magnitude
magnitudeUnassigned
count
unassigned
taxon
rank

BC3\_kraken\_krona

9
node0.members.0.js
109948

node1.members.0.js
2777

superkingdom
node2.members.0.js
10
107162
2

75333
1224
phylum
node3.members.0.js
64

class
node4.members.0.js
16
51981
1236

135614
8069
order

2
node6.members.0.js
family
32033
8069

338
8066
60
node7.members.0.js
genus

8005
339
species
752
node8.members.0.js

no rank
node9.members.0.js
7
17
359385

no rank
node10.members.0.js
10
990315

no rank
node11.members.0.js
6394
7224
340

24
314565
no rank
node12.members.0.js

13
1281282
no rank
node13.members.0.js

3
1357999
no rank
node14.members.0.js

node15.members.0.js
no rank
190485
790

no rank
node16.members.0.js
12
92826

species
node17.members.0.js
1
56460

40323
1
node18.members.0.js
genus

72274
13
order

135621
13
family

node21.members.0.js
1
genus
286
13

136841
11
species group

10
287
species
node23.members.0.js

species subgroup
1
1232139

node25.members.0.js
species
330
1

node26.members.0.js
species
237609
1

order
83
node27.members.0.js
43883
91347

family
node28.members.0.js
22
23114
1903410

genus
2
122277

2
species
554

node31.members.0.js
subspecies
180957
1

subspecies
1
555

no rank
node33.members.0.js
1
1218933

23090
204037
genus
1143
node34.members.0.js

species
node35.members.0.js
21364
21877
1089444

node36.members.0.js
no rank
1225786
378

1224151
65
node37.members.0.js
no rank

49
1226344
no rank
node38.members.0.js

21
1224152
no rank
node39.members.0.js

204039
10
7
node40.members.0.js
species

3
1223570
no rank
node41.members.0.js

556
11
1
node42.members.0.js
species

node43.members.0.js
no rank
1223571
4

1223569
6
node44.members.0.js
no rank

species
node45.members.0.js
9
568766

204042
4
node46.members.0.js
3
species

1223567
1
node47.members.0.js
no rank

species
1
69223

no rank
node49.members.0.js
1
1224150

4
1224145
species
node50.members.0.js

node51.members.0.js
11
species
204038
31

node52.members.0.js
no rank
1223572
6

6
1224149
no rank
node53.members.0.js

node54.members.0.js
no rank
198628
1

subspecies
7
204040

node56.members.0.js
no rank
1223574
7

1903409
1
family

genus
1
53335

node59.members.0.js
species
592316
1

1903412
family
4

635
genus
4

node62.members.0.js
species
67780
4

1903411
9396
node63.members.0.js
2
family

node64.members.0.js
3
genus
613
9392

node65.members.0.js
species
47917
9386

species
node66.members.0.js
1
615

species
2
82996

2
1006598
no rank
node68.members.0.js

629
genus
2

1649845
species group
1

species
node71.members.0.js
1
632

28152
1
node72.members.0.js
species

node73.members.0.js
69
family
543
11285

160674
1
genus

1
54291
species
node75.members.0.js

genus
18
node76.members.0.js
8624
547

4518
node77.members.0.js
species group
354276
8605

species
node78.members.0.js
5
1812935

208224
2
node79.members.0.js
species

species
node80.members.0.js
3
1915310

node81.members.0.js
139
species
158836
3400

subspecies
node82.members.0.js
36
1812934

231
301105
subspecies
node83.members.0.js

subspecies
node84.members.0.js
28
1296536

node85.members.0.js
subspecies
301102
168

subspecies
node86.members.0.js
2798
299766

61645
98
34
node87.members.0.js
species

49
640513
no rank
node88.members.0.js

15
1421338
no rank
node89.members.0.js

299767
6
node90.members.0.js
species

550
573
node91.members.0.js
503
species

19
subspecies
336306

3
1211025
no rank
node93.members.0.js

node94.members.0.js
no rank
716541
16

69219
subspecies
41

node96.members.0.js
no rank
1104326
41

6
1354030
no rank
node97.members.0.js

1045856
4
node98.members.0.js
no rank

node99.members.0.js
species
399742
1

1
1330547
genus
node100.members.0.js

16
genus
1330546

16
1334193
species
node102.members.0.js
14

701347
2
node103.members.0.js
no rank

node104.members.0.js
84
genus
413496
2546

28141
2461
node105.members.0.js
2199
species

node106.members.0.js
no rank
1138308
133

956149
10
node107.members.0.js
no rank

no rank
node108.members.0.js
119
290339

413503
species
1

no rank
node110.members.0.js
1
1159491

7
genus
570

species
node112.members.0.js
3
573

2
244366
species
node113.members.0.js
1

no rank
node114.members.0.js
1
640131

species
node115.members.0.js
1
1905288

node116.members.0.js
species
1463165
1

2
544
genus
1
node117.members.0.js

node118.members.0.js
species
67824
1

genus
4
590

node120.members.0.js
1
species
28901
4

3
subspecies
59201

2
90370
no rank
node122.members.0.js

no rank
1
611

1160717
1
node124.members.0.js
no rank

genus
15
561

562
15
8
node126.members.0.js
species

941322
1
node127.members.0.js
no rank

node128.members.0.js
no rank
585397
2

3
405955
no rank
node129.members.0.js

1
no rank
83334

node131.members.0.js
no rank
1328859
1

5
node132.members.0.js
class
28216
23288

9
node133.members.0.js
order
80840
12220

506
12206
16
node134.members.0.js
family

genus
1
305976

node136.members.0.js
species
1007105
1

12188
222
genus
159
node137.members.0.js

1881016
1
node138.members.0.js
species

species
node139.members.0.js
1
217204

10188
node140.members.0.js
species
85698
12026

1167634
980
node141.members.0.js
no rank

151
762376
no rank
node142.members.0.js

707
562971
no rank
node143.members.0.js

32002
1
node144.members.0.js
species

genus
node145.members.0.js
1
290425

119065
2
no rank

no rank
2
80841

species
node148.members.0.js
2
1469502

119060
family
3

genus
1
106589

1796606
1
node151.members.0.js
species

32008
2
genus

87882
2
species group

1
species
87883

1
985079
no rank
node155.members.0.js

species
node156.members.0.js
1
95486

order
1
node157.members.0.js
11063
206351

family
node158.members.0.js
8
11062
1499392

90153
no rank
11054

genus
40
node160.members.0.js
11054
535

1
1108595
species
node161.members.0.js

species
11013
536

no rank
node163.members.0.js
11013
243365

1783272
no rank
31819

10030
phylum
201174

node166.members.0.js
3
class
1760
10030

85006
3710
node167.members.0.js
1
order

3709
1268
family
1
node168.members.0.js

1269
genus
3708

1270
3708
3359
node170.members.0.js
species

no rank
node171.members.0.js
349
465515

1
order
85009

31957
1
family

1
genus
1912216

species
node175.members.0.js
1
1747

6316
order
85007

family
6316
1653

genus
node178.members.0.js
518
6316
1716

1705
1
node179.members.0.js
species

node180.members.0.js
species
1652495
1

5794
1718
species
5536
node181.members.0.js

1079988
37
node182.members.0.js
no rank

9
1310161
no rank
node183.members.0.js

85
196627
no rank
node184.members.0.js
74

no rank
node185.members.0.js
11
1204414

127
340322
no rank
node186.members.0.js

1
43771
species
node187.members.0.js

1408191
species
1

931089
1
node189.members.0.js
no rank

21789
1239
phylum
1
node190.members.0.js

21788
91061
class
node191.members.0.js
2

1385
21786
node192.members.0.js
3
order

6650
186817
family
node193.members.0.js
1

6649
1386
genus
node194.members.0.js
2994

species
node195.members.0.js
1
1565991

species
node196.members.0.js
1
135735

1
1408
species
node197.members.0.js

species
node198.members.0.js
3
756828

561879
1
node199.members.0.js
species

653685
3588
node200.members.0.js
321
species group

25
119858
species
node201.members.0.js

2950
1402
species
node202.members.0.js
1781

no rank
node203.members.0.js
5
1126218

1164
279010
no rank
node204.members.0.js

8
node205.members.0.js
species
1423
13

86029
1
node206.members.0.js
subspecies

96241
1
subspecies

no rank
node208.members.0.js
1
655816

135461
3
2
node209.members.0.js
subspecies

1404258
1
node210.members.0.js
no rank

node211.members.0.js
species
1452
1

species
257
node212.members.0.js
269
1648923

12
766760
no rank
node213.members.0.js

1938374
9
node214.members.0.js
1
species subgroup

species
2
1390

no rank
node216.members.0.js
2
1034836

6
492670
species
4
node217.members.0.js

1458206
1
node218.members.0.js
no rank

1
1338518
no rank
node219.members.0.js

species group
3
node220.members.0.js
34
86661

1428
3
node221.members.0.js
1
species

29339
no rank
1

1279365
1
node223.members.0.js
no rank

no rank
1
29337

1
930170
no rank
node225.members.0.js

species
18
node226.members.0.js
23
1396

1217984
1
node227.members.0.js
no rank

451709
1
node228.members.0.js
no rank

3
288681
no rank
node229.members.0.js

node230.members.0.js
2
species
1392
3

1
1392837
no rank
node231.members.0.js

1405
2
node232.members.0.js
species

79885
species
1

no rank
node234.members.0.js
1
398511

1837130
1
node235.members.0.js
species

1664069
18
node236.members.0.js
species

node237.members.0.js
species
1856406
6

family
10658
186822

node239.members.0.js
25
genus
44249
10658

1
species
159743

node241.members.0.js
no rank
985665
1

node242.members.0.js
species
189426
10631

species
node243.members.0.js
1
1566358

90964
4475
node244.members.0.js
1
family

4474
1279
genus
node245.members.0.js
70

species
node246.members.0.js
15
1288

node247.members.0.js
species
61015
1

species
node248.members.0.js
25
246432

70255
16
node249.members.0.js
species

1280
48
node250.members.0.js
species

node251.members.0.js
species
1715860
2

1637
node252.members.0.js
species
29385
4288

147452
2651
subspecies

no rank
node254.members.0.js
2651
342451

45972
species
3

3
1276282
no rank
node256.members.0.js

species
node257.members.0.js
1
1281

species
node258.members.0.js
5
214473
