# Supplementary material for: Benchmarking the MinION: Evaluating long reads for microbial profiling
Source: Sci Rep. 2020 Mar 20;10:5125. doi: 10.1038/s41598-020-61989-x (PMC7083898; doi:10.1038/s41598-020-61989-x)
Supplement: Supplementary file 2 — Supplementary information2. [file 41598_2020_61989_MOESM2_ESM.zip › sample_barcode_3/kraken2.html]

Javascript must be enabled to view this page.

members
magnitude
magnitudeUnassigned
count
unassigned
taxon
rank

BC3\_kraken2\_krona

109948
node0.members.0.js
1

1297
node1.members.0.js

superkingdom
18
2
node2.members.0.js
108649

32148
1783272
no rank

phylum
22006
node4.members.0.js
1239
2

class
909932
2

order
1
1843489

family
31977
1

genus
1
39948

species
node9.members.0.js
2161821
1

order
1
909929

1
1843490
family

genus
1
365348

species
365349
1

1192197
node14.members.0.js
1
no rank

class
1
22002
91061
node15.members.0.js

22000
1385
node16.members.0.js
3
order

186818
1
family

genus
1372
1

species
2058136
node19.members.0.js
1

family
4530
90964
node20.members.0.js
2

4528
node21.members.0.js
1279
49
genus

4450
4459
node22.members.0.js
29385
species

9
147452
subspecies

node24.members.0.js
342451
9
no rank

246432
node25.members.0.js
4
species

2
node26.members.0.js
61015
species

node27.members.0.js
214473
1
species

species
6
1280
node28.members.0.js
8

subspecies
46170
node29.members.0.js
2

1
node30.members.0.js
1283
species

species
2
1282
node31.members.0.js

2
1290
node32.members.0.js
species

family
186822
node33.members.0.js
10736
1

genus
10735
node34.members.0.js
44249
19

10712
node35.members.0.js
189426
species

species
1
node36.members.0.js
1532905

species
159743
1

985665
node38.members.0.js
1
no rank

1
1566358
node39.members.0.js
species

2044880
1
species group

species
1
483937

1
node42.members.0.js
1073571
no rank

family
6730
186817

genus
6730
1386
node44.members.0.js
2230

3
45
86661
node45.members.0.js
species group

1
node46.members.0.js
64104
species

25
1396
node47.members.0.js
3
species

no rank
1454382
node48.members.0.js
1

1003239
node49.members.0.js
21
no rank

8
node50.members.0.js
1392
species

species
1428
node51.members.0.js
8

262
4344
653685
node52.members.0.js
species group

species
1402
node53.members.0.js
3314
1930

node54.members.0.js
279010
1372
no rank

12
1126218
node55.members.0.js
no rank

node56.members.0.js
1423
29
15
species

96241
4
subspecies

4
655816
node58.members.0.js
no rank

subspecies
2
node59.members.0.js
86029

subspecies
483913
node60.members.0.js
3

2
936156
node61.members.0.js
no rank

3
135461
subspecies

no rank
2
1052588
node63.members.0.js

1
node64.members.0.js
1404258
no rank

species subgroup
1938374
node65.members.0.js
26
2

species
node66.members.0.js
492670
23
20

3
node67.members.0.js
1458206
no rank

species
node68.members.0.js
1390
1

species
2
1452
node69.members.0.js
1

1
node70.members.0.js
1239783
no rank

648
1648923
node71.members.0.js
617
species

31
node72.members.0.js
766760
no rank

species
63
node73.members.0.js
119858

species
1402861
node74.members.0.js
1

species
2
node75.members.0.js
199441

species
1
86664
node76.members.0.js

node77.members.0.js
561879
6
species

species
1467
node78.members.0.js
1

species
1
node79.members.0.js
1705566

species
2
1398
node80.members.0.js
3

no rank
node81.members.0.js
345219
1

species
node82.members.0.js
352858
1

species group
1792192
1

node84.members.0.js
1049581
1
species

species
2
1441095
node85.members.0.js

node86.members.0.js
1178537
1
species

8
node87.members.0.js
1856406
species

species
1581038
node88.members.0.js
1

324767
1
species

no rank
node90.members.0.js
1367477
1

1
1471
species

no rank
1
node92.members.0.js
796606

node93.members.0.js
1408
1
species

species
1
665099

no rank
node95.members.0.js
1196031
1

2
79880
node96.members.0.js
species

species
2
node97.members.0.js
35841

1
2093834
node98.members.0.js
species

2
node99.members.0.js
2049935
species

1
node100.members.0.js
859143
species

1
node101.members.0.js
421767
species

1664069
node102.members.0.js
69
species

order
186826
node103.members.0.js
1

phylum
1
544448

1
31969
class

2085
1
order

family
1
2092

genus
1
2093

species
28227
1

1
272633
node110.members.0.js
no rank

200795
1
phylum

475962
1
class

order
475963
1

family
475964
1

genus
233191
1

1
133453
species

no rank
926550
node117.members.0.js
1

phylum
1
node118.members.0.js
201174
10138

class
8
10136
1760
node119.members.0.js

85013
1
order

1
74712
family

genus
1
1434010

species
1907575
node123.members.0.js
1

5
85009
order

3
31957
family

genus
1
1912216

species
1
node127.members.0.js
1747

genus
1
1912215

species
node129.members.0.js
1748
1

genus
1743
1

node131.members.0.js
1744
1
species

2
85015
family

1839
1
genus

1
450734
species

1
node135.members.0.js
1300347
no rank

genus
86795
1

species
1
node137.members.0.js
642780

1
85008
order

28056
1
family

genus
1873
1

species
1
285665
node141.members.0.js

node142.members.0.js
85006
3750
2
order

85021
1
family

125287
1
genus

species
1
node145.members.0.js
2283195

1
3746
1268
node146.members.0.js
family

32207
1
genus

species
1
43675
node148.members.0.js

3744
1269
genus

species
3371
3744
node150.members.0.js
1270

no rank
373
465515
node151.members.0.js

1
125316
family

84756
1
genus

species
1
84757

no rank
471853
node155.members.0.js
1

6366
85007
order

85025
1
family

genus
1827
1

species
1
1564114
node159.members.0.js

6365
1653
family

genus
340
1716
node161.members.0.js
6365

node162.members.0.js
43771
1
species

species
node163.members.0.js
1697
1

1724
node164.members.0.js
1
species

species
1
1721

no rank
node166.members.0.js
1121353
1

species
1
571915
node167.members.0.js

species
1
575200

no rank
1224163
node169.members.0.js
1

1
161895
node170.members.0.js
species

species
1
169292

no rank
node172.members.0.js
548476
1

species
152794
3

3
node174.members.0.js
196164
no rank

1223514
1
species

1
node176.members.0.js
1223515
no rank

2
92706
species

no rank
2
node178.members.0.js
1232385

1652495
node179.members.0.js
6
species

species
5575
1718
node180.members.0.js
5996

3
node181.members.0.js
1232383
no rank

4
node182.members.0.js
1232384
no rank

22
41
196627
node183.members.0.js
no rank

no rank
1204414
node184.members.0.js
19

1079988
node185.members.0.js
66
no rank

no rank
18
1232381
node186.members.0.js

node187.members.0.js
340322
289
no rank

161879
1
species

1
node189.members.0.js
645127
no rank

species
4
1408191

4
931089
node191.members.0.js
no rank

species
2
node192.members.0.js
1050174

species
node193.members.0.js
1705
2

order
85010
1

1
2070
family

genus
65496
1

1
340345
node197.members.0.js
species

4
85011
order

2062
4
family

node200.members.0.js
1883
4
2
genus

146923
node201.members.0.js
1
species

species
1914
1

subspecies
node203.members.0.js
58340
1

1
84995
class

order
84996
1

1
84997
family

genus
42255
1

species
1
49319

1
266117
node209.members.0.js
no rank

1798711
2
no rank

1117
2
phylum

order
1
2
1161
node212.members.0.js

family
1
1162

1
56106
genus

species
142864
1

1
56107
node216.members.0.js
no rank

200918
1
phylum

class
188708
1

order
2419
1

family
188709
1

genus
2335
node221.members.0.js
1

1783257
1
no rank

1
203682
phylum

1
203683
class

1
112
order

family
1
126

genus
1
1676125

1
node228.members.0.js
1331910
species

no rank
5
1783270

no rank
68336
5

phylum
5
976

class
1
200643

1
171549
order

family
1
815

genus
1
816

species
821
1

node237.members.0.js
435590
1
no rank

class
2
768503

2
768507
order

1124781
1
no rank

1433993
1
genus

1
node242.members.0.js
2321403
species

family
1853232
1

89966
1
genus

species
1
node245.members.0.js
1484116

class
2
117743

200644
2
order

2
49546
family

genus
143222
1

143223
node250.members.0.js
1
species

genus
1
178469

species
1
node252.members.0.js
616991

1224
node253.members.0.js
76476
48
phylum

68525
7
subphylum

29547
1
class

order
213849
1

family
1
72294

genus
1
194

species
1
197

subspecies
1
32022

683082
node261.members.0.js
1
no rank

28221
6
class

order
2
29

suborder
80811
2

1
39
family

genus
40
1

species
41
1

no rank
1
378806
node268.members.0.js

family
1524215
1

1
161492
node270.members.0.js
genus

order
213462
2

family
2
213465

29526
2
genus

species
119484
2

335543
node275.members.0.js
2
no rank

69541
1
order

213422
1
family

genus
28231
1

node279.members.0.js
443143
1
species

1
213115
order

family
1
194924

genus
872
1

184917
1
species

1
node284.members.0.js
573370
no rank

class
28211
12

766
1
order

942
1
family

1
943
genus

species group
1
106178

1
35795
species

1
node291.members.0.js
1423892
no rank

order
356
7

1
118882
family

genus
1
234
node294.members.0.js

82115
2
family

no rank
2
227290

genus
1
357

species
1842536
node298.members.0.js
1

genus
1
1525371

species
node300.members.0.js
1825976
1

family
69277
1

genus
1
28100

species
1
1867719
node303.members.0.js

41294
3
family

374
2
genus

node306.members.0.js
1839752
1
species

44255
1
species

no rank
1
1245469
node308.members.0.js

genus
1
1073

1
1076
species

1
node311.members.0.js
316058
no rank

204441
1
order

433
1
family

1602345
1
genus

species
1
node315.members.0.js
1510841

204455
2
order

family
1
2
31989
node317.members.0.js

1
188905
genus

1
node319.members.0.js
290400
species

204457
1
order

family
1
41297

1
13687
genus

160791
1
species

no rank
1283312
node324.members.0.js
1

class
16
node325.members.0.js
1236
52842

order
1
135623

family
641
1

662
1
genus

species group
717610
1

species
680
node330.members.0.js
1

118969
2
order

family
444
2

2
445
genus

species
446
node334.members.0.js
1

node335.members.0.js
452
1
species

order
1
135613

family
72276
1

85108
1
genus

species
1052
node339.members.0.js
1

135622
2
order

family
1
267891

1
58050
genus

69539
node343.members.0.js
1
species

family
1
267888

1
node345.members.0.js
53246
genus

2
135619
order

family
1
28256
node347.members.0.js

family
135620
1

1
187492
genus

187493
1
species

1298593
node351.members.0.js
1
no rank

order
135624
1

family
84642
1

642
1
genus

species
1
654

1
998088
node356.members.0.js
no rank

1
135618
order

1
403
family

genus
1
413

species
1
414

1
243233
node361.members.0.js
no rank

node362.members.0.js
91347
44633
67
order

family
9482
1903411
node363.members.0.js
2

genus
node364.members.0.js
613
9476
5

species
615
node365.members.0.js
2

species
2
82996
node366.members.0.js
1

no rank
1
1006598
node367.members.0.js

node368.members.0.js
47917
9467
species

1
node369.members.0.js
629
4
genus

species group
2
1649845

species
1
node371.members.0.js
632
2

no rank
1345702
node372.members.0.js
1

species
1
node373.members.0.js
28152

23660
1903410
node374.members.0.js
14
family

genus
node375.members.0.js
204037
23642
919

node376.members.0.js
1224145
1
species

2037915
node377.members.0.js
2
species

species
204042
node378.members.0.js
22
12

node379.members.0.js
590409
4
no rank

node380.members.0.js
1224147
1
no rank

node381.members.0.js
1223567
4
no rank

1
1223573
node382.members.0.js
no rank

node383.members.0.js
1778540
2
species

18
node384.members.0.js
556
6
species

no rank
node385.members.0.js
1224148
1

no rank
4
1223571
node386.members.0.js

no rank
7
1223569
node387.members.0.js

species
22368
node388.members.0.js
1089444
22578

129
node389.members.0.js
1225786
no rank

no rank
15
node390.members.0.js
1224152

66
node391.members.0.js
1224151
no rank

species
3
69223

1224150
node393.members.0.js
3
no rank

species
node394.members.0.js
204039
23
21

1225780
node395.members.0.js
1
no rank

1
1223570
node396.members.0.js
no rank

node397.members.0.js
568766
14
species

60
node398.members.0.js
204038
28
species

no rank
node399.members.0.js
198628
5

subspecies
204040
16

no rank
node401.members.0.js
1223574
16

no rank
1224149
node402.members.0.js
11

1
71655
genus

species
node404.members.0.js
1109412
1

3
122277
node405.members.0.js
1
genus

1
554
species

1
555
subspecies

561230
node408.members.0.js
1
no rank

1
29471
node409.members.0.js
species

family
1903409
5

3
53335
node411.members.0.js
1
genus

species
1235990
node412.members.0.js
1

species
node413.members.0.js
1891675
1

genus
551
2

species
node415.members.0.js
215689
1

1
node416.members.0.js
1922217
species

543
node417.members.0.js
11412
48
family

158483
1
genus

species
158822
node419.members.0.js
1

4
191675
no rank

2
84563
no rank

genus
1
1906657

species
node423.members.0.js
1778262
1

1
146507
no rank

species
node425.members.0.js
134287
1

no rank
2
36866

node427.members.0.js
891974
1
species

1
2282309
node428.members.0.js
species

139
8716
547
node429.members.0.js
genus

2
node430.members.0.js
1692238
species

species
1868135
node431.members.0.js
1

2879
node432.members.0.js
354276
8573
species group

species
27
1812935
node433.members.0.js

42
168
node434.members.0.js
61645
species

no rank
node435.members.0.js
640513
106

no rank
20
1421338
node436.members.0.js

species
4
2077136
node437.members.0.js

1070
node438.members.0.js
550
914
species

no rank
7
1045856
node439.members.0.js

subspecies
36
336306

no rank
node441.members.0.js
716541
16

20
1211025
node442.members.0.js
no rank

node443.members.0.js
1333850
1
no rank

node444.members.0.js
1354030
9
no rank

103
69219
subspecies

1104326
node446.members.0.js
103
no rank

species
2027919
node447.members.0.js
388

11
1915310
node448.members.0.js
species

294
158836
node449.members.0.js
3854
species

subspecies
73
1812934
node450.members.0.js

subspecies
117
1296536
node451.members.0.js

subspecies
2888
299766
node452.members.0.js

301102
node453.members.0.js
6
subspecies

subspecies
301105
node454.members.0.js
476

species
node455.members.0.js
69218
135

species
13
2077137
node456.members.0.js

node457.members.0.js
299767
15
species

species
9
208224
node458.members.0.js

species
node459.members.0.js
881260
1

160674
2
genus

species
1
54291
node461.members.0.js

1
node462.members.0.js
575
species

1
1330545
genus

node464.members.0.js
1907578
1
species

genus
node465.members.0.js
83654
2

genus
590
11

5
11
node467.members.0.js
28901
species

subspecies
6
59201

no rank
2
611

no rank
2
1160717
node470.members.0.js

no rank
node471.members.0.js
600
1

no rank
2
149539
node472.members.0.js

node473.members.0.js
58712
1
no rank

2580
node474.members.0.js
413496
51
genus

species
1
1163710

no rank
1073999
node476.members.0.js
1

28141
node477.members.0.js
2519
2241
species

no rank
251
node478.members.0.js
1138308

no rank
18
956149
node479.members.0.js

no rank
9
290339
node480.members.0.js

413501
2
species

no rank
2
1159613
node482.members.0.js

species
3
413497

413498
3
subspecies

no rank
node485.members.0.js
1159554
3

413502
4
species

4
node487.members.0.js
693216
no rank

1
544
node488.members.0.js
3
genus

node489.members.0.js
1920110
1
species

species
67825
1

no rank
node491.members.0.js
637910
1

genus
929812
3

node493.members.0.js
929813
3
species

genus
1
570
node494.members.0.js
14

species
7
10
node495.members.0.js
573

2
72407
node496.members.0.js
subspecies

no rank
1049565
node497.members.0.js
1

1
1905288
node498.members.0.js
species

node499.members.0.js
244366
2
species

genus
561
25

17
24
node501.members.0.js
562
species

no rank
node502.members.0.js
1050617
2

no rank
930406
node503.members.0.js
2

no rank
1
866768
node504.members.0.js

83333
1
no rank

1
511145
no rank

node507.members.0.js
879462
1
no rank

83334
1
no rank

no rank
1
node509.members.0.js
1328859

species
208962
node510.members.0.js
1

genus
1
1330547

1
1158459
species

no rank
1
1235834
node513.members.0.js

1
1330546
node514.members.0.js
genus

family
1903414
2

genus
626
2

species
node517.members.0.js
40576
1

1
40577
species

no rank
1354304
node519.members.0.js
1

1
1903416
family

1
82984
genus

1
node522.members.0.js
82985
species

4
1903412
family

genus
4
635

species
67780
node525.members.0.js
4

order
26
72274

3
468
family

genus
469
2

species
node529.members.0.js
108981
1

species
2004647
node530.members.0.js
1

54393
1
no rank

species
1
node532.members.0.js
2283318

135621
23
family

22
node534.members.0.js
286
1
genus

17
136841
species group

1232139
2
species subgroup

1
1149133
node537.members.0.js
species

node538.members.0.js
330
1
species

1
300
node539.members.0.js
species

species
14
node540.members.0.js
287

136843
1
species group

294
1
species

no rank
1
746360
node543.members.0.js

species group
136842
2

species
587753
node545.members.0.js
2
1

86192
node546.members.0.js
1
subspecies

species
1
node547.members.0.js
237610

subfamily
1
351

352
1
genus

species
1
353

no rank
node551.members.0.js
1328314
1

1934945
1
order

family
1934946
1

genus
1
1934947

1810504
node555.members.0.js
1
species

8155
135614
order

family
8
node557.members.0.js
32033
8154

genus
41
node558.members.0.js
338
8145

1
442694
node559.members.0.js
species

species
3
5
node560.members.0.js
56460

2
node561.members.0.js
925775
no rank

584
339
node562.members.0.js
8094
species

no rank
node563.members.0.js
359385
26
15

11
node564.members.0.js
990315
no rank

92826
node565.members.0.js
17
no rank

6351
node566.members.0.js
340
7467
no rank

no rank
34
node567.members.0.js
1358017

no rank
43
314565
node568.members.0.js

node569.members.0.js
1358019
19
no rank

no rank
3
node570.members.0.js
1358009

3
1357999
node571.members.0.js
no rank

no rank
node572.members.0.js
1358015
53

190485
node573.members.0.js
960
no rank

1
node574.members.0.js
1281282
no rank

2
node575.members.0.js
48664
species

species
56448
1

1
node577.members.0.js
195709
no rank

species
347
1

1
64187
node579.members.0.js
no rank

2370
1
genus

node581.members.0.js
1444770
1
species

1775411
1
family

75309
1
genus

1
666685
node584.members.0.js
species

135625
1
order

family
1
712

1
2094023
genus

1
738
node588.members.0.js
species

6
28216
node589.members.0.js
23567
class

11
node590.members.0.js
80840
12355
order

family
20
12329
506
node591.members.0.js

genus
12300
222
node592.members.0.js
115

species
1
2282475
node593.members.0.js

32002
node594.members.0.js
2
species

species
2
node595.members.0.js
217204

node596.members.0.js
217203
2
species

12177
85698
node597.members.0.js
10274
species

461
node598.members.0.js
562971
no rank

node599.members.0.js
762376
448
no rank

no rank
1167634
node600.members.0.js
994

node601.members.0.js
1881016
1
species

genus
517
node602.members.0.js
9
3

species
1697043
node603.members.0.js
1

species
1
1331258
node604.members.0.js

1
1746199
node605.members.0.js
species

species
1
node606.members.0.js
1416803

species
94624
node607.members.0.js
1

node608.members.0.js
519
1
species

4
75682
family

genus
2
149698

species
47229
node611.members.0.js
1

species
1
node612.members.0.js
1678028

genus
2
963

1
92645
node614.members.0.js
species

80842
1
species

no rank
1
1078773
node616.members.0.js

80864
5
family

genus
12916
4

1
232721
node619.members.0.js
species

species
1
80867

subspecies
80870
node621.members.0.js
1

species
2
358220
node622.members.0.js

node623.members.0.js
80865
1
genus

family
119060
5

genus
1
106589

node626.members.0.js
164546
1
species

3
node627.members.0.js
32008
2
genus

1
87882
species group

95486
1
species

no rank
406425
node630.members.0.js
1

genus
1822464
1

1
252970
species

no rank
1
1229205
node633.members.0.js

no rank
1
119065

224471
1
no rank

genus
32012
1

species
1
node637.members.0.js
1050370

order
3
206389

family
75787
1

1
551759
genus

species
1
551760

no rank
1
76114
node642.members.0.js

family
2008794
2

2
33057
genus

species
1
96773
node645.members.0.js

1
node646.members.0.js
2005884
species

2
11203
node647.members.0.js
206351
order

5
1499392
node648.members.0.js
11201
family

1
568394
genus

1
node650.members.0.js
748280
species

57479
1
genus

species
node652.members.0.js
57480
1

genus
187
1

1
node654.members.0.js
1938604
species

no rank
11193
90153

77
535
node656.members.0.js
11193
genus

species
node657.members.0.js
1778675
2

species
1108595
node658.members.0.js
3

species
727
11110
node659.members.0.js
536

no rank
243365
node660.members.0.js
10383

species
1
node661.members.0.js
2202141

superkingdom
10239
1

35237
1
no rank

1
549779
node664.members.0.js
family
