# Supplementary material for: Benchmarking the MinION: Evaluating long reads for microbial profiling
Source: Sci Rep. 2020 Mar 20;10:5125. doi: 10.1038/s41598-020-61989-x (PMC7083898; doi:10.1038/s41598-020-61989-x)
Supplement: Supplementary file 2 — Supplementary information2. [file 41598_2020_61989_MOESM2_ESM.zip › sample_barcode_4/centrifuge.html]

Javascript must be enabled to view this page.

members
magnitude
magnitudeUnassigned
count
unassigned
taxon
rank

BC4\_k1\_centrifuge\_results

3
node0.members.0.js
148393

2157
5
superkingdom

phylum
4
28890

2290931
no rank
3

183963
3
class

1644055
order
2

family
1
1644056

2251
genus
1

species
1
node8.members.0.js
2246

no rank
1
2116545

species
756883
1
node10.members.0.js

order
1
2235

family
1
2236

1
genus
1980514

species
1873524
1
node14.members.0.js

2283794
no rank
1

183925
class
1

2158
1
order

2159
1
family

1
genus
2172

species
1
node20.members.0.js
294671

no rank
1
1783275

28889
phylum
1

183924
1
class

114380
order
1

2272
family
1

1
genus
2273

species
1
node27.members.0.js
94694

node28.members.0.js
806

superkingdom
13
147579
node29.members.0.js
2

1783272
no rank
46072

1239
2
33233
node31.members.0.js
phylum

class
1
1737404

1737405
order
1

1570339
family
1

165779
1
node35.members.0.js
genus

526524
1
class

526525
order
1

128827
family
1

1729679
1
genus

1702221
1
node40.members.0.js
species

class
8
186801

68295
2
order

family
1
543372

252965
genus
1

252966
1
node45.members.0.js
species

186814
1
family

129957
genus
1

species
129958
node48.members.0.js
1

53433
order
1

972
1
family

genus
1
32636

1
node52.members.0.js
31909
species

186802
5
order

family
1
543349

1
genus
2733

2734
1
node56.members.0.js
species

541000
1
family

genus
1
946234

node59.members.0.js
1
292800
species

31979
1
family

1981033
1
genus

species
2086584
1
node62.members.0.js

2
family
186807

genus
1562
1
node64.members.0.js

genus
1
471826

species
471827
1
node66.members.0.js

91061
2
node67.members.0.js
33221
class

186826
order
7

2
family
81852

1
genus
1350

1352
node71.members.0.js
1
species

genus
1
33969

species
33970
1
node73.members.0.js

1300
family
2

2
genus
1301

671232
1
species group

1328
1
node77.members.0.js
species

species
node78.members.0.js
1
1825069

2
family
33958

2
genus
1578

1074467
node81.members.0.js
1
species

1
node82.members.0.js
1584
species

81850
1
family

genus
1
1243

979982
node85.members.0.js
1
species

1385
33212
node86.members.0.js
4
order

6894
family
90964

1279
16
node88.members.0.js
6894
genus

species
70255
2
node89.members.0.js

species
node90.members.0.js
2
1715860

29382
3
node91.members.0.js
species

1282
node92.members.0.js
4
species

2
node93.members.0.js
1286
species

species
node94.members.0.js
6854
29385

species
node95.members.0.js
7
1280

species
node96.members.0.js
1
1288

species
node97.members.0.js
3
246432

family
3
186818

1372
genus
3

node100.members.0.js
3
2058136
species

539002
no rank
1

1
no rank
539742

33986
genus
1

species
1224749
node104.members.0.js
1

186822
13504
family

genus
44249
13503
node106.members.0.js
15

species
189426
node107.members.0.js
13481

2044880
1
species group

1
node109.members.0.js
483937
species

1464
1
node110.members.0.js
species

species
414771
1
node111.members.0.js

node112.members.0.js
3
1536775
species

1536770
1
node113.members.0.js
species

1
no rank
85151

55079
1
genus

species
node116.members.0.js
1
1500254

2
family
186820

2
genus
1637

species
1
node119.members.0.js
1639

1640
1
node120.members.0.js
species

12804
family
186817

genus
12803
node122.members.0.js
5954
1386

species group
14
86661

1
node124.members.0.js
1428
species

species
13
node125.members.0.js
1396

412384
1
node126.members.0.js
species

1
node127.members.0.js
1409
species

node128.members.0.js
1
1837130
species

node129.members.0.js
8
1664069
species

species
1856406
70
node130.members.0.js

653685
6684
species group

species
node132.members.0.js
170
1648923

node133.members.0.js
1
1423
species

1402
6503
node134.members.0.js
species

node135.members.0.js
8
119858
species

1938374
species subgroup
2

492670
2
node137.members.0.js
species

species
79885
node138.members.0.js
1

species
node139.members.0.js
2
1398

67
node140.members.0.js
2026248
species

genus
129337
node141.members.0.js
1

no rank
2
1798711

2
phylum
1117

order
2
1890424

1213
1
family

1218
1
genus

1501269
node147.members.0.js
1
species

1890426
1
family

167375
genus
1

species
1851505
1
node150.members.0.js

phylum
12834
201174

12834
class
1760

order
3940
85006

1
family
85016

genus
1
1707

1711
node156.members.0.js
1
species

85023
2
family

33882
genus
1

species
node159.members.0.js
1
36805

1759331
genus
1

1619308
1
node161.members.0.js
species

3937
family
1268

genus
1
32207

node164.members.0.js
1
43675
species

1269
genus
3936

species
node166.members.0.js
3936
1270

order
10
85011

2062
10
family

genus
9
node169.members.0.js
1
1883

1841249
node170.members.0.js
1
species

species
2094021
1
node171.members.0.js

species
68192
1
node172.members.0.js

species
47763
node173.members.0.js
1

species
node174.members.0.js
1
1912

1
node175.members.0.js
164348
species

1915
node176.members.0.js
1
species

1852274
species group
1

species
1888
node178.members.0.js
1

2063
genus
1

2018025
node180.members.0.js
1
species

4
order
85009

1
family
85015

1
genus
2044

species
2045
1
node184.members.0.js

31957
3
family

genus
1
29404

species
29405
node187.members.0.js
1

genus
2
1912216

species
2
node189.members.0.js
1747

order
2
85008

28056
family
2

1
genus
84593

species
node193.members.0.js
1
1003110

genus
1
673534

2108470
1
node195.members.0.js
species

order
1
2037

2049
1
family

1
genus
1654

1852377
1
node199.members.0.js
species

85012
1
order

2012
1
family

2019
1
genus

species
2020
1
node203.members.0.js

85010
6
order

family
node205.members.0.js
6
1
2070

43356
genus
1

43357
node207.members.0.js
1
species

genus
1
1813

1814
1
node209.members.0.js
species

1
genus
2029

species
node211.members.0.js
1
860235

genus
1
65496

1
node213.members.0.js
1612552
species

142577
genus
1

species
530584
1
node215.members.0.js

85007
order
8870

1653
8860
family

1716
8860
node218.members.0.js
222
genus

1718
node219.members.0.js
8584
species

108486
node220.members.0.js
1
species

species
191610
1
node221.members.0.js

1705
1
node222.members.0.js
species

1
node223.members.0.js
152794
species

92706
46
node224.members.0.js
species

species
1404244
1
node225.members.0.js

1721
1
node226.members.0.js
species

species
node227.members.0.js
1
1652495

species
349751
1
node228.members.0.js

85025
family
2

genus
1827
1
node230.members.0.js

1817
genus
1

species
37329
1
node232.members.0.js

8
family
1762

3
genus
1763

120793
1
species group

1764
1
node236.members.0.js
species

1
node237.members.0.js
1682113
species

species
2051552
1
node238.members.0.js

1866885
3
genus

node240.members.0.js
1
1810
species

134601
1
node241.members.0.js
species

species
node242.members.0.js
1
110539

genus
2
670516

36809
1
node244.members.0.js
species

node245.members.0.js
1
1578165
species

200795
phylum
1

1
class
475962

1
order
475963

1
family
475964

1
genus
233191

species
133453
1
node251.members.0.js

2
phylum
1297

2
class
188787

1
order
68933

family
1
188786

186191
1
genus

node257.members.0.js
1
186192
species

order
1
118964

family
1
183710

1298
genus
1

1
node261.members.0.js
432329
species

phylum
101476
node262.members.0.js
49
1224

28211
1
30
node263.members.0.js
class

5
order
204457

family
4
41297

genus
1
165695

13690
node267.members.0.js
1
species

165697
3
genus

2
node269.members.0.js
33050
species

33052
1
node270.members.0.js
species

1
family
335929

1111
1
node272.members.0.js
genus

order
6
204455

6
family
31989

1759396
genus
1

species
1920883
node276.members.0.js
1

265
1
genus

1077935
node278.members.0.js
1
species

1060
1
genus

species
1
node280.members.0.js
2033869

60136
genus
1

species
1
node282.members.0.js
1402135

1
genus
367771

species
42444
node284.members.0.js
1

302485
genus
1

species
1
node286.members.0.js
1580596

3
order
204458

76892
family
3

41275
1
genus

species
1938605
node290.members.0.js
1

75
genus
2

1
node292.members.0.js
366602
species

1
node293.members.0.js
155892
species

5
order
204441

433
1
family

1
genus
125216

species
node297.members.0.js
1
2018065

4
family
41295

1182780
genus
1

species
node300.members.0.js
1
1288970

171436
2
genus

species
2
node302.members.0.js
171437

genus
1
1081

34018
1
node304.members.0.js
species

order
10
356

41294
family
1

1
genus
374

species
288000
node308.members.0.js
1

family
4
82115

227292
2
no rank

28105
1
genus

no rank
1
663276

species
380
1
node313.members.0.js

106591
genus
1

node315.members.0.js
1
106592
species

1
genus
34019

34020
node317.members.0.js
1
species

no rank
1
227290

379
1
genus

384
1
node320.members.0.js
species

1
family
31993

genus
1
133

species
655015
1
node323.members.0.js

119045
family
1

407
genus
1

270351
1
node326.members.0.js
species

45401
family
2

1068
genus
1

1
node329.members.0.js
1069
species

1
genus
29407

674703
node331.members.0.js
1
species

69277
family
1

68287
1
genus

species
39645
node334.members.0.js
1

11
69811
node335.members.0.js
1236
class

order
91347
36
58019
node336.members.0.js

543
64
21334
node337.members.0.js
family

579
genus
1

species
61648
node339.members.0.js
1

587
node340.members.0.js
11685
547
genus

species
1914861
node341.members.0.js
1

11008
species group
354276

species
2077137
1
node343.members.0.js

species
299767
2
node344.members.0.js

2
node345.members.0.js
61645
species

species
node346.members.0.js
1
1812935

species
node347.members.0.js
3361
550

species
7641
node348.members.0.js
7627
158836

subspecies
1812934
5
node349.members.0.js

9
node350.members.0.js
1296536
subspecies

species
node351.members.0.js
89
2051905

544
2
node352.members.0.js
12
genus

species
35703
1
node353.members.0.js

1344959
9
species group

67827
node355.members.0.js
1
species

546
node356.members.0.js
7
species

species
1
node357.members.0.js
2066049

2
genus
1048757

node359.members.0.js
2
1048758
species

3
genus
158483

158822
node361.members.0.js
3
species

1330545
genus
1

61646
1
node363.members.0.js
species

413496
33
9504
node364.members.0.js
genus

species
413497
node365.members.0.js
2

93
node366.members.0.js
413503
species

node367.members.0.js
6
413501
species

species
535744
node368.members.0.js
11

28141
9352
node369.members.0.js
species

7
node370.members.0.js
413502
species

561
genus
27

species
1499973
1
node372.members.0.js

562
node373.members.0.js
26
species

1330547
3
genus

species
node375.members.0.js
1
283686

node376.members.0.js
1
497725
species

node377.members.0.js
1
208223
species

1
7
node378.members.0.js
590
genus

species
28901
node379.members.0.js
6

1330546
genus
2

1334193
1
node381.members.0.js
species

species
node382.members.0.js
1
61647

1
genus
409304

species
node384.members.0.js
1
168169

8
no rank
191675

36866
no rank
4

node387.members.0.js
2
693444
species

1920109
node388.members.0.js
1
species

species
1
node389.members.0.js
891974

84563
no rank
4

1682492
1
genus

node392.members.0.js
1
1410383
species

1
genus
1906661

1070130
1
node394.members.0.js
species

no rank
2
84564

2
genus
203804

no rank
2
711328

species
node398.members.0.js
2
1505596

1
genus
1335483

node400.members.0.js
1
563
species

genus
2
node401.members.0.js
13
570

6
node402.members.0.js
573
species

species
1463165
1
node403.members.0.js

species
571
node404.members.0.js
3

species
node405.members.0.js
1
1134687

family
1903410
2
node406.members.0.js
20757

genus
204037
20750
node407.members.0.js
97

species
node408.members.0.js
57
204038

10
node409.members.0.js
204042
species

node410.members.0.js
20537
1089444
species

node411.members.0.js
20
1778540
species

204039
node412.members.0.js
29
species

genus
1
84565

1239307
1
node414.members.0.js
species

122277
2
4
node415.members.0.js
genus

species
node416.members.0.js
1
55208

species
554
1
node417.members.0.js

family
7
1903414

586
genus
2

588
1
node420.members.0.js
species

species
126385
node421.members.0.js
1

1
genus
581

582
node423.members.0.js
1
species

1
genus
583

species
584
1
node425.members.0.js

29487
genus
2

574560
node427.members.0.js
2
species

genus
1
626

node429.members.0.js
1
351671
species

15867
family
1903411

34037
1
node431.members.0.js
2
genus

node432.members.0.js
1
1805933
species

613
node433.members.0.js
15861
4
genus

species
node434.members.0.js
24
615

species
104623
2
node435.members.0.js

species
15827
node436.members.0.js
47917

species
61651
1
node437.members.0.js

species
node438.members.0.js
1
488142

82996
node439.members.0.js
2
species

1
node440.members.0.js
4
629
genus

species group
1
1649845

species
node442.members.0.js
1
632

630
2
node443.members.0.js
species

1903412
11
family

genus
11
635

67780
9
node446.members.0.js
species

species
636
node447.members.0.js
2

1903409
7
family

genus
53335
node449.members.0.js
2
1

1
node450.members.0.js
592316
species

4
genus
551

species
node452.members.0.js
2
215689

species
1619313
1
node453.members.0.js

species
182337
1
node454.members.0.js

2100764
1
genus

node456.members.0.js
1
665913
species

118884
no rank
1

198346
no rank
1

species
1
node459.members.0.js
186490

135614
order
11737

family
1
11736
node461.members.0.js
32033

338
26
node462.members.0.js
11730
genus

species
node463.members.0.js
1
56454

643453
species group
6

species
6
node465.members.0.js
346

species
29447
node466.members.0.js
1

node467.members.0.js
1
48664
species

species
56460
3
node468.members.0.js

2
node469.members.0.js
90270
species

339
node470.members.0.js
11689
species

species
442694
node471.members.0.js
1

genus
1
68

69
1
node473.members.0.js
species

83618
1
genus

node475.members.0.js
1
415229
species

40323
3
node476.members.0.js
1
genus

1904944
1
node477.members.0.js
species

995085
1
species group

species
1
node479.members.0.js
40324

1
family
1775411

2233801
genus
1

2021234
node482.members.0.js
1
species

2
order
135623

2
family
641

2
genus
662

62153
node486.members.0.js
1
species

1
species group
717610

670
1
node488.members.0.js
species

135613
2
order

1
family
72276

133193
genus
1

species
351052
node492.members.0.js
1

1046
1
family

85072
1
genus

species
1
node495.members.0.js
1049

118969
order
1

444
1
family

genus
1
445

1
node499.members.0.js
28087
species

2
order
1706369

1
family
1706373

48073
genus
1

node503.members.0.js
1
252514
species

family
1
1706371

1
genus
10

155077
1
node506.members.0.js
species

135624
2
order

84642
family
2

642
2
genus

species
651
node510.members.0.js
1

node511.members.0.js
1
644
species

135622
5
order

1
family
267888

53246
genus
1

1
node515.members.0.js
161398
species

2
family
72275

genus
1
2742

1
node518.members.0.js
330734
species

226
genus
1

233316
node520.members.0.js
1
species

267893
family
1

135575
1
genus

2100422
node523.members.0.js
1
species

267890
family
1

22
1
genus

species
node526.members.0.js
1
93973

72274
29
order

family
28
135621

28
node529.members.0.js
2
286
genus

species group
1
136849

species
36746
node531.members.0.js
1

1
species group
136843

species
294
1
node533.members.0.js

136842
species group
2

587753
2
node535.members.0.js
species

species
1283291
1
node536.members.0.js

136845
1
species group

species
node538.members.0.js
1
303

species group
1
136846

species subgroup
1
578833

316
1
node541.members.0.js
species

species
1
node542.members.0.js
1853130

species group
18
136841

species
18
node544.members.0.js
287

468
1
family

1
genus
469

909768
1
species group

species
node548.members.0.js
1
471

class
node549.members.0.js
31581
5
28216

17
17400
node550.members.0.js
80840
order

7
node551.members.0.js
3
80864
family

12916
2
genus

species
80867
node553.members.0.js
1

species
232721
1
node554.members.0.js

1
genus
238749

1546149
1
node556.members.0.js
species

genus
1
219181

species
1
node558.members.0.js
1658672

119060
family
19

1
genus
1822464

species
node561.members.0.js
1
261302

106589
genus
3

species
876364
1
node563.members.0.js

species
node564.members.0.js
1
106590

119219
node565.members.0.js
1
species

4
node566.members.0.js
11
32008
genus

111527
species group
1

species
28450
node568.members.0.js
1

1
node569.members.0.js
28095
species

species
node570.members.0.js
3
640510

species group
2
87882

node572.members.0.js
1
101571
species

node573.members.0.js
1
488446
species

48736
genus
3

species
305
3
node575.members.0.js

93217
1
node576.members.0.js
genus

no rank
4
119065

224471
3
no rank

93681
1
genus

76731
node580.members.0.js
1
species

32012
genus
2

species
926
node582.members.0.js
2

species
1
node583.members.0.js
413882

506
node584.members.0.js
17351
8
family

genus
1
1921582

species
1851544
1
node586.members.0.js

359336
1
genus

75697
1
node588.members.0.js
species

8
node589.members.0.js
1
517
genus

1
node590.members.0.js
463040
species

species
node591.members.0.js
2
94624

node592.members.0.js
1
1331258
species

35814
1
node593.members.0.js
species

520
node594.members.0.js
1
species

species
103855
node595.members.0.js
1

222
37
node596.members.0.js
17333
genus

species
node597.members.0.js
38
32002

1758194
node598.members.0.js
5
species

species
85698
17247
node599.members.0.js

species
node600.members.0.js
3
217204

species
node601.members.0.js
3
217203

2
family
75682

1
node603.members.0.js
29580
genus

149698
1
genus

species
1
node605.members.0.js
2045208

206351
order
14172

family
1
481

482
genus
1

1
node609.members.0.js
487
species

1
14171
node610.members.0.js
1499392
family

90153
14169
no rank

535
28
14169
node612.members.0.js
genus

5
node613.members.0.js
2059672
species

species
node614.members.0.js
14117
536

species
19
node615.members.0.js
1108595

168470
1
genus

species
1
node617.members.0.js
168471

206389
1
order

75787
family
1

146937
genus
1

1
node621.members.0.js
146939
species

order
3
32003

32011
family
1

1679002
genus
1

1581557
1
node625.members.0.js
species

206379
family
1

914
genus
1

node628.members.0.js
1
44574
species

2008793
1
family

378210
genus
1

species
1
node631.members.0.js
1842540

68525
5
subphylum

4
class
28221

1
order
69541

family
1
213422

28231
1
genus

225194
1
node637.members.0.js
species

1
order
29

1
suborder
80811

family
39
node640.members.0.js
1

1
order
213115

194924
1
family

872
genus
1

881
node644.members.0.js
1
species

213462
1
order

213468
1
family

2357
genus
1

1
node648.members.0.js
2358
species

29547
class
1

1
order
213849

family
1
72294

57665
1
genus

node653.members.0.js
1
66821
species

203691
phylum
2

2
class
203692

2
no rank
1643688

170
family
2

genus
171
2
node658.members.0.js
1

node659.members.0.js
1
172
species

1783257
3
no rank

3
phylum
203682

203683
3
class

3
order
112

2
family
1763524

2
genus
127

species
node666.members.0.js
2
128

126
family
1

genus
1
1649480

species
node669.members.0.js
1
120

no rank
12
1783270

68336
no rank
12

1090
1
phylum

1
class
191410

191411
1
order

1
family
191412

1
no rank
274493

genus
1
1099

1
node678.members.0.js
1100
species

phylum
11
976

class
1
1853228

order
1
1853229

563835
1
family

649460
1
genus

1
node684.members.0.js
477680
species

117743
3
class

200644
3
order

49546
3
family

28250
genus
1

1
node689.members.0.js
28251
species

genus
1
59732

1685010
1
node691.members.0.js
species

genus
1
237

1492737
1
node693.members.0.js
species

768503
3
class

order
3
768507

1
family
1853232

genus
1
89966

species
1484116
node698.members.0.js
1

563798
family
1

genus
1
246875

1727163
1
node701.members.0.js
species

89373
family
1

1
genus
861914

species
1834519
1
node704.members.0.js

4
class
200643

4
order
171549

1
family
2005520

294702
genus
1

1
node709.members.0.js
1642647
species

815
family
2

2
genus
816

species
1
node712.members.0.js
817

species
node713.members.0.js
1
357276

171552
family
1

genus
1
838

76123
1
node716.members.0.js
species

1
phylum
200940

class
1
67799

188710
1
order

188711
family
1

241192
1
genus

species
171695
node722.members.0.js
1
