# Supplementary material for: Benchmarking the MinION: Evaluating long reads for microbial profiling
Source: Sci Rep. 2020 Mar 20;10:5125. doi: 10.1038/s41598-020-61989-x (PMC7083898; doi:10.1038/s41598-020-61989-x)
Supplement: Supplementary file 2 — Supplementary information2. [file 41598_2020_61989_MOESM2_ESM.zip › sample_barcode_4/kraken.html]

Javascript must be enabled to view this page.

members
magnitude
magnitudeUnassigned
count
unassigned
taxon
rank

BC4\_kraken\_krona

148392
node0.members.0.js
19

2
superkingdom
26
node1.members.0.js
144480

phylum
1224
97
99273
node2.members.0.js

node3.members.0.js
68268
16
1236
class

21
72274
order

family
135621
21

21
genus
286

21
136841
species group

287
species
18
node8.members.0.js
21

node9.members.0.js
1
no rank
910265

798130
no rank
2
node10.members.0.js

116
91347
order
56693
node11.members.0.js

14
1903410
family
19982
node12.members.0.js

3
122277
genus

node14.members.0.js
3
1
species
554

555
subspecies
2

1218933
no rank
node16.members.0.js
2

node17.members.0.js
19964
204037
genus
1017

species
1224145
node18.members.0.js
2

1089444
species
18429
node19.members.0.js
18895

no rank
1225786
node20.members.0.js
348

1226344
no rank
node21.members.0.js
47

26
node22.members.0.js
no rank
1224152

1224151
no rank
node23.members.0.js
45

69223
species
1

1224150
no rank
node25.members.0.js
1

node26.members.0.js
6
556
species
2

1223569
no rank
2
node27.members.0.js

1224148
no rank
1
node28.members.0.js

1223571
no rank
node29.members.0.js
1

4
node30.members.0.js
2
204042
species

1
node31.members.0.js
590409
no rank

1
node32.members.0.js
1427366
no rank

8
node33.members.0.js
204039
species

27
node34.members.0.js
204038
species
13

6
node35.members.0.js
no rank
1224149

204040
subspecies
5

5
node37.members.0.js
no rank
1223574

1223572
no rank
3
node38.members.0.js

4
node39.members.0.js
568766
species

71655
genus
1

node41.members.0.js
1
598467
species

node42.members.0.js
15621
4
1903411
family

15614
node43.members.0.js
genus
613
9

species
61651
1
node44.members.0.js

82996
species
1

no rank
1006598
1
node46.members.0.js

node47.members.0.js
15602
47917
species

species
1759437
node48.members.0.js
1

3
node49.members.0.js
1
629
genus

node50.members.0.js
1
630
species

1
species group
1649845

1
node52.members.0.js
632
species

161
family
543
node53.members.0.js
20972

8
160674
genus

node55.members.0.js
4
species
575

54291
species
4
node56.members.0.js

561
genus
28

node58.members.0.js
28
22
species
562

585057
no rank
node59.members.0.js
1

node60.members.0.js
1
585397
no rank

910348
no rank
node61.members.0.js
1

1038927
no rank
2

no rank
1048254
node63.members.0.js
1

1133853
no rank
1
node64.members.0.js

1358422
no rank
node65.members.0.js
1

1
genus
620

622
species
1

300267
no rank
1
node68.members.0.js

1
590
genus
3
node69.members.0.js

2
28901
species

2
subspecies
59201

108619
no rank
1

no rank
877468
node73.members.0.js
1

1
340190
no rank

no rank
439843
1
node75.members.0.js

9290
node76.members.0.js
413496
genus
230

2
species
413503
node77.members.0.js
5

1159491
no rank
node78.members.0.js
3

12
413502
species

no rank
693216
12
node80.members.0.js

2
species
413501

1159613
no rank
node82.members.0.js
2

1
species
1163710

1
node84.members.0.js
1073999
no rank

species
413497
1

1
subspecies
413498

1159554
no rank
node87.members.0.js
1

9039
node88.members.0.js
species
28141
8203

403
node89.members.0.js
no rank
1138308

956149
no rank
node90.members.0.js
29

node91.members.0.js
404
no rank
290339

genus
1330546
26

species
1334193
21
node93.members.0.js
26

no rank
701347
node94.members.0.js
5

no rank
191675
1

node96.members.0.js
1
84563
no rank

11430
node97.members.0.js
genus
547
24

5944
354276
species group
11406
node98.members.0.js

species
550
671
753
node99.members.0.js

1045856
no rank
6
node100.members.0.js

subspecies
336306
13

716541
no rank
12
node102.members.0.js

no rank
1211025
node103.members.0.js
1

subspecies
69219
56

1104326
no rank
node105.members.0.js
56

no rank
1354030
7
node106.members.0.js

197
species
158836
4573
node107.members.0.js

node108.members.0.js
47
subspecies
1296536

subspecies
299766
node109.members.0.js
3706

node110.members.0.js
250
301102
subspecies

301105
subspecies
node111.members.0.js
319

subspecies
1812934
node112.members.0.js
54

1812935
species
9
node113.members.0.js

40
species
61645
108
node114.members.0.js

node115.members.0.js
52
640513
no rank

node116.members.0.js
16
no rank
1421338

node117.members.0.js
7
299767
species

node118.members.0.js
7
1915310
species

node119.members.0.js
5
species
208224

5
544
genus

1344959
species group
1
node121.members.0.js
3

node122.members.0.js
2
species
546

2
67825
species

2
node124.members.0.js
637910
no rank

node125.members.0.js
19
570
genus
3

571
species
node126.members.0.js
2

1905288
species
5
node127.members.0.js

species
573
4
5
node128.members.0.js

subspecies
72407
node129.members.0.js
1

species
548
node130.members.0.js
2

species
1134687
1

node132.members.0.js
1
1191061
no rank

species
1463165
node133.members.0.js
1

1
family
1903412

1
635
genus

node136.members.0.js
1
67780
species

1
1903414
family

genus
586
1

node139.members.0.js
1
species
588

11536
order
135614

8
family
32033
node141.members.0.js
11536

68
genus
1

1
node143.members.0.js
69
species

11527
node144.members.0.js
338
genus
103

1
343
species

487909
no rank
1
node146.members.0.js

species
339
1050
11419
node147.members.0.js

9007
340
no rank
10326
node148.members.0.js

no rank
314565
node149.members.0.js
35

node150.members.0.js
16
no rank
1281282

node151.members.0.js
3
no rank
1357999

node152.members.0.js
1
1281283
no rank

3
node153.members.0.js
no rank
1358009

node154.members.0.js
1261
190485
no rank

node155.members.0.js
24
9
359385
no rank

990315
no rank
node156.members.0.js
15

no rank
92826
node157.members.0.js
19

1
node158.members.0.js
species
456327

species group
643453
1

node160.members.0.js
1
species
346

species
48664
node161.members.0.js
2

2
135623
order

641
family
2

2
662
genus

node165.members.0.js
1
species
190893

666
species
1

1
no rank
127906

node168.members.0.js
1
no rank
1134456

17
class
28216
node169.members.0.js
30907

node170.members.0.js
17024
8
order
80840

no rank
119065
2

80841
no rank
2

2
node173.members.0.js
species
1469502

17010
node174.members.0.js
family
506
29

node175.members.0.js
16978
224
genus
222

1881016
species
1
node176.members.0.js

16749
node177.members.0.js
species
85698
14088

562971
no rank
1020
node178.members.0.js

node179.members.0.js
1429
no rank
1167634

762376
no rank
node180.members.0.js
212

node181.members.0.js
4
species
32002

node182.members.0.js
3
1
517
genus

1
node183.members.0.js
species
520

node184.members.0.js
1
94624
species

80864
family
2

1
genus
2490452

species
1458426
node187.members.0.js
1

genus
283
1

species
285
1

1
node190.members.0.js
no rank
1191062

family
119060
1
2
node191.members.0.js

106589
genus
1
node192.members.0.js

13866
node193.members.0.js
order
206351
1

13865
node194.members.0.js
family
1499392
11

90153
no rank
13852

62
genus
535
node196.members.0.js
13852

13785
536
species

no rank
243365
node198.members.0.js
13785

node199.members.0.js
5
species
1108595

57739
genus
1

node201.members.0.js
1
species
1192162

genus
187
1

1
node203.members.0.js
1938604
species

class
28211
1

order
356
1

1
family
82115

1
227292
no rank

genus
28105
1

1
node209.members.0.js
1842534
species

45181
no rank
1783272

32561
1239
phylum

1
class
526524

526525
order
1

family
128827
1

1
genus
191303

1
node216.members.0.js
1712675
species

node217.members.0.js
32560
91061
class
3

3
order
1385
node218.members.0.js
32557

12491
node219.members.0.js
186817
family
2

129337
genus
1

species group
1505648
1

species
33938
1
node222.members.0.js

12488
node223.members.0.js
genus
1386
5677

53
node224.members.0.js
species group
86661
6

species
1428
10
12
node225.members.0.js

1
no rank
29339
2
node226.members.0.js

node227.members.0.js
1
no rank
1261129

species
1396
30
34
node228.members.0.js

no rank
288681
3
node229.members.0.js

347495
no rank
1
node230.members.0.js

node231.members.0.js
1
1405
species

species
1398
node232.members.0.js
1

node233.members.0.js
1
species
1178537

1
node234.members.0.js
1547283
species

node235.members.0.js
1
86664
species

node236.members.0.js
4
756828
species

1856406
species
node237.members.0.js
12

5
node238.members.0.js
1408
species

node239.members.0.js
6699
653685
species group
563

species subgroup
1938374
1
8
node240.members.0.js

2
species
1390
node241.members.0.js
4

2
node242.members.0.js
1292358
no rank

node243.members.0.js
3
2
492670
species

1
node244.members.0.js
no rank
1458206

5542
node245.members.0.js
1402
species
3369

no rank
279010
2165
node246.members.0.js

no rank
1126218
8
node247.members.0.js

node248.members.0.js
61
species
119858

3
node249.members.0.js
1452
species
2

node250.members.0.js
1
no rank
1239783

13
1423
species
node251.members.0.js
24

node252.members.0.js
3
no rank
936156

1
subspecies
96241

no rank
655816
node254.members.0.js
1

135461
subspecies
1
6
node255.members.0.js

1
node256.members.0.js
no rank
1404258

535026
no rank
node257.members.0.js
1

node258.members.0.js
1
no rank
224308

2
node259.members.0.js
no rank
1302650

1
node260.members.0.js
86029
subspecies

476
species
1648923
node261.members.0.js
498

22
node262.members.0.js
no rank
766760

3
node263.members.0.js
561879
species

29
node264.members.0.js
species
1664069

1
species
86665

272558
no rank
node266.members.0.js
1

species
1441095
node267.members.0.js
1

90964
family
2
6767
node268.members.0.js

1279
genus
97
node269.members.0.js
6765

4
node270.members.0.js
2
28035
species

2
node271.members.0.js
no rank
1034809

node272.members.0.js
6469
29385
species
2356

147452
subspecies
4113

342451
no rank
node274.members.0.js
4113

species
308354
node275.members.0.js
1

node276.members.0.js
68
species
1280
66

subspecies
46170
2
node277.members.0.js

node278.members.0.js
8
species
214473

246432
species
node279.members.0.js
54

1
45972
species
node280.members.0.js
4

node281.members.0.js
3
no rank
1276282

70255
species
36
node282.members.0.js

node283.members.0.js
1
61015
species

1288
species
node284.members.0.js
22

1
node285.members.0.js
species
1715860

13296
node286.members.0.js
186822
family
2

13294
node287.members.0.js
genus
44249
38

1
node288.members.0.js
1619311
species

1
node289.members.0.js
1536770
species

species
189426
node290.members.0.js
13252

414771
species
1
node291.members.0.js

node292.members.0.js
1
1536775
species

12620
node293.members.0.js
phylum
201174
1

12619
node294.members.0.js
4
class
1760

85009
order
1

1
family
31957

1
genus
1912216

species
1747
node298.members.0.js
1

85006
order
3865

2
family
1268
3865
node300.members.0.js

genus
1269
3863

3507
1270
species
3863
node302.members.0.js

no rank
465515
node303.members.0.js
356

order
85007
8749

85025
family
1

1827
genus
1

1
node307.members.0.js
1805827
species

8748
family
1653

genus
1716
679
node309.members.0.js
8748

38305
species
1

1224164
no rank
1
node311.members.0.js

species
349751
1

node313.members.0.js
1
1224162
no rank

1652495
species
node314.members.0.js
3

node315.members.0.js
1
146827
species

1408191
species
1

931089
no rank
node317.members.0.js
1

8061
node318.members.0.js
7708
species
1718

no rank
340322
193
node319.members.0.js

no rank
1310161
node320.members.0.js
7

no rank
1232384
1
node321.members.0.js

34
node322.members.0.js
no rank
1079988

106
no rank
196627
118
node323.members.0.js

no rank
1204414
node324.members.0.js
12

1
node325.members.0.js
43771
species

node326.members.0.js
3893
