# Supplementary material for: Benchmarking the MinION: Evaluating long reads for microbial profiling
Source: Sci Rep. 2020 Mar 20;10:5125. doi: 10.1038/s41598-020-61989-x (PMC7083898; doi:10.1038/s41598-020-61989-x)
Supplement: Supplementary file 2 — Supplementary information2. [file 41598_2020_61989_MOESM2_ESM.zip › sample_barcode_4/kraken2.html]

Javascript must be enabled to view this page.

members
magnitude
magnitudeUnassigned
count
unassigned
taxon
rank

BC4\_kraken2\_krona

4
node0.members.0.js
148392

node1.members.0.js
1981

32
2
superkingdom
node2.members.0.js
146407

phylum
32066
1

class
203490
1

order
203491
1

203492
family
1

1
848
genus

species
849
1

469615
no rank
1
node9.members.0.js

no rank
1783270
6

6
68336
no rank

976
phylum
6

768503
class
2

order
768507
2

no rank
1124781
1

1
1433993
genus

2321403
species
node17.members.0.js
1

1853234
family
1

genus
59740
1

1085624
species
node20.members.0.js
1

3
117743
class

3
order
200644

family
49546
3

1
379070
genus

species
754429
1
node25.members.0.js

1
111500
genus

species
516051
node27.members.0.js
1

1
252356
genus

node29.members.0.js
1
species
313603

1
class
200643

order
1970189
1

1
1573805
family

1
no rank
1717716

1717717
species
1
node34.members.0.js

63
phylum
1224
100605
node35.members.0.js

1
16
node36.members.0.js
28211
class

204457
order
5

2
family
335929

1041
genus
1

node40.members.0.js
1
species
39960

genus
361177
1

692370
species
1
node42.members.0.js

41297
family
3

1
genus
165695

120107
species
node45.members.0.js
1

1
genus
13687

node47.members.0.js
1
species
160791

1
165696
genus

species
205844
1

no rank
1088721
node50.members.0.js
1

204458
order
1

1
family
76892

1
genus
75

node54.members.0.js
1
88688
species

6
order
356

family
119045
2

407
genus
2
node57.members.0.js
1

334852
species
1

1
node59.members.0.js
no rank
693986

1
82115
family

1
227292
no rank

1
106591
genus

species
716925
1

no rank
716928
1
node64.members.0.js

2
45401
family

genus
29407
1

node67.members.0.js
1
species
674703

1
81
genus

717785
species
node69.members.0.js
1

1
family
45404

1
genus
532

533
species
1

subspecies
31994
1

395963
no rank
node74.members.0.js
1

204455
order
2

family
31989
2

1
genus
92944

92945
species
node78.members.0.js
1

1
genus
60136

node80.members.0.js
1
species
1389011

order
204441
1

1
family
433

genus
89583
1

1
species
33996

272568
no rank
node85.members.0.js
1

3
subphylum
68525

1
class
28221
node87.members.0.js
2

order
29
1

80811
suborder
1

1
family
1524215

1
genus
161492

447217
species
node92.members.0.js
1

class
29547
1

order
213849
1

family
72294
1

1
57665
genus

66821
species
1

1150621
no rank
1
node98.members.0.js

class
1553900
1

1
213481
order

1
213483
family

1
genus
958

959
species
node103.members.0.js
1

9
31312
node104.members.0.js
class
28216

206389
order
1

family
2008794
1

12960
genus
1

1
node108.members.0.js
species
41977

order
80840
17265
node109.members.0.js
9

2
75682
family

149698
genus
1

1
node112.members.0.js
species
2045208

1
202907
genus

node114.members.0.js
1
species
279113

11
80864
family

1
genus
219181

1
node117.members.0.js
1658672
species

201096
genus
1

179636
species
1

596154
no rank
node120.members.0.js
1

3
node121.members.0.js
12916
genus
1

1
80867
species

1
node123.members.0.js
subspecies
80870

1
species
721785

1
node125.members.0.js
no rank
535289

genus
1649468
3

2109913
species
3
node127.members.0.js

34072
genus
1

node129.members.0.js
1
species
436515

genus
283
1

species
1082851
node131.members.0.js
1

node132.members.0.js
1
no rank
83494

node133.members.0.js
17232
506
family
24

2
node134.members.0.js
7
genus
517

1
node135.members.0.js
123899
species

node136.members.0.js
1
463025
species

node137.members.0.js
1
520
species

node138.members.0.js
1
species
94624

species
463040
node139.members.0.js
1

1921582
genus
1

1851544
species
node141.members.0.js
1

genus
90243
1

node143.members.0.js
1
species
90245

164
genus
222
17199
node144.members.0.js

species
1758194
node145.members.0.js
2

species
217203
node146.members.0.js
2

3
node147.members.0.js
species
32002

2282475
species
1
node148.members.0.js

node149.members.0.js
17025
85698
species
14278

562971
no rank
node150.members.0.js
687

638
node151.members.0.js
762376
no rank

node152.members.0.js
1422
no rank
1167634

2
node153.members.0.js
species
1881016

2
no rank
119065

2
no rank
224471

genus
88
1

1
34029
species

1
node158.members.0.js
no rank
395495

1
28067
genus

1
28068
species

983917
no rank
node161.members.0.js
1

2
node162.members.0.js
9
family
119060

93217
genus
1

1
node164.members.0.js
species
93220

genus
106589
2

species
1796606
node166.members.0.js
1

119219
species
node167.members.0.js
1

2
32008
genus
node168.members.0.js
4

1
node169.members.0.js
species group
87882

111527
species group
1
node170.members.0.js

14036
node171.members.0.js
206351
order
2

481
family
1

482
genus
1

487
species
1

node175.members.0.js
1
no rank
1095685

node176.members.0.js
14033
1499392
family
13

90153
no rank
14020

14020
node178.members.0.js
genus
535
95

species
1108595
3
node179.members.0.js

2202142
species
node180.members.0.js
1

node181.members.0.js
13914
species
536
1012

node182.members.0.js
12902
243365
no rank

6
node183.members.0.js
2059672
species

1778675
species
1
node184.members.0.js

1
order
32003

family
90627
1

genus
1778653
1

1985873
species
1
node188.members.0.js

1
class
2008785

119069
order
1

206349
family
1

genus
70774
1

species
297
1
node193.members.0.js

class
1236
69209
node194.members.0.js
18

135622
order
5

1
family
267892

1
genus
44011

1
species
44012

1
node199.members.0.js
no rank
550540

family
267888
1

53246
genus
1

species
227
1

1
node203.members.0.js
no rank
1314868

3
267890
family

genus
22
3
node205.members.0.js
1

192073
species
1

318161
no rank
1
node207.members.0.js

1
359303
species

node209.members.0.js
1
no rank
323850

1
node210.members.0.js
11644
order
135614

5
32033
family
node211.members.0.js
11643

1
genus
68
node212.members.0.js
2

species
69
node213.members.0.js
1

2
83618
genus

node215.members.0.js
2
species
314722

80
genus
338
node216.members.0.js
11634

806
species
339
node217.members.0.js
11540

92826
no rank
22
node218.members.0.js

10678
node219.members.0.js
no rank
340
8966

1358009
no rank
4
node220.members.0.js

71
node221.members.0.js
no rank
314565

1358017
no rank
node222.members.0.js
33

node223.members.0.js
28
no rank
1358019

1281282
no rank
4
node224.members.0.js

1358015
no rank
node225.members.0.js
80

190485
no rank
node226.members.0.js
1489

node227.members.0.js
3
1357999
no rank

19
359385
no rank
node228.members.0.js
34

990315
no rank
node229.members.0.js
15

species
347
node230.members.0.js
2
1

129394
no rank
1
node231.members.0.js

1
species
56448

node233.members.0.js
1
195709
no rank

2
species
56450

2
node235.members.0.js
1219375
no rank

species
56454
1

1
487904
no rank

no rank
863365
node238.members.0.js
1

species
1985254
1

1
92828
no rank

no rank
1437877
1
node241.members.0.js

3
species group
643453

346
species
node243.members.0.js
3
1

1
node244.members.0.js
76802
no rank

1
node245.members.0.js
no rank
86040

species
343
1

1
no rank
134875

node248.members.0.js
1
no rank
1261556

species
48664
node249.members.0.js
3

1
order
1934945

1
family
1934946

genus
1934947
1

species
1810504
1
node253.members.0.js

order
135623
3

family
641
3

genus
662
3

species group
717610
1

1
species
680

no rank
338187
1
node259.members.0.js

666
species
1

127906
no rank
1

1134456
no rank
1
node262.members.0.js

node263.members.0.js
1
190893
species

43
order
72274

family
468
1

1
no rank
54393

node267.members.0.js
1
2283318
species

42
family
135621

2
genus
286
node269.members.0.js
42

species group
136845
2

species
303
node271.members.0.js
1

node272.members.0.js
1
species
76759

1
136846
species group

1
species subgroup
578833

1
species
316

1196835
no rank
1
node276.members.0.js

136843
species group
2

species
294
node278.members.0.js
1

node279.members.0.js
1
species
29442

3
species group
136849

251695
species subgroup
1

1
species
317

1
node283.members.0.js
1357279
no rank

36746
species
1

1
node285.members.0.js
no rank
1441629

33069
species
node286.members.0.js
1

node287.members.0.js
1
species
2083051

136841
species group
31

287
species
node289.members.0.js
30
26

910265
no rank
node290.members.0.js
1

1427342
no rank
node291.members.0.js
2

node292.members.0.js
1
1400868
no rank

1
node293.members.0.js
53408
species

135624
order
2

2
family
84642

2
node296.members.0.js
genus
642
1

node297.members.0.js
1
558964
species

6
order
135619

1
255527
family

1
genus
1445504

species
1445505
1

no rank
1445510
node302.members.0.js
1

1920240
family
1

261963
genus
node304.members.0.js
1

1
family
2066474

1
305899
genus

1027273
species
1

570277
no rank
1
node308.members.0.js

1
family
224379

1
genus
158481

species
158327
1

349521
no rank
1
node312.members.0.js

2
family
224372

1
59753
genus
node314.members.0.js
2

1
node315.members.0.js
2014542
species

4
135625
order

4
712
family

1
genus
2094023

species
738
node319.members.0.js
1

2
genus
75984

node321.members.0.js
1
species
75985

1432056
species
node322.members.0.js
1

genus
745
1

747
species
1

node325.members.0.js
1
subspecies
44283

93
order
91347
node326.members.0.js
57481

20415
node327.members.0.js
1903410
family
13

3
genus
71655

1
node329.members.0.js
species
1109412

598467
species
node330.members.0.js
2

122277
genus
2

species
1905730
1
node332.members.0.js

species
29471
1
node333.members.0.js

20397
node334.members.0.js
204037
genus
762

16
species
204042
27
node335.members.0.js

no rank
1427366
node336.members.0.js
2

590409
no rank
node337.members.0.js
5

no rank
1224153
node338.members.0.js
1

no rank
1223567
node339.members.0.js
1

1223573
no rank
node340.members.0.js
2

556
species
node341.members.0.js
23
10

no rank
1224148
4
node342.members.0.js

node343.members.0.js
5
1223571
no rank

4
node344.members.0.js
1223569
no rank

21
22
node345.members.0.js
204039
species

node346.members.0.js
1
no rank
1223570

69223
species
4

4
node348.members.0.js
no rank
1224150

28
48
node349.members.0.js
species
204038

1224149
no rank
node350.members.0.js
7

198628
no rank
node351.members.0.js
6

1223572
no rank
node352.members.0.js
1

204040
subspecies
6

no rank
1223574
node354.members.0.js
6

1
node355.members.0.js
species
2037915

node356.members.0.js
2
species
1778540

13
node357.members.0.js
568766
species

species
1224145
node358.members.0.js
2

species
1089444
node359.members.0.js
19493
19281

no rank
1224151
67
node360.members.0.js

no rank
1225786
124
node361.members.0.js

20
node362.members.0.js
no rank
1224152

1
node363.members.0.js
no rank
1226344

3
node364.members.0.js
15744
1903411
family

genus
34037
1
node365.members.0.js

7
genus
613
15735
node366.members.0.js

82996
species
node367.members.0.js
1

47917
species
15725
node368.members.0.js

node369.members.0.js
2
species
615

629
genus
5

29486
species
1
node371.members.0.js

935293
species
node372.members.0.js
1

species
263819
node373.members.0.js
1

1
1649845
species group

species
632
1
node375.members.0.js

1
node376.members.0.js
630
species

1
10
node377.members.0.js
family
1903412

2
568
genus

2
node379.members.0.js
species
569

genus
635
7
node380.members.0.js
1

67780
species
node381.members.0.js
2

636
species
2
node382.members.0.js

1263550
species
2
node383.members.0.js

3
family
1903414

29487
genus
node385.members.0.js
1

genus
581
1

species
582
1
node387.members.0.js

1
genus
626

node389.members.0.js
1
351671
species

1903416
family
1

genus
82980
1

158841
species
1
node392.members.0.js

128
family
543
node393.members.0.js
21210

191675
no rank
3

1
node395.members.0.js
no rank
36866

2
no rank
84563

no rank
146507
1

node398.members.0.js
1
1199245
species

1
genus
1906661

1
node400.members.0.js
species
1070130

genus
590
11

28901
species
node402.members.0.js
11
6

59201
subspecies
5

node404.members.0.js
1
no rank
119912

2
node405.members.0.js
108619
no rank
1

1
node406.members.0.js
877468
no rank

node407.members.0.js
1
no rank
54388

1
440524
no rank

no rank
866913
1
node409.members.0.js

570
genus
33

244366
species
node411.members.0.js
1

16
22
node412.members.0.js
573
species

subspecies
72407
6
node413.members.0.js

node414.members.0.js
1
species
1463165

species
1905288
node415.members.0.js
4

571
species
node416.members.0.js
3

2
node417.members.0.js
2026240
species

genus
1335483
1

1
species
563

630626
no rank
1
node420.members.0.js

2
genus
544
node421.members.0.js
7

1
3
node422.members.0.js
1344959
species group

1
node423.members.0.js
species
546

species
2066049
1
node424.members.0.js

2
node425.members.0.js
species
545

5
160674
genus

species
575
node427.members.0.js
4

1
node428.members.0.js
54291
species

genus
1330546
2

1334193
species
node430.members.0.js
1

1
node431.members.0.js
species
61647

179
genus
413496
node432.members.0.js
9375

2
species
1163710

2
node434.members.0.js
no rank
1073999

2
species
535744

2
node436.members.0.js
1074000
no rank

8144
species
28141
9171
node437.members.0.js

node438.members.0.js
919
no rank
1138308

47
node439.members.0.js
290339
no rank

node440.members.0.js
61
956149
no rank

9
413502
species

no rank
693216
9
node442.members.0.js

3
species
413501

1159613
no rank
node444.members.0.js
3

node445.members.0.js
6
413503
species
4

1159491
no rank
node446.members.0.js
2

3
species
413497

3
413498
subspecies

node449.members.0.js
3
1159554
no rank

561
genus
53

node451.members.0.js
53
species
562
41

no rank
405955
node452.members.0.js
3

1
node453.members.0.js
no rank
199310

2
1038927
no rank

1048254
no rank
node455.members.0.js
1

no rank
1134782
node456.members.0.js
1

2
1603259
no rank

node458.members.0.js
2
331111
no rank

1
node459.members.0.js
no rank
1446746

1
no rank
861906

216592
no rank
node461.members.0.js
1

no rank
585057
node462.members.0.js
1

no rank
2048777
1
node463.members.0.js

1
genus
83654

1
node465.members.0.js
species
1920116

1330547
genus
node466.members.0.js
2
1

node467.members.0.js
1
species
208223

genus
158483
1

1
node469.members.0.js
species
158822

547
genus
node470.members.0.js
11586
188

1
node471.members.0.js
1914861
species

399742
species
node472.members.0.js
2

node473.members.0.js
1
species
1868135

species
1166130
node474.members.0.js
1

1560339
species
node475.members.0.js
4

species
1977566
1
node476.members.0.js

3765
node477.members.0.js
11388
species group
354276

16
node478.members.0.js
species
1915310

1279
node479.members.0.js
1463
species
550

node480.members.0.js
2
1333850
no rank

108
subspecies
69219

no rank
1104326
108
node482.members.0.js

1
53
node483.members.0.js
subspecies
336306

716541
no rank
36
node484.members.0.js

no rank
1211025
node485.members.0.js
16

1045856
no rank
node486.members.0.js
7

node487.members.0.js
14
no rank
1354030

node488.members.0.js
13
208224
species

5133
node489.members.0.js
158836
species
446

3806
node490.members.0.js
299766
subspecies

129
node491.members.0.js
1812934
subspecies

node492.members.0.js
7
301102
subspecies

301105
subspecies
node493.members.0.js
630

node494.members.0.js
115
1296536
subspecies

species
299767
node495.members.0.js
15

species
2077137
node496.members.0.js
7

208
node497.members.0.js
61645
species
51

no rank
1421338
node498.members.0.js
24

no rank
640513
node499.members.0.js
133

69218
species
node500.members.0.js
189

46
node501.members.0.js
species
1812935

526
node502.members.0.js
species
2027919

node503.members.0.js
7
species
2077136

genus
929812
1

species
929813
1
node505.members.0.js

1
genus
1048757

1
node507.members.0.js
species
1048758

1
1903409
family
node508.members.0.js
5

3
node509.members.0.js
53335
genus
1

1
node510.members.0.js
species
1235990

species
470934
1

712898
no rank
1
node512.members.0.js

1
2100764
genus

species
665913
1
node514.members.0.js

order
135613
1

1
1046
family

1
genus
1980513

node518.members.0.js
1
1630141
species

135618
order
1

1
family
403

1
genus
39773

1
species
271065

node523.members.0.js
1
1091494
no rank

phylum
508458
1

1
649775
class

order
649776
1

family
649777
1

49894
genus
1

1
97477
species

node530.members.0.js
1
891968
no rank

node531.members.0.js
45761
1783272
no rank
3

1
phylum
1297

1
class
188787

order
118964
1

1
183710
family

genus
1298
1

1
node537.members.0.js
980427
species

12762
phylum
201174

4
node539.members.0.js
12761
1760
class

85009
order
4

31957
family
3

1
72763
genus

1909732
species
1
node543.members.0.js

genus
1912216
2

species
1747
2
node545.members.0.js

1
family
85015

genus
53387
1

species
546871
node548.members.0.js
1

order
85010
3

2070
family
3

1
node551.members.0.js
genus
40566

1
genus
1851

1
species
40988

node554.members.0.js
1
no rank
882081

1
genus
142577

530584
species
node556.members.0.js
1

85008
order
1

1
family
28056

1
1865
genus

species
196914
1

no rank
1246995
node561.members.0.js
1

1
85004
order

1
family
31953

1
genus
1678

species
28025
1
node565.members.0.js

85011
order
16

16
2062
family

1
genus
228398

1
node569.members.0.js
2126346
species

3
node570.members.0.js
15
1883
genus

2
379067
species

no rank
749414
2
node572.members.0.js

362257
species
node573.members.0.js
1

1
node574.members.0.js
47763
species

1912
species
2
node575.members.0.js

2049881
species
node576.members.0.js
1

species
1938841
node577.members.0.js
1

1901
species
1
node578.members.0.js

node579.members.0.js
1
species
1616117

1
node580.members.0.js
species
1736046

node581.members.0.js
1
species
2059884

node582.members.0.js
8817
order
85007
1

1762
family
3

1866885
genus
1

species
1804
node585.members.0.js
1

1
node586.members.0.js
1763
genus

1
670516
genus

node588.members.0.js
1
species
1520670

8812
family
1653

genus
1716
8812
node590.members.0.js
462

node591.members.0.js
8315
1718
species
7736

no rank
1079988
node592.members.0.js
111

no rank
1232383
node593.members.0.js
2

4
node594.members.0.js
no rank
1232384

43
node595.members.0.js
196627
no rank
22

node596.members.0.js
21
1204414
no rank

node597.members.0.js
13
no rank
1232381

node598.members.0.js
406
340322
no rank

1
152794
species

1
node600.members.0.js
no rank
196164

1
node601.members.0.js
161899
species

species
161896
2
node602.members.0.js

108486
species
1

node604.members.0.js
1
1451189
no rank

1
species
160386

no rank
1285583
1
node606.members.0.js

1652495
species
14
node607.members.0.js

species
1719
node608.members.0.js
3

species
191610
1
node609.members.0.js

species
1072256
1
node610.members.0.js

1408191
species
2

2
node612.members.0.js
931089
no rank

1
1717
species

698972
no rank
node614.members.0.js
1

species
38288
2

585529
no rank
2
node616.members.0.js

1721
species
2

no rank
1121353
2
node618.members.0.js

node619.members.0.js
1
1724
species

node620.members.0.js
2
species
35755

1
family
85025

1
1817
genus

species
37329
1
node623.members.0.js

85006
order
3915
node624.members.0.js
2

1
family
1268
3911
node625.members.0.js

3910
1269
genus

3516
1270
species
node627.members.0.js
3910

394
node628.members.0.js
no rank
465515

85023
family
2

518733
genus
1

species
412690
node631.members.0.js
1

337004
genus
1

279828
species
node633.members.0.js
1

1
84998
class

1
1643822
order

1
1643826
family

1
644652
genus

species
1335613
1
node638.members.0.js

1
phylum
544448

1
31969
class

186328
order
1

33925
family
1

1
genus
46239

2151
species
node644.members.0.js
1

node645.members.0.js
32987
phylum
1239
1

7
186801
class

7
order
186802

family
543349
1

genus
2733
1

1
2734
species

no rank
292459
1
node651.members.0.js

2
2304686
family

2
genus
2304692

node654.members.0.js
2
species
1515

2
31979
family

1485
genus
2

species
84022
node657.members.0.js
1

1
node658.members.0.js
species
1534

family
186803
1

genus
1663717
1

node661.members.0.js
1
species
1679721

186807
family
1

1562
genus
node663.members.0.js
1

class
91061
32978
node664.members.0.js
1

3
1385
order
node665.members.0.js
32975

13405
186822
family

26
13405
node667.members.0.js
44249
genus

172713
species
node668.members.0.js
1

node669.members.0.js
1
species
1536775

node670.members.0.js
1
species
2069255

365617
species
1

1
node672.members.0.js
no rank
1268072

node673.members.0.js
1
species
1536770

1406
species
node674.members.0.js
1

189426
species
node675.members.0.js
13373

family
186817
12717

node677.members.0.js
12716
genus
1386
4267

3
node678.members.0.js
species
129985

2049935
species
2
node679.members.0.js

node680.members.0.js
1
species
2009331

8157
node681.members.0.js
species group
653685
444

52
node682.members.0.js
1938374
species subgroup
1

40
46
node683.members.0.js
492670
species

no rank
1458206
node684.members.0.js
6

species
1390
node685.members.0.js
5
4

no rank
1034836
1
node686.members.0.js

species
1423
54
node687.members.0.js
33

2
node688.members.0.js
86029
subspecies

subspecies
483913
3
node689.members.0.js

7
node690.members.0.js
10
135461
subspecies

node691.members.0.js
2
no rank
1404258

1
node692.members.0.js
1052588
no rank

subspecies
96241
6

no rank
655816
node694.members.0.js
2

node695.members.0.js
4
1052585
no rank

4
node696.members.0.js
5
1452
species

1239783
no rank
1
node697.members.0.js

1648923
species
node698.members.0.js
1222
1166

766760
no rank
node699.members.0.js
56

3726
6281
node700.members.0.js
species
1402

node701.members.0.js
2532
no rank
279010

23
node702.members.0.js
1126218
no rank

species
119858
99
node703.members.0.js

species
33932
2
node704.members.0.js

2
node705.members.0.js
35841
species

species
1547283
node706.members.0.js
2

node707.members.0.js
3
species
2093834

species
859143
1
node708.members.0.js

node709.members.0.js
2
264697
species

species
1856406
14
node710.members.0.js

species
1467
node711.members.0.js
1

species
1479
node712.members.0.js
3

1441095
species
node713.members.0.js
5

species
2026248
1
node714.members.0.js

species
1127744
1
node715.members.0.js

2
node716.members.0.js
421767
species

5
node717.members.0.js
1408
species
4

315750
no rank
1
node718.members.0.js

3
species
324767

1367477
no rank
3
node720.members.0.js

1398
species
node721.members.0.js
3

node722.members.0.js
1
352858
species

128
node723.members.0.js
species
1664069

98228
species
2
node724.members.0.js

node725.members.0.js
5
species
561879

species
1565991
1
node726.members.0.js

species
1402861
node727.members.0.js
3

species
1705566
1
node728.members.0.js

86664
species
node729.members.0.js
3

84
node730.members.0.js
86661
species group
11

5
species
1396
node731.members.0.js
46

2
node732.members.0.js
405532
no rank

288681
no rank
2
node733.members.0.js

1
node734.members.0.js
1454382
no rank

1003239
no rank
node735.members.0.js
35

node736.members.0.js
1
526977
no rank

node737.members.0.js
2
species
580165

1428
species
10
node738.members.0.js

1392
species
13
node739.members.0.js
11

2
node740.members.0.js
no rank
1392837

node741.members.0.js
2
species
64104
1

527000
no rank
1
node742.members.0.js

1
node743.members.0.js
1478
species

node744.members.0.js
2
species group
1792192
1

1
node745.members.0.js
species
1049581

species
1178537
1
node746.members.0.js

1
species
79885

1
node748.members.0.js
398511
no rank

node749.members.0.js
3
79883
species

1
genus
150247

33934
species
1
node751.members.0.js

6848
family
90964

6848
node753.members.0.js
genus
1279
71

1
1292
species

node755.members.0.js
1
1194526
no rank

node756.members.0.js
2
species
1715860

1
species
1282
node757.members.0.js
2

1449752
no rank
node758.members.0.js
1

1
node759.members.0.js
species
29379

1288
species
node760.members.0.js
2

node761.members.0.js
1
29384
species

node762.members.0.js
1
214473
species

6729
node763.members.0.js
6743
29385
species

14
147452
subspecies

no rank
342451
14
node765.members.0.js

node766.members.0.js
5
species
29382

node767.members.0.js
1
species
1286

11
species
1280
14
node768.members.0.js

3
node769.members.0.js
subspecies
46170

species
1283
node770.members.0.js
2

2
node771.members.0.js
species
246432

2
186818
family

1
genus
1569

1571
species
node774.members.0.js
1

1
648800
genus

1
node776.members.0.js
species
2048654

2
order
186826

family
1300
1

1301
genus
1

1
1349
species

no rank
218495
1
node781.members.0.js

1
family
33958

1
1578
genus

species
1007676
node784.members.0.js
1

class
1737404
1

order
1737405
1

family
1570339
1

1
genus
150022

1
species
1260

no rank
334413
1
node790.members.0.js

7
no rank
1798711

phylum
1117
7

1
subclass
1301283

order
1150
1

1
1892252
family

genus
35823
node796.members.0.js
1

1161
order
4

1
1162
family

genus
1177
1

1
92942
species

1
node801.members.0.js
1091006
no rank

3
1185
family

node803.members.0.js
3
genus
1186
1

species
938406
1

no rank
1973478
1
node805.members.0.js

32054
species
1

1
node807.members.0.js
no rank
1170562

2
1890424
order

2
1890426
family

2
node810.members.0.js
1129
genus
1

1
species
33070

no rank
1917166
1
node812.members.0.js

1
phylum
203691

203692
class
1

1
order
1643686

family
143786
1

genus
29521
1

52584
species
node818.members.0.js
1
