# Supplementary material for: Benchmarking the MinION: Evaluating long reads for microbial profiling
Source: Sci Rep. 2020 Mar 20;10:5125. doi: 10.1038/s41598-020-61989-x (PMC7083898; doi:10.1038/s41598-020-61989-x)

Read Length Histogram

Summary read length distribution

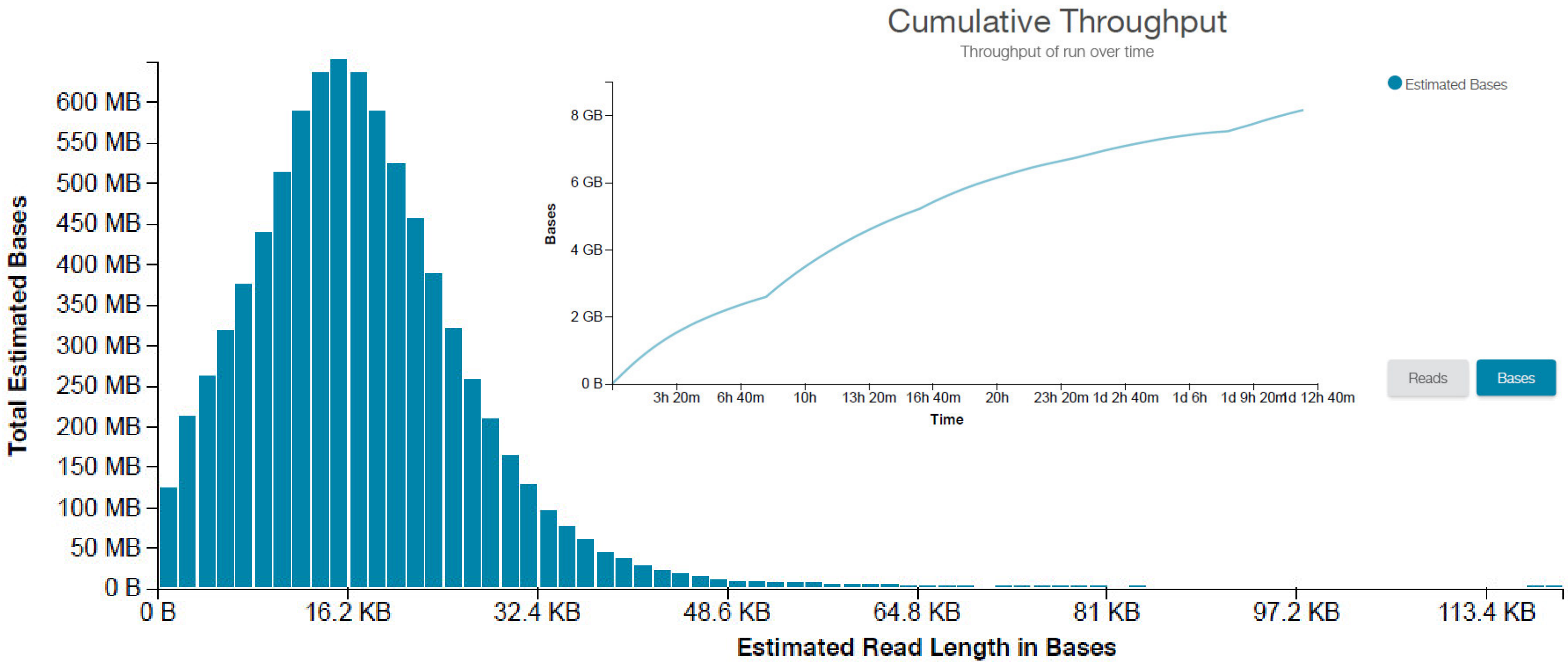

Supplement: Supplementary file 3 — Supplementary information3. [file 41598_2020_61989_MOESM3_ESM.zip › supplementary_figure_S1.pdf]
