# Supplementary material for: Benchmarking the MinION: Evaluating long reads for microbial profiling
Source: Sci Rep. 2020 Mar 20;10:5125. doi: 10.1038/s41598-020-61989-x (PMC7083898; doi:10.1038/s41598-020-61989-x)
Supplement: Supplementary file 3 — Supplementary information3. [file 41598_2020_61989_MOESM3_ESM.zip › supplementary_figure_S2.pdf]

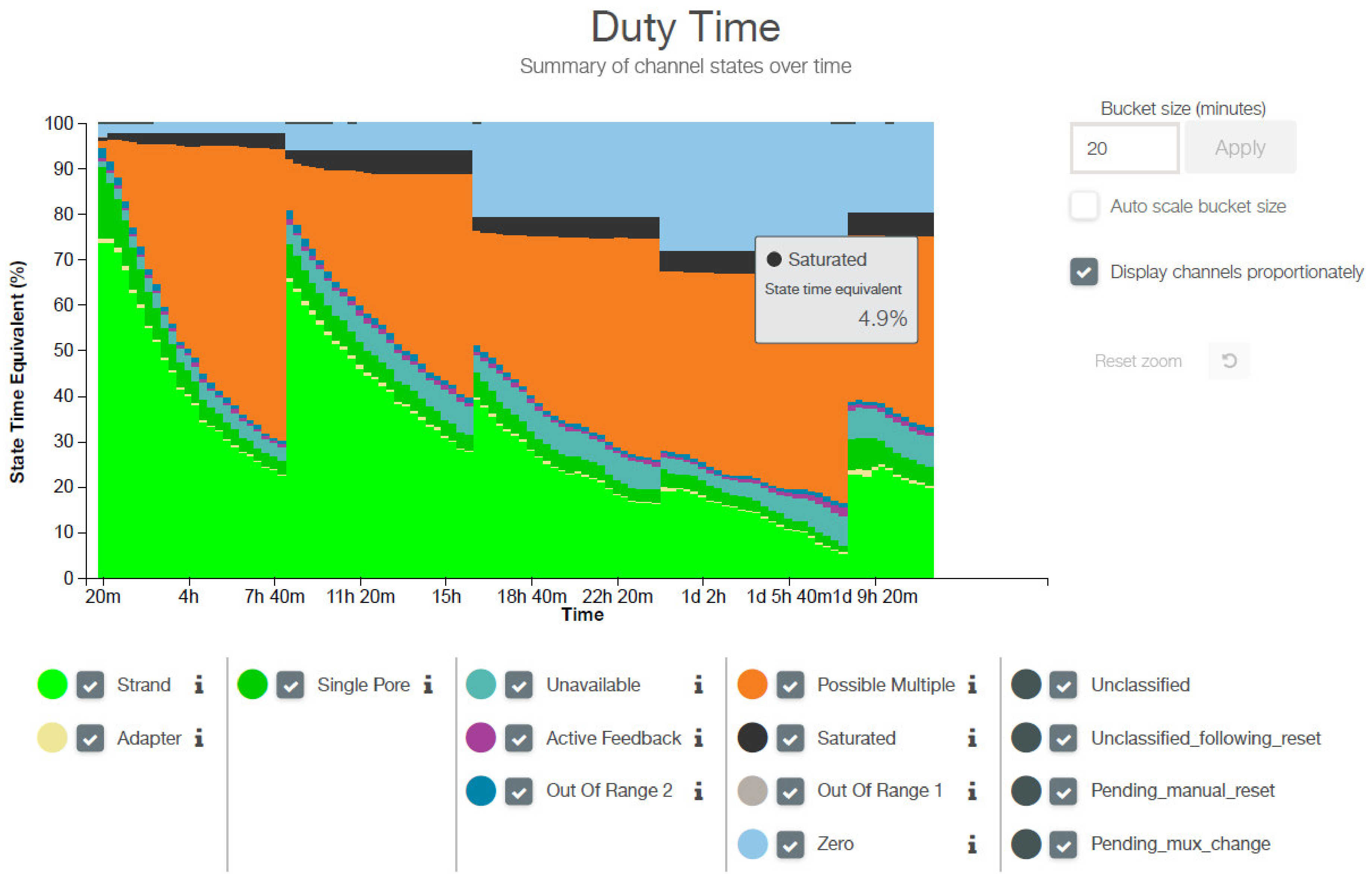
Duty Time
Summary of channel states over time
State Time Equivalent (%)
100
90
80
70
60
50
40
30
20
10
0
20m
4h
7h 40m
11h 20m
15h
18h 40m
22h 20m
1d 2h
1d 5h 40m
1d 9h 20m
Time
Saturated
State time equivalent
4.9%
Bucket size (minutes)
20
Apply
Auto scale bucket size
Display channels proportionately
Reset zoom
Strand
Adapter
Single Pore
Unavailable
Active Feedback
Out Of Range 2
Possible Multiple
Saturated
Out Of Range 1
Zero
Unclassified
Unclassified_following_reset
Pending_manual_reset
Pending_mux_change
