# Supplementary material for: Benchmarking the MinION: Evaluating long reads for microbial profiling
Source: Sci Rep. 2020 Mar 20;10:5125. doi: 10.1038/s41598-020-61989-x (PMC7083898; doi:10.1038/s41598-020-61989-x)
Supplement: Supplementary file 3 — Supplementary information3. [file 41598_2020_61989_MOESM3_ESM.zip › supplementary_figure_S4.pdf]

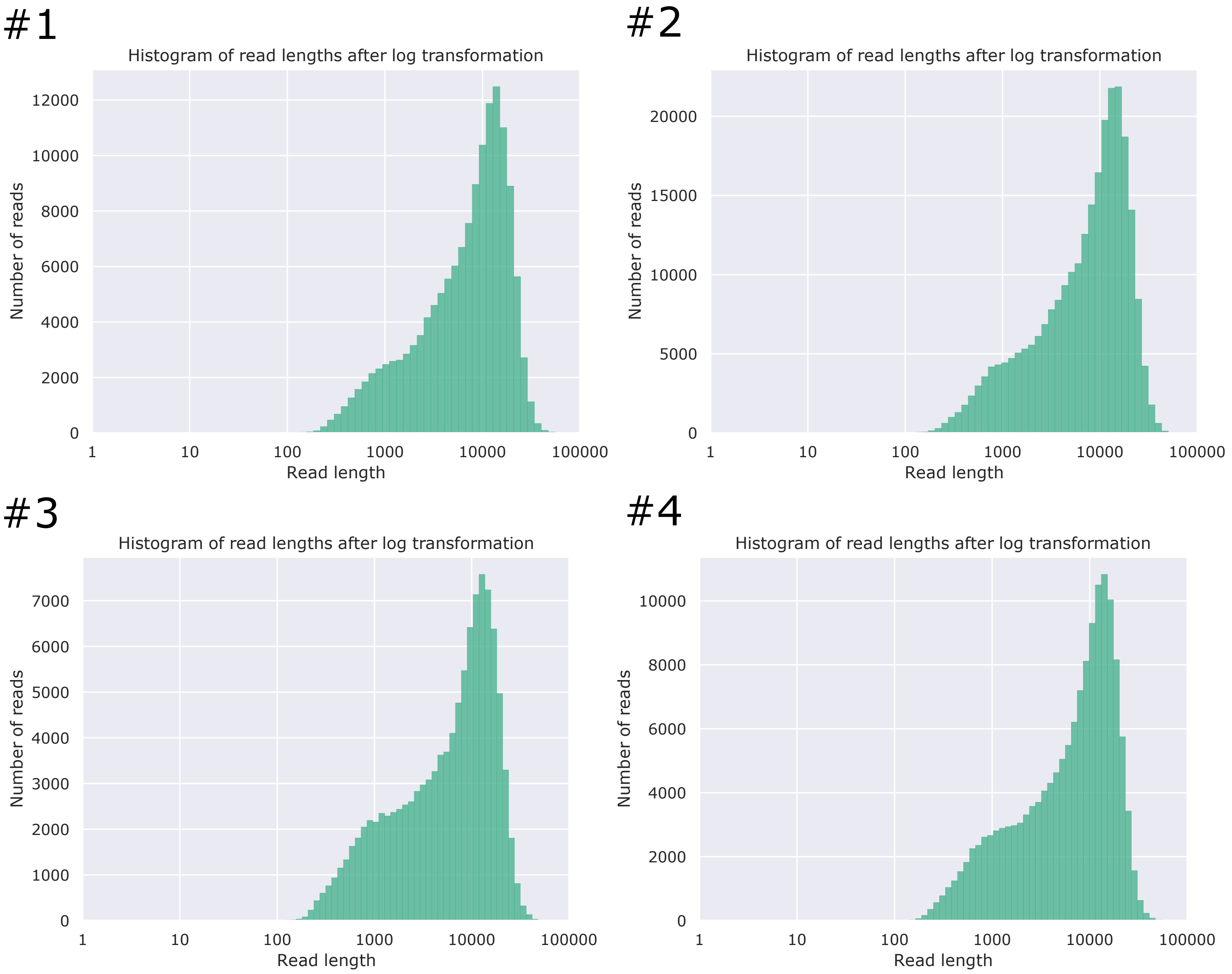
#1
Histogram of read lengths after log transformation
Number of reads
0
2000
4000
6000
8000
10000
12000
1
10
100
1000
10000
100000
Read length
#2
Histogram of read lengths after log transformation
Number of reads
0
5000
10000
15000
20000
1
10
100
1000
10000
100000
Read length
#3
Histogram of read lengths after log transformation
Number of reads
0
1000
2000
3000
4000
5000
6000
7000
1
10
100
1000
10000
100000
Read length
#4
Histogram of read lengths after log transformation
Number of reads
0
2000
4000
6000
8000
10000
1
10
100
1000
10000
100000
Read length
