# Supplementary material for: Benchmarking the MinION: Evaluating long reads for microbial profiling
Source: Sci Rep. 2020 Mar 20;10:5125. doi: 10.1038/s41598-020-61989-x (PMC7083898; doi:10.1038/s41598-020-61989-x)
Supplement: Supplementary file 3 — Supplementary information3. [file 41598_2020_61989_MOESM3_ESM.zip › supplementary_figure_S5.pdf]

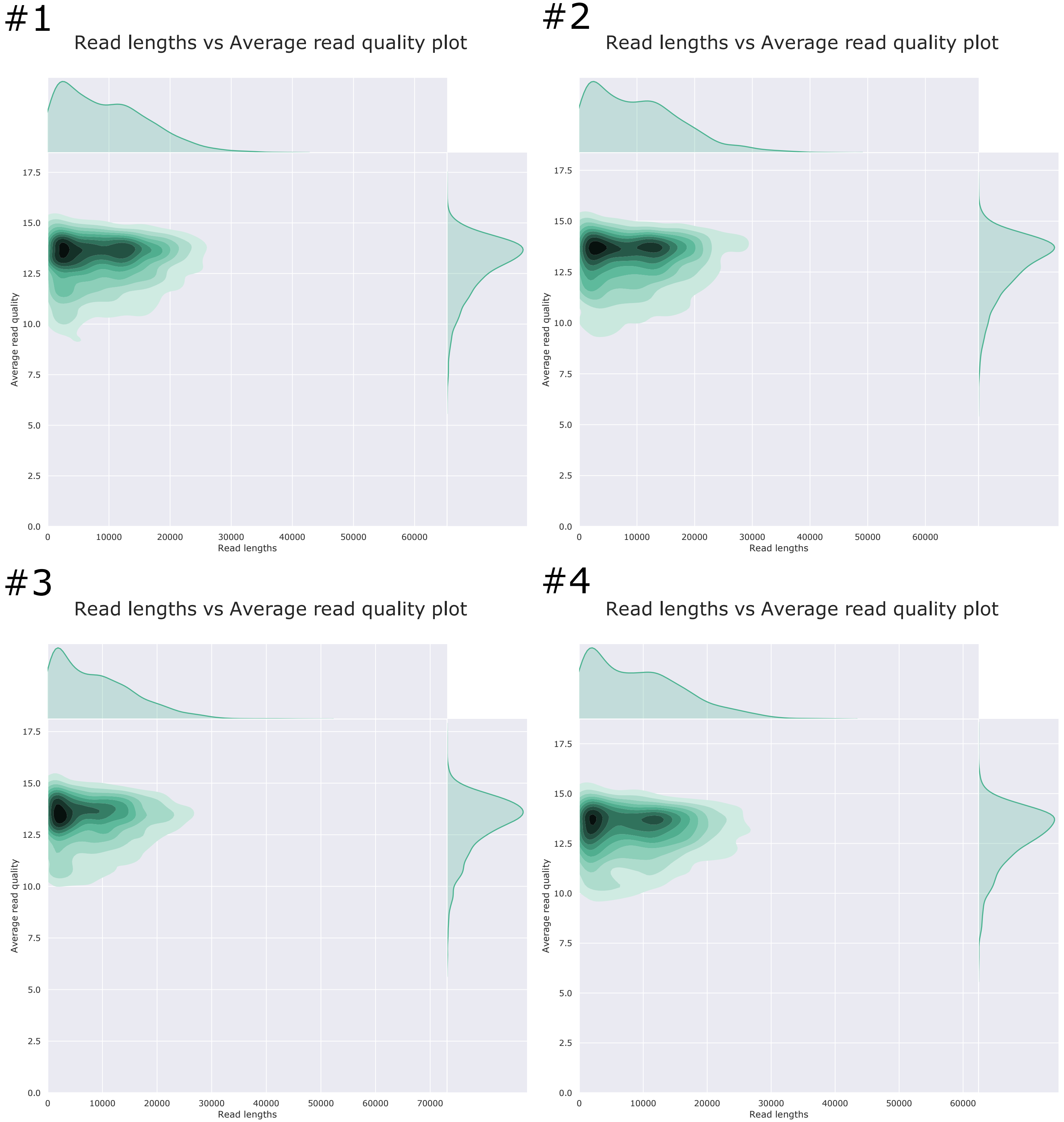

#1
Read lengths vs Average read quality plot
Average read quality
Read lengths
0 10000 20000 30000 40000 50000 60000
0.0 2.5 5.0 7.5 10.0 12.5 15.0 17.5
#2
Read lengths vs Average read quality plot
Average read quality
Read lengths
0 10000 20000 30000 40000 50000 60000
0.0 2.5 5.0 7.5 10.0 12.5 15.0 17.5
#3
Read lengths vs Average read quality plot
Average read quality
Read lengths
0 10000 20000 30000 40000 50000 60000 70000
0.0 2.5 5.0 7.5 10.0 12.5 15.0 17.5
#4
Read lengths vs Average read quality plot
Average read quality
Read lengths
0 10000 20000 30000 40000 50000 60000
0.0 2.5 5.0 7.5 10.0 12.5 15.0 17.5
